# Extracellular Vesicles in Head and Neck Cancer—A Systematic Review

**DOI:** 10.1002/jex2.70164

**Published:** 2026-07-17

**Authors:** Abdallah Swaid, Nadisha Piyarathne, Andrea Holme, David Muirhead, Gabriel Landini, Rasha Abu‐Eid

**Affiliations:** ^1^ School of Dentistry University of Birmingham Birmingham UK; ^2^ Institute of Dentistry University of Aberdeen Aberdeen UK; ^3^ Faculty of Dentistry Ibn Sina University for Medical Sciences Amman Jordan; ^4^ Institute of Medical Sciences University of Aberdeen Aberdeen UK; ^5^ Flow Cytometry STP Francis Crick Institute London UK; ^6^ School of Geosciences University of Aberdeen Aberdeen UK

**Keywords:** extracellular vesicles, exosomes, head and neck cancer, oral potentially malignant disorder

## Abstract

Head and neck cancer (HNC) is among the top ten malignancies worldwide and is associated with high morbidity and mortality. Oral potentially malignant disorders (OPMDs) predispose to certain HNCs. Recent studies suggest emerging roles of extracellular vesicles (EVs) in malignant transformation and provide potential in the management of HNC. This systematic review aimed to evaluate the role of EVs in HNC and OPMDs. The systematic review followed PRISMA‐2020 guidelines and was registered with PROSPERO. A comprehensive literature search was conducted in five electronic databases (Medline, Scopus, Embase, Cochrane and Web of Science), yielding 3705 initial records. Following de‐duplication and independent screening, 237 eligible articles were included. Data extraction was performed by two independent reviewers. Risk of bias was assessed using the Joanna Briggs Institute critical appraisal tools. Thematic analysis with narrative data synthesis was conducted and the results were organised under the following subthemes: EV isolation and characterisation methods, EV role in cancer and OPMDs progression, immune modulation, biomarker potential, and therapeutic applications. Studies included in the review were published between “1985–2025” and showed an accelerated growth in EV research after 2020. Most of the studies relied on traditional isolation and characterisation techniques including ultracentrifugation, transmission electron microscopy and flow cytometry. Most evidence was reported from preclinical studies and demonstrated EVs’ promise as biomarkers for early detection, prognostication, and therapeutic targets. However, the findings also underscored the need for standardised protocols. Current evidence on EVs’ role as drivers of epithelial mesenchymal transition, invasion, immune escape, metabolic reprogramming, and angiogenesis in the pathogenesis of HNC are summarised together with the role of EVs as biomarkers in HNC and potential applications in HNC therapy and therapy resistance. Mechanistic insights into the biological behaviour of EVs in HNC are provided and evidence‐based recommendations for future research are proposed in this review.

**Abbreviations**
CAF: cancer associated fibroblast
EMT: epithelial–mesenchymal transition
EVs: extracellular vesicles
HNC: head and neck cancer
HNSCC: head and neck squamous cell carcinoma
HUVEC: human umbilical vein endothelial cells
MS: mass spectroscopy
OSCC: oral squamous cell carcinoma
TEM: transmission electron microscope
WB: western blot

## 1 Introduction

Head and neck cancers (HNCs) are among the ten most common cancers worldwide (Mehanna et al. 2010; Mody et al. 2021). In the United Kingdom, approximately 34 new cases are diagnosed with around 16 deaths each day (Cancer Research UK 2025). Squamous cell carcinoma (SCC) accounts for over 90% of all HNCs (Kizhakkoottu et al. 2025; Markopoulos 2012; Mody et al. 2021), and more than half of HNC SCC cases are diagnosed at an advanced stage of the disease (Barsouk et al. 2023). HNC poses a substantial challenge as a public health concern due to compromise in vital functions such as speech, nutrition, aesthetic, psychosocial wellbeing, and economic status of the affected individuals, compromising the oral health related quality of life and wellbeing (Piyarathne, Dhanushka, et al. 2025; Piyarathne, Sinclair, et al. 2025).

Patients diagnosed at early stages have a significantly better prognosis and require less aggressive therapy, highlighting the need of new methods for early detection (Teppo and Alho 2008). One of the obstacles to early diagnosis is the inability to predict malignant transformation in a group of diseases that carry a higher risk of developing oral SCC, termed oral potentially malignant disorders (OPMDs), a term first established in 2005 following an expert meeting organised by the WHO Collaborating Centre for Oral Cancer in London (Warnakulasuriya 2020).

Identifying markers that can predict disease progression in OPMDs and detect HNC at early stages is needed to improve patient outcomes.

Extracellular vesicles (EVs) are heterogeneous, membrane‐enclosed particles released by cells, ranging in size from 30 to 1000 nm. EVs carry molecules such as nucleic acids, proteins, and lipids. Due to the variety of terminology of EVs, the International Society for Extracellular Vesicles (ISEV) recommends a classification based on observable characteristics such as size, density, surface markers, and mechanism of biogenesis, rather than relying solely on traditional terms like “exosomes” or “microvesicles,” which are often difficult to verify experimentally (Welsh et al. 2024). ISEV recommends that researchers describe EVs using measurable properties such as size ranges or specific markers, like “small EVs (<200 nm)” or “CD63+ EVs” (Welsh et al. 2024). While “exosomes” remains the most commonly used term for EVs in published literature, newer studies are beginning to follow the ISEV terminology recommendations.

EVs can be found in body fluids such as blood and saliva, and can be isolated from *in vitro* cell culture media. EVs may also help predict how the disease will progress (Zhang et al. 2015), and they could play a role in cancer therapy (Liu, Liao, et al. 2021; Zhang et al. 2015).

The field of EV research is expanding significantly and researching EVs in the context of HNC and OPMDs is no exception. This systematic review aims to synthesise current evidence on EVs in HNC, specifically oral and oropharyngeal cancers and OPMDs, with a focus on commonly used methods of EV isolation and characterisation, the role of EVs in HNC and OPMD progression and metastasis, their influence on the tumour microenvironment and immune modulation, the potential of EVs for early disease detection, as diagnostic and prognostic biomarkers and emerging research into their therapeutic applications.

## 2 Methodology

### 2.1 Protocol and Registration

This systematic review was conducted according to the PRISMA 2020 guidelines (Page et al. 2021). The protocol was registered in PROSPERO (CRD42024491923). The review question was defined according to SPIDER format, Sample: Head and neck cancer and OPMDs, Phenomenon of Interest: Extracellular vesicles, Design: observational, interventional, and experimental designs, Evaluation: EV and/ or their cargo and Research type: Primary research studies.

### 2.2 Data Sources

A comprehensive electronic literature search was independently conducted by two reviewers (AS and NP) in Medline (Ovide), Scopus, Embase, Cochrane and Web of Science databases. The original literature search was completed on the 24^th^ of January 2024. An updated search from the end point of the first search onwards was conducted on the 15^th^ of April 2025.

### 2.3 Search Strategy

**FIGURE 1:**
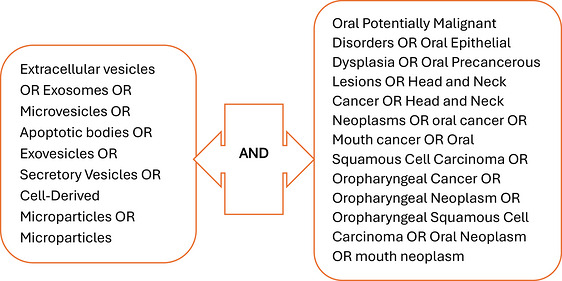
Search terms used for the database search.

The search strategy incorporated the following keywords: extracellular vesicles, exosomes, microvesicles, apoptotic bodies, exovesicles, secretory vesicles, cell‐derived microparticles, microparticles, oral potentially malignant disorders, oral precancerous lesions, oral epithelial dysplasia, head and neck cancer, head and neck neoplasms, oral cancer, oral squamous cell carcinoma, oropharyngeal cancer, oropharyngeal neoplasm, oropharyngeal squamous cell carcinoma, oral neoplasm and mouth neoplasm. These terms were combined with AND/OR to generate the search strategy. The search was conducted using the search terms in Figure 1, without time restrictions and with a filter to include studies published in the English language only.

### 2.4 Screening and Study Selection

All data records retrieved from the literature searches were exported and managed using EndNote referencing software. Duplicate records were removed using EndNote, followed by manual checks. Screening was then conducted in two stages (title and abstract followed by full‐text screening) using pre‐defined selection criteria by two independent reviewers (AS and NP), blinded to each other. The screening was conducted using the Rayyan.ai software. Any disagreements were resolved by discussion and if consensus could not be reached, a third independent reviewer (RAE) was consulted. The selection criteria for the studies were:

**Inclusion criteria**

- Primary research designs including cohort, case control, cross sectional, clinical trials, experimental, and animal studies.
- Studies focused on the role of extracellular vesicles in head and neck cancer, specifically oral and oropharyngeal squamous cell carcinoma, and oral potentially malignant disorders.

**Exclusion criteria**

- Case reports, case series, reviews, systematic reviews, letters to the editor and conference abstracts
- Articles where the full text was published in languages other than English.

**FIGURE 2:**
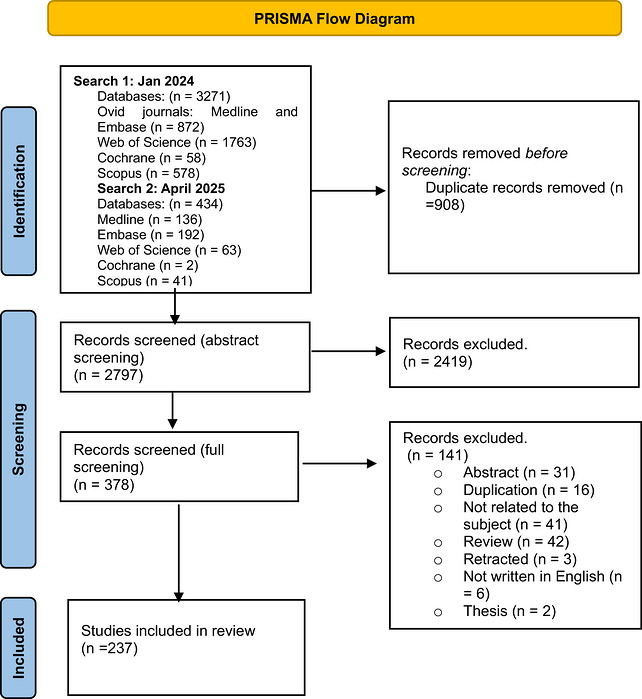
PRISMA diagram showing a summary of screening and study selection for the 237 included papers.

Screening and study selection are summarised in Figure 2, PRISMA (preferred reporting systems for systematic reviews and meta‐analysis) flow chart.

### 2.5 Reviewer Calibration

Two reviewers (AS and NP) extracted and analysed data from seven randomly selected articles for training and calibration. Cohen's kappa statistic was used to assess inter‐rater reliability, showing substantial agreement (0.65) between the two reviewers.

### 2.6 Data Extraction

Following calibration, data from all included full texts were extracted using a custom‐made spreadsheet in Microsoft Excel. Any disagreements were resolved by discussion, and if no agreement, with the involvement of an independent third reviewer (RAE). The following data items were extracted from each included article. First author, published year, country, study groups, diagnosis, HPV status, sample size of study groups, sample type, study design, site of lesion, age, sex, cell line, EV type studied, EV cargo, methods of EVs isolation, methods of EV detection and analysis, size/concentration of EV, shape and features of EV, EV biomarkers, role of EV, main findings/outcomes.

### 2.7 Risk of Bias Assessment

The risk of bias was assessed independently by two reviewers (AS and NP) for all included studies using appropriate Joanna Briggs Institute (JBI) critical appraisal checklists according to the study design. Studies were assigned a grade of Good (G), Fair (F) or Poor (P) quality according to pre‐defined criteria for each checklist. Grading was based according to the number of criteria that were satisfied in each tool as follows: cross‐sectional studies: 8–6 (Good), 5–3 (Fair), <3 (Poor); case‐control studies: 8–10 (Good), 4–7 (Fair), <4 (Poor), and cohort studies: 9–11 (Good), 5–8 (Fair), <5 (Poor).

### 2.8 Evidence Synthesis

Due to the heterogeneity of the included studies, a meta‐analysis was not considered. Evidence synthesis was conducted according to SWiM guidelines (Campbell et al. 2020). The results were summarised using descriptive statistics and tables. The narrative data synthesis was conducted under the following sub‐themes: General study characteristics, EV isolation and characterisation methods, EV role in HNC and OPMDs progression, immune modulation, biomarker potential, and therapeutic applications.

## 3 Results

### 3.1 Characteristics of the Included Studies

**FIGURE 3:**
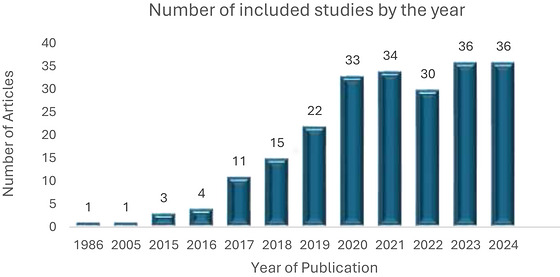
Number of included studies by the publication year, showing an increase over time.

The 237 included studies were published between 1986 and 2025, with the majority (76.0%) published after 2020. Figure 3 demonstrates the number of studies by publication year.

The majority of the included studies were laboratory‐based experimental studies (*n* = 166, 70.0%), while 71 studies (30.0%) utilised clinical samples, predominantly derived from blood (*n* = 45, 63.4%) and saliva (*n* = 26, 36.6%). Geographically, China, the United States, Japan, and Germany were the leading contributors, accounting for 118 (49.8%), 25 (10.5%), 15 (6.3%), and 14 (5.9%) of the included studies, respectively. The details of the data extracted from each study are presented in Table S1.

The risk of bias assessment of the included studies was conducted using the JBI critical appraisal tools. The results of the risk of bias assessment are presented in Table S2. Among the included studies, 79.3% of articles were rated as good quality with minimum risk of bias, 15.6% fair and only 5.1% were rated poor.

### 3.2 Methods for Isolation and Characterisation of EVs

Ultracentrifugation (UC) emerged as the predominant method for EV isolation, reported in 76.1% of the included studies, consistent with findings from an ISEV survey (Royo et al. 2020). UC was either used alone or in combination with other techniques such as filtration, size‐exclusion chromatography, and precipitation‐based kits. Although UC remains the gold standard due to its ability to isolate EVs based on size and density, it is time‐consuming and requires specialised equipment. To overcome these limitations, several studies employed hybrid protocols that combined different isolation strategies to enhance EV purity and yield (Guerreiro et al. 2018; Theodoraki, Hoffmann, et al. 2018). Comparative analyses demonstrated that two consecutive UC spins of 3 h each, yielded the highest particle to protein ratio and was identified as the preferred method for small EV isolation (Tengler et al. 2024). In contrast, the Biocompatible Magnetic Beads isolation method yielded over four times more EVs than UC (Sun et al. 2023). Approaches such as acousto‐fluidic platforms have enabled sensitive detection of human papillomavirus (HPV16) in salivary exosomes, offering potential for early, non‐invasive screening of HPV‐associated oropharyngeal cancer (Wang et al. 2020). Whole‐mouth saliva (WMS) sampling was found to be the optimal sample collection method for EV isolation from saliva because WMS is collected from the entire oral cavity and shows greater yield, EV concentration and broader biomarker coverage (Bozyk et al. 2023). Another study compared cost‐effective protocols combining exosome‐depleted serum, ultrafiltration, and size‐exclusion chromatography to reliably isolate high‐purity EVs from culture media (Guerreiro et al. 2018).

For EV characterisation, transmission electron microscopy (TEM), western blotting (WB), and nanoparticle tracking analysis (NTA) were the most frequently applied methods where 97.5% of the included articles used at least one of these three methods, aligning with the general workflow in EVs (Royo et al. 2020). Some examples of the use of these methods are described below. TEM provided visual confirmation of vesicle morphology and size, typically showing cup‐shaped structures (Li, Zeng, et al. 2024; Theodoraki et al. 2024). WB was widely used to detect EV‐specific markers such as CD9, CD63, and CD81, along with the absence of negative markers like calnexin or GM130 to assess sample purity (Hill et al. 2023). NTA enabled quantification and size distribution analysis of EVs in suspension, offering critical insights into particle concentration and homogeneity (Jiang et al. 2025). Several additional approaches complemented these standard techniques. Flow cytometry is considered as an important tool for EVs characterisation, applied to identify surface markers, cargo, and size; providing a robust quantitative data and enabling their profiling and functional assessment in biological fluids (Menck et al. 2017). Dynamic light scattering (DLS) was employed in some studies to provide rapid estimates of vesicle size distribution in suspension (Qiu et al. 2020). Furthermore, Raman‐based analysis of salivary exosomes demonstrated potential for non‐invasive oral cancer detection with reasonable sensitivity and specificity (Faur et al. 2023). In addition to these basic characterisation techniques, studies go deeper and focus on proteomic and transcriptomic analysis to gain deeper insight into EV cargo. Proteomic profiling, typically performed using mass spectrometry (Sun et al. 2023), enabled the identification of differentially expressed EV‐associated proteins linked to tumour progression, immune modulation, and potential diagnostic, prognostic, or even therapeutic value. Similarly, transcriptomic approaches—including small RNA sequencing and miRNA profiling—revealed EV‐derived nucleic acids that contribute to the molecular mechanisms underlying cancer progression and immune regulation (Hofmann et al. 2022). These transcriptomic signatures also demonstrated a strong potential as diagnostic and prognostic biomarkers. Together, proteomic and transcriptomic analyses provide deeper insight into the functional cargo of EVs, complementing conventional characterisation methods and advancing understanding of their role in cancer. These molecular analyses provided a high‐resolution view of EV content, complementing physical and phenotypic characterisation and advancing understanding of their functional roles in cancer. Together, these methods ensured robust validation of EV identity, integrity, and purity. Compared to the ISEV surveys, results of the studies included in this review show that EV work in HNC aligns with the general workflow of EVs in terms of common isolation and characterisation techniques.

### 3.3 Role of EV in HNC Progression and Metastasis

Several studies included in the review provide evidence on the EVs role in the progression and metastasis of HNC, primarily by mediating intercellular communication and remodelling the cancer microenvironment. Studies have shown that EVs carry active biomolecules such as Arg‐1, TGF‐β and PD‐L1, which affect tumour progression and could promote metastasis (Affolter et al. 2023; Han et al. 2023; Hofmann et al. 2023; Ludwig et al. 2023). EVs from HNC can induce epithelial–mesenchymal transition (EMT), enhance migration and invasion, promote angiogenesis, support invasiveness and metabolic reprogramming (Huang et al. 2023; Jiang et al. 2019; Sakha et al. 2016; Sun et al. 2019). A summary of EV cargos, and their roles in progression and metastasis of HNC is presented in Table 1.

**TABLE 1:** Role of selected EVs and their cargo in disease progression in HNC.

EV type/cargo	Mechanism/effect	Role in cancer progression and metastasis	Reference
Oncogenic miRNAs (miR‐1246 and miR‐29a‐3p)	EMT induction, invasion, migration, angiogenesis, apoptosis resistance	Direct and supportive: drives metastasis via EMT/invasion; apoptosis resistance supports survival	Cai et al. (2019), Sakha et al. (2016)
CAF‐derived EVs (miR‐3529‐3p, miR‐146b‐5p, miR‐382‐5p, loss of miR‐34a‐5p)	Migration, invasion, proliferation, metabolic reprogramming	Mixed role: migration/invasion direct; proliferation/metabolism supportive	He et al. (2023), Sun et al. (2019), You et al. (2023)
Immune‐modulating EVs (Arg‐1, TGF‐β, PD‐L1, miRNAs)	Macrophage polarization, MDSC expansion, immune evasion	Supportive: facilitates metastatic survival	Affolter et al. (2023), Cai et al. (2019), Hofmann et al. (2023), Li, Cao, et al. (2019), Ludwig et al. (2023), Theodoraki, Yerneni, et al. (2018), Yuan et al. (2021)
Metabolic EVs	Glycolysis alteration, fibroblast metabolic coupling	Supportive: provides energy for tumour growth and metastasis	Jiang et al. (2019)
Angiogenic EVs	Endothelial proliferation, tube formation; some inhibit angiogenesis	Mixed / context‐dependent: supports vascularisation for metastasis	Huang et al. (2023), Ludwig et al. (2020), Wang et al. (2022), Yan et al. (2021)

Several oncogenic miRNAs (e.g., let‐7c‐5p, miR‐1246, and miR‐29a‐3p) are enriched in tumour exosomes and contribute to EMT induction, enhanced motility, and apoptosis resistance (Cai et al. 2019; Li, Wang, et al. 2024; Sakha et al. 2016). These changes enable tumour cells to detach from the primary lesion, survive in hostile microenvironments, and colonise in distant tissues. HSP90‐high EVs further drive EMT, invasion, tumorigenesis, and macrophage reprogramming, effects that were suppressed by CDC37/HSP90α/HSP90β knockdown (Ono et al. 2020). In addition, EV‐associated adenosine and metabolites activate A2BR signalling, promoting endothelial reprogramming and angiogenesis (Ludwig et al. 2020).

EVs derived from Cancer Associated Fibroblast (CAF‐Ev) transfer regulatory miRNAs (e.g., miR‐3529‐3p, miR‐146b‐5p, miR‐382‐5p) while showing loss of tumour‐suppressive miRNAs such as miR‐34a‐5p, collectively promoting proliferation, metabolic reprogramming, and invasiveness (He et al. 2023; Sun et al. 2019; You et al. 2023). These vesicles also drive metabolic coupling between stromal and cancer cells, supplying metabolites that sustain glycolysis and biosynthesis, thereby providing energy for aggressive tumour growth (Jiang et al. 2019).

Promoting angiogenesis represents another critical but heterogeneous function of EVs. Multiple studies show that exosomal miRNAs such as miR‐205‐5p, miR‐130b‐3p, and miR‐210‐3p target regulators including PTEN, AMOT, and PIK3R1, thereby activating the PI3K/AKT signalling axis to enhance endothelial cell proliferation, migration, and tube formation (He, Zhang, et al. 2021; Huang et al. 2023; Wang et al. 2020; Yan et al. 2021). This vascularisation provides essential nutrients and access routes for tumour dissemination. Interestingly, not all angiogenic EV signals are pro‐vascular; stress‐induced EV cargos such as miR‐424‐5p can inhibit angiogenesis through LAMC1/Wnt/β‐catenin signalling, illustrating the context‐dependent nature of EV effects (Wang et al. 2022).

#### 3.3.1 Role of EV in HPV Driven HNC

Human papillomavirus (HPV), especially high‐risk types such as HPV‐16 and HPV‐18, plays an etiological role in oropharyngeal cancer (OPC). Emerging evidence indicates that HPV‐positive cancer cells release EVs that carry virus derived molecules, contributing to carcinogenesis, immune modulation and response to treatment. A summary of the evidence on EV's role in HPV associated HNC and OPMD is given in Table 2.

**TABLE 2:** Role of EV and their cargo in HPV associated HNC.

Sample source	EV cargo/molecule	Effect/role	Outcome	Reference
Saliva	Glycolytic enzymes	Metabolic reprogramming	Elevated glycolysis in HPV+ patients	Tang et al. (2021)
Cell line	miR‐9‐5p	Inhibits fibroblast activation (via NOX4)	Better prognosis	Wang et al. (2022)
Plasma / Saliva	Exosomal miRNA panels	Diagnostic biomarkers	Distinguishes HPV+ vs. HPV–	Hofmann et al. (2022), Mayne et al. (2020)
Cell line	miR‐9‐5p	Radiosensitivity, M1 macrophage polarization	Improved treatment response	Tong et al. (2020)
Cell line	miR‐1972	Dendritic cell modulation	Enhanced anti‐tumour immunity	Ludwig et al. (2020)

HPV positive HNC derived EVs carry HPV associated miRNAs, such as miR‐9 and miR‐1972 which influence the tumour microenvironment, immune response, and even treatment sensitivity (Hofmann et al. 2022; Ludwig et al. 2020; Mayne et al. 2020; Tang et al. 2021; Tong et al. 2020; Wang et al. 2022). For instance, exosomes carrying miR‐1972 can alter dendritic cell function, thereby enhancing antitumour immunity (Ludwig et al. 2020). Exosomal miR‐9‐5p inhibits TGF‐β–mediated fibroblast activation through NOX4, promotes macrophage M1 polarization, and increases radiosensitivity, which may explain the more favourable prognosis often observed in HPV‐positive patients compared to HPV negative oropharyngeal cancers (Tong et al. 2020; Wang et al. 2022). Notably, miR‐9 has emerged as one of the most consistently reported and functionally relevant cargos in HPV‐associated EVs. Consistently, HPV infection induces the secretion of miR‐9‐rich exosomes, which are associated with increased CD9 expression and enhanced radiation response in HNC through PPARδ downregulation (Tong et al. 2020). Studies have identified diagnostic panels of exosomal miRNAs in plasma and saliva, including miR‐9, capable of distinguishing HPV positive patients from HPV negative individuals and healthy donors (Hofmann et al. 2022; Mayne et al. 2020). Moreover, salivary EVs from HPV positive OPC patients have been shown to carry elevated glycolytic enzymes, suggesting that HPV infection may also reshape cancer metabolism through EV‐mediated signalling (Tang et al. 2021).

### 3.4 Role of EVs in OPMD

OPMDs such as oral leukoplakia, erythroplakia, oral submucous fibrosis (OSF) and oral lichen planus carry “a variable risk of transformation into OSCC”. Assessment of disease progression and risk of malignant transformation are crucial in the management of OPMD (Mello et al. 2020). In this context, EVs have emerged as promising tools for both early detection and the identification of therapeutic targets.

Exosomal ADAMTS9‐AS2 was identified as a suppressive factor in OSF microenvironment, with its downregulation associated with poor prognosis, making it a candidate biomarker for the transition into OSCC (Zhou et al. 2021). Exosomes from mesenchymal stem cells derived from oral leukoplakia, another OPMD, exhibit strong pro‐angiogenic effects by delivering high levels of MMP1, which enhances human umbilical vein endothelial cells (HUVEC) migration and angiogenesis (Li et al. 2022). These findings emphasise the role of EVs in malignant transformation of OPMDs.

As opposed to conventional therapies such as surgical and pharmacological management, EVs have been shown to be important as therapeutic approach for OPMD. A study shows that adipose stem cell (ADSC) derived EVs were able to suppress TGF‐β1‐induced collagen synthesis in oral fibroblasts by modulating the p38 MAPK signalling pathway, highlighting their therapeutic potential in reducing fibrosis in OSF (Liu, Li, et al. 2021). Additionally, ADSC‐EVs carrying miR‐375 were found to inhibit fibrosis in fibrotic buccal mucosal fibroblasts (fBMFs) by targeting FOXF1, thereby mitigating OSF progression through the miR‐375/FOXF1 axis (Han et al. 2021). The topical application of stem cell‑derived EVs carrying miR‐185 in a 7,12‐dimethylbenzanthracene (DMBA)–induced OPMD mouse model onto buccal lesions significantly reduced inflammation severity, decreased dysplasia incidence and lesion number, and lowered the expression of the proliferation marker PCNA and the angiogenesis marker CD31 in treated tissues (Wang et al. 2019).

### 3.5 Role of EVs as Regulators of Anti‐tumour Immune Responses

EVs play a critical role in modulating the immune system in HNC by either promoting immune evasion or immune activation. A particularly significant role of EVs is exerted through transferring immunoregulatory cargo. Immune‐suppressive EV cargo such as Arg‐1, TGF‐β, PD‐L1, and multiple immunoregulatory miRNAs facilitate macrophage polarisation toward an M2‐like phenotype, promote expansion of myeloid‐derived suppressor cells (MDSCs), and impair T‐cell cytotoxicity (Cai et al. 2019; Hofmann et al. 2023; Theodoraki et al. 2018; Yuan et al. 2021). This EV‐mediated immune evasion not only allows primary tumour survival but also enables metastatic spread under reduced immune surveillance. In this way, EVs reprogram immune responses in favour of tumour progression. Additionally, EV cargo such as TLR3 ligands from necrotic cancer cells and miR‐23a‐3p from M2 macrophages promote immune evasion and invasion and serve as independent predictors of recurrence and survival in OSCC (Li et al. 2023; Vasiljevic et al. 2023). EVs cargo facilitate cancer progression by enhancing immune evasion (Cai et al. 2019; Li et al. 2023) and allowing tumour cells to survive and disseminate (Sakha et al. 2016). In addition, metabolic EVs provide energy support for tumour growth (Jiang et al. 2019).

Regarding the modulation of innate immunity, EVs suppress natural killer (NK) cell activity by downregulating activating receptors such as NKG2D and delivering immunosuppressive molecules including PD‐L1, FasL, and TGF‐β, with levels correlating with disease stage and HPV negative status (Theodoraki, Yerneni, et al. 2018). EVs may have a dual effect on NK cells where short‐term exposure enhances cytotoxicity, while prolonged exposure reduces it (Zhu et al. 2020). Moreover, deletion of CHMP2A in HNC cells enhances NK‐mediated killing, underscoring the role of EVs in NK evasion (Bernareggi et al. 2022). As mentioned before, macrophage polarisation is another key target, where tumour derived EVs drive macrophages toward an M2 pro tumorigenic phenotype through cargos such as CMTM6 and ANLN‐210 acting via ERK1/2 and PTEN/PI3K/AKT signalling pathways (Guo, Mao, et al. 2021; Pang et al. 2021), or by carrying miR‐23a‐3p and miR‐29a‐3p (Cai et al. 2019). Exosomes from endoplasmic reticulum (ER) stressed OSCC or cancer stem cells transfer PD‐L1 and lncRNA UCA1, further supporting M2 polarisation (Wu et al. 2022; Yuan et al. 2022). In turn M2‐derived exosomes carrying miR‐23a‐3p enhance OSCC proliferation, migration, and resistance to apoptosis (Li et al. 2023). Conversely, M1‐polarised macrophages‐derived exosomes enriched with HOTTIP activate TLR5/NF‐κB signalling, inducing M1 polarisation and exhibiting anti‐tumour effects (Jiang et al. 2022; Xiao et al. 2018). Tumour‐Derived Exosomes enriched in TGF‐β also reprogram macrophages into a pro angiogenic, immunosuppressive state (Ludwig et al. 2022). EV mediated changes in monocytes, further induce pro tumorigenic inflammatory signalling (Momen‐Heravi and Bala 2018; Theodoraki et al. 2024).

As for the suppression of adaptive immunity, EVs impair dendritic cell differentiation, migration, and viability, thereby weakening antigen presentation (Silva et al. 2021), while tumour derived exosomes carrying galectin‐1 contribute to CD8+ T cell suppression (Maybruck et al. 2017).

The immunosuppressive impact of EVs is not limited to EV content, but surface molecules could affect the immune response. For example exosomes lacking CD45 (which is a surface marker for immune EVs) correlate with cancer aggressiveness while CD45+ hematopoietic exosomes are considered a marker for immune suppression (Beccard et al. 2020).

The role that EVs play in immune modulation provides opportunities for developing novel therapeutic strategies. Synthetic EVs like GT‐RGEV@CSFRi, demonstrate the potential to reprogram tumour‐associated macrophages from a pro‐tumour M2‐like state to an anti‐tumour M1‐like phenotype, thereby enhancing CD8^+^ T cell infiltration and activation within the tumour microenvironment while minimizing systemic toxicity. By delivering a targeted CSF1R inhibitor directly to macrophages, these engineered EVs selectively modulate the immune landscape, promoting anti‐tumour immunity without widespread off‐target effects (Zhou et al. 2024). Moreover, strategies like triple silencing of CDC37/HSP90α/HSP90β weaken EV transmission and M2 polarization, offering new immunotherapeutic solutions (Ono et al. 2020). These findings highlight the role of EVs in immune system modulation in HNC.

### 3.6 EVs as Biomarkers for HNC

EVs are an emerging source of biomarkers in HNC, due to their accessibility in body fluids and their ability to carry bioactive molecules that reflect tumour biology. Alterations in EVs cargo and surface molecules, concentrations, size and distribution have been correlated with disease progression and clinically relevant endpoints. Elevated exosome concentrations have been associated with lymph node metastasis, recurrence, and reduced survival in oral and oropharyngeal cancer (Hofmann et al. 2022; Hofmann et al. 2023; Ludwig et al. 2023; Theodoraki, Yerneni, et al. 2018). Elevated plasma exosome levels were linked with lymph node metastasis, in part via fibroblastic reticular cell activation through phosphorylated IFNGR1 and PD‐L1 induction (Han et al. 2023). These findings highlight their potential application as non‐invasive biomarkers for disease monitoring. Importantly, EV‐associated molecules such as exosomal TGF‐β and PD‐L1 often show stronger correlations with disease progression than conventional diagnostic markers, underscoring their translational potential (Affolter et al. 2023; Han et al. 2023; Ludwig et al. 2023).

Several studies have investigated the RNA cargo in EVs, including microRNAs(miRNA), long non‐coding RNAs(lncRNAs) and circular RNAs(lncRNAs). Across multiple studies, salivary and plasma‐derived EV miRNAs demonstrated diagnostic value for OSCC. For example, salivary EV‐associated miR‐486‐5p and miR‐10b‐5p correlated with tumour keratinisation and differentiation (Faur et al. 2022), while salivary miR‐24‐3p promoted OSCC proliferation via PER1 suppression (He et al. 2020). Cancer exclusive miRNA within exosomes from both plasma and saliva demonstrated diagnostic capacity in distinguishing HNC patients from healthy donors, as well as stratifying HNC by HPV status (Hofmann et al. 2022).

Exosomal miRNAs identified in HNC cell lines, including those that impair dendritic cell function, matched miRNAs found in patient plasma and could be promising diagnostic biomarkers (Silva et al. 2021). lncRNAs and circRNAs derived from EV cargo also show significant promise as prognostic biomarkers. For example, MAGI2‐AS3 and CCDC144NL‐AS1 in plasma EVs were linked with lymph node metastasis through the PI3K–AKT–mTOR pathway (Li et al. 2022). circRNAs such as circGDI2 and has_circ_0069313 have been reported as emerging diagnostic tools in OSCC (Zhang et al. 2020). More specifically, exosomal circGDI2 suppresses OSCC progression by sponging miR‐424‐5p and upregulating SCAI, thereby inhibiting tumour cell proliferation, migration, and invasion, making it a potential non‐invasive biomarker (Zhang et al. 2020). Conversely, has_circ_0069313 promotes immune evasion by sponging miR‐325‐3p to upregulate Foxp3 in regulatory T cells, facilitating OSCC immune escape, which highlights its value both as a diagnostic marker and a potential therapeutic target (Chen et al. 2022). Together, these circRNAs illustrate how exosome‐mediated delivery of regulatory RNAs can reflect tumour behaviour and modulate the tumour microenvironment. Plasma derived exosomal miR‐130a was significantly elevated in late‐stage OSCC and associated with poorer overall survival (He, Guo, et al. 2021). High MX1 expression levels and the presence of ANLN‐210 carried via salivary exosomes were associated with poor prognosis in HNSCC (Gong et al. 2021; Guo, Mao, et al. 2021). A group of GCGC motif miRNAs (miR‐24‐1, miR‐103b, miR‐127) promoted lymph node metastasis of OSCC by suppressing NK activity and demonstrated prognostic power with a hazard ratio (HR) of 2.98 (Li, Lin, et al. 2024). Circulating miR‐503‐3p has also emerged as a candidate prognostic biomarker, as extracellular vesicles derived from radioresistant OSCC cells can transfer miR‐503‐3p to recipient cells, promoting acquired radio‐resistance and potentially indicating poorer treatment response and clinical outcome (Yamana et al. 2022). Downregulation of ADAMTS9‐AS2 was associated with OSCC progression and poor prognosis (Zhou et al. 2021) while some markers showed both diagnostic and prognostic potential such as Exosomal miR‐21 (Liu et al. 2017).

Serum/Plasma EVs carried cancer‐associated proteins, including EGFR, EPHA2, cytokeratin 19, and Alix, which were elevated in OSCC and correlated with clinical stage and therapeutic response (Goudsmit et al. 2021; Nakamichi et al. 2021). Serum EVs containing squamous cell carcinoma antigen (SCCA) showed improved diagnostic sensitivity after vesicle disruption with saponin (Yang et al. 2022). Plasma proteomics from OSCC patients identified panels such as ApoA1, CXCL7, PF4V1, and F13A1 that predicted nodal metastasis (Li, Zhou, et al. 2019), and combined EVs‐protein signatures including CRP/VWF/LRG improved discrimination of OSCC cases and prediction of nodal involvement (Guo, Giang, et al. 2021).

EV surface profiling provided further diagnostic insights. OSCC‐derived EVs exhibited elevated N‐glycan structures, particularly in early‐stage disease (Wu et al. 2022). EV surface proteins EpCAM and CD45 differentiated OSCC from benign ulcers (Hong et al. 2022), while immuno‐enrichment of tumour‐associated EVs with markers like CD9, CD81, and low‐molecular weight proteins improved diagnostic specificity (Tamkovich et al. 2018). Salivary exosomes were found to contain protein profiles distinct from cancer tissues, with specific proteins like desmocollin‐2 enriched in pre‐dysplastic lesions and DNA repair‐related proteins depleted in malignant samples (Wang et al. 2022).

Other studies analysing EVs from smokers reported higher particle concentrations and larger vesicle size compared with non‐smokers, suggesting that smoking, which is a major risk factor for HNC, induces early vesicular alterations that may reflect cellular stress and contribute to oral cancer development, highlighting their potential as predictive biomarkers (Bano et al. 2023). Additionally, salivary exosome profiling using surface‐enhanced Raman scattering (SERS) combined with chemometric analysis achieved 75.9% sensitivity and 54.5% specificity in OSCC diagnosis, representing a promising liquid biopsy approach for OSCC diagnosis (Faur et al. 2023). While surgical biopsy remains definitive and is the gold standard diagnostic approach for OSCC, the SERS based exosome assay offers a reproducible, non‐invasive approach that could complement traditional methods, particularly for screening or longitudinal monitoring (Liu et al. 2024).

In addition to the diagnostic value, several EV‐associated proteins correlated with OSCC prognosis. Elevated serum exosomal LOXL2 levels were associated with low‐grade HNC, indicating its potential as an early diagnostic biomarker (Sanada et al. 2020). Elevated Tenascin‐C levels have been associated with increased tumour aggressiveness in HNSCC (Goudsmit et al. 2021), while elevated Alix levels in serum and salivary exosomes have been identified as potential biomarkers for OSCC, with serum exosomes Alix levels serving as predictors of therapeutic response (Nakamichi et al. 2021).

Postsurgical plasma exosome levels dropped after OSCC removal, but persistently high concentrations predicted early recurrence and poorer survival (Rodríguez‐Zorrilla et al. 2023). Table 3 summarises a list of EV associated molecules and their potential applications as diagnostic and prognostic biomarkers in HNC and OPMD.

**TABLE 3:** Diagnostic and prognostic applications of selected EV biomarkers in HNC and OPMD.

Cargo type	Biomarkers	Type of biomarker	Reference
miRNAs	miR‐486‐5p, miR‐10b‐5p, miR‐24‐3p, miR‐21, miR‐210‐3p	Dx	Faur et al. (2022), He et al. (2020), Wang et al. (2020)
lncRNAs/circRNAs	MAGI2‐AS3, CCDC144NL‐AS1, LBX1‐AS1, circGDI2, has_circ_0069313	Dx	Ai et al. (2021), Chen et al. (2022), Li et al. (2022), Zhang et al. (2020).
Proteins	EGFR, EPHA2, CK19, Alix, SCCA, ApoA1, CXCL7, PF4V1, F13A1, (CRP, VWF, LRG), EpCAM, CD45, CD9 and CD81, TGF‐β1	Dx	Goudsmit et al. (2021), Guo, Giang, et al. (2021), Hong et al. (2022), Huang et al. (2021), Li, Zhou, et al. (2019), Nakamichi et al. (2021), Tamkovich et al. (2018), Yang et al. (2022)
Glycans	N‐glycan (8 glycan structures)	Dx	Wu et al. (2022)
miRNAs	miR‐130a, miR‐21, miR‐503‐3p, GCGC motif miRNAs (miR‐24‐1, miR‐103b, miR‐127), miR‐210‐3p	Px	He, Guo, et al. (2021), Li, Lin, et al. (2024), Liu et al. (2017), Yamana et al. (2022)
lncRNAs/circRNAs	ADAMTS9‐AS2, has_circ_0069313	Px	Chen et al. (2022), Zhou et al. (2021)
Proteins	LOXL2, HSP90 family, Tenascin‐C, Alix	Px	Nakamichi et al. (2021), Ono et al. (2020), Sanada et al. (2020), Goudsmit et al. (2021)

Abbreviations: Dx, Diagnostic biomarker; Px, Prognostic biomarker.

### 3.7 EVs role in HNC Therapy and Resistance to Therapy

EVs are emerging as promising tools in cancer therapy. Their potential as therapeutic agents can be divided into two categories; native EVs with inherent tumour‐suppressive or tumour‐promoting properties that can be targeted and engineered or loaded EVs that can be used as delivery systems for drugs, RNAs, or other therapeutic molecules.

Native EVs represent the physiological, unmodified vesicles naturally secreted by tumour, stromal or immune cells. These vesicles can promote or suppress tumour progression depending on their origin and cargo composition. Some native EVs include tumour‐suppressive vesicles, such as lncRNA MEG3, which has been shown to suppress angiogenesis through the miR‐421/HS2ST1 axis (Huang et al. 2024), thereby restraining tumour vascularisation and progression. In contrast, tumour‐promoting EVs significantly contribute to cancer aggressiveness and therapy resistance. Examples include tumour‐associated macrophage (TAM)‐derived miR‐31‐5p which enhances invasion and metastasis (Yuan et al. 2021), tongue SCC‐derived miR‐205‐5p which promotes tumour growth and survival (Huang et al. 2023), CAF‐EVs that contribute to radio‐ resistance via miR‐503‐3p (Yamana et al. 2022). These findings demonstrate that native EVs play a key role in HNC progression and response to therapy and suggest that blocking oncogenic EVs could slow tumour growth and manipulating them could overcome resistance to chemotherapy or radiotherapy.

Engineered or loaded EVs are intentionally designed or modified to function as drug delivery systems, tumour suppressors, or immune modulators. Advances in bioengineering and nanotechnology have allowed the control of EV content or surface molecules to optimise therapeutic efficacy and target specificity. For instance, EVs loaded with miR‐34a (Deng et al. 2022) and LBX1‐AS1/miR‐182‐5p/FOXO3 exosomes (Ai et al. 2021), have demonstrated significant antiproliferative and pro‐apoptotic effects in preclinical HNC models, thereby suppressing tumour growth. Beyond direct tumour suppression, engineered EVs are increasingly recognised as powerful immunotherapy tools. Constructs such as EV PD‐L1 have emerged as important modulators of immune responses in HNC. PDL‐1–expressing EVs can suppress T cell activity and serve as prognostic markers in patients with OSCC patient treated with immune checkpoint inhibitors, linking EV‐mediated PD‐L1 expression to therapy response and clinical outcomes (Seki et al. 2024). In HPV+ HNSCC, The Dsg2/miR‐146a/IL‐8 pathway has been shown to play a key role in immune evasion through EVs that can transfer miR‐146a from tumour cells, which promotes IL‐8 production and correlates with nonresponse to neoadjuvant nivolumab, highlighting a mechanism by which EVs contribute to resistance to therapy (Hill et al. 2023). Hepatocyte growth factor–regulated tyrosine kinase substrate regulates the loading of PD‐L1 in EVs which contribute the immune evasion in OSCC (Xiao et al. 2023) illustrating how tumour‐derived EVs can modulate the immune microenvironment in HNC. Innovative approaches such as the (Hy‐M Exo nano vaccine) highlight the potential of EV‐based vaccines to induce robust anti‐tumour immune responses. The Hy‐M‐Exo vaccine works by using the exosomes to safely and efficiently deliver cancer antigens to the lymph nodes which activate the immune cells that can attack the cancer cells throughout the body and serving as predictive biomarkers for immunotherapy responsiveness (Xu et al. 2023). Table 4 provides an overview of native and engineered EVs including their cargo, mechanisms of action, and therapeutic implications in HNC.

**TABLE 4:** Role of EVs and their cargo in HNC therapy.

EV cargo	Mechanism/effect	Therapeutic or biomarker implication	Reference
Native EVs—Exosomal lncRNA MEG3	Regulates angiogenesis via miR‐421/HS2ST1	Tumour ‐promoting: Promotes tumour angiogenesis, a potential therapeutic target	Huang et al. (2024)
TAM‐derived exosomal miR‐31‐5p	Promotes OSCC proliferation	Targeting may inhibit tumour growth	Yuan et al. (2021)
Shh/RhoA proteins	Drive OSCC progression	Shh/RhoA inhibition as therapy serves as a potential therapeutic strategy to block tumour vascularisation	Xiao et al. (2017)
TSCC‐derived exosomal miR‐205‐5p	Enhances angiogenesis via AMOT	Anti‐angiogenic therapeutic target	Huang et al. (2023)
CAF‐derived sEV‐bound VEGF	Mediates bevacizumab resistance	Heparinase + bevacizumab restores sensitivity	Li et al. (2020)
Exosomal miR‐503‐3p	Induces radio resistance via BAK	Potential therapeutic target: Inhibiting miR‐503‐3p can reverse radiation resistance and improve radiotherapy efficacy	Yamana et al. (2022)
Engineered/ miR‐34a‐loaded exosomes	Suppress proliferation/migration/invasion (SATB2)	RNA‐based therapy Using engineered EVs as a targeted bio‐delivery vehicle to restore therapeutic miRNA‐34a levels	Deng et al. (2022)
LBX1‐AS1/miR‐182‐5p/FOXO3 exosomes	Blocks miR‐182‐5p to upregulate FOXO3 expression in OSCC cells, inhibiting Cancer suppression	Engineered tumour‐suppressive strategy by controlling tumour‐suppressive (M1) macrophage‐derived EVs to deliver anti‐tumour non‐coding RNAs	Ai et al. (2021)
EV PD‐L1	Poor survival; better ICI (immune checkpoint inhibitor) response	Biomarker for immunotherapy	Seki et al. (2024)
Dsg2/miR‐146a/IL‐8 pathway	Predictive of ICI response in HPV+ HNC	Biomarker panel for ICI treatment outcomes	Hill et al. (2023)
HRS (ESCRT protein)	Regulates sEV PD‐L1 secretion	Target to improve ICI efficacy	Xiao et al. (2023)
Hy‐M‐Exo (tumour exosomes + DC + CCR7)	LN targeting, T cell activation	Potent immunotherapeutic nano vaccine	Xu et al. (2023)

As mentioned above, EVs play a critical role in resistance to therapy in HNC, particularly mediating drug resistance and sensitisation. EVs contribute to chemoresistance by transferring proteins, miRNAs, and lncRNAs that modulate drug response, including cisplatin, 5‐fluorouracil, and targeted therapies such as erlotinib and bevacizumab. For example, ATP7B‐containing EVs and CAF‐derived EVs promote cisplatin resistance (Ogawa et al. 2024), while exosomal miR‐196a and macrophage‐derived EVs activate signalling pathways that reduce drug efficacy (Qin et al. 2019; Tomita et al. 2020). On the other hand, certain EVs such as circNR4A1‐containing EVs can sensitise OSCC cells to anticancer drugs by modulating pathways like K‐RAS/ERK and p53 (Dong et al. 2023). EV‐mediated mechanisms also have implications for immunotherapy and targeted therapy, including VEGF‐ or TGFβ‐containing EVs that influence chemotherapy response and chemoradiotherapy outcomes (Li et al. 2020; Rodrigues‐Junior et al. 2019). A summary of these EV‐mediated mechanisms, their cargo, and chemoresistance implications is provided in Table 5.

**TABLE 5:** Role of EVs and their cargo in chemoresistance in HNC.

EV cargo	Mechanism/effect	chemoresistance implication	Reference
EV‐mediated export of ATP7B	Promotes cisplatin resistance by exporting the drug out of cancer cells, lowering intracellular drug toxicity	Targeting EV release may enhance cisplatin efficacy by preventing drug export	Ogawa et al. (2024)
CAF‐derived exosomal miR‐196a	Targets CDKN1B and ING5, inducing resistance	Potential therapeutic target	Qin et al. (2019)
EV circNR4A1	Modulates K‐RAS/ERK and p53 pathways	Enhances sensitivity to anticancer drugs	Dong et al. (2023)
Plasma EV TGFβ	Associated with poor response to chemoradiotherapy	Targeting TGFβ signalling may improve outcomes	Ludwig et al. (2023), Rodrigues‐Junior et al. (2019)

EVs could have a role in mediating cellular responses to ionizing radiation. EVs released from irradiated cells can influence neighbouring non‐irradiated cells through a phenomenon known as the radiation‐induced bystander effect (Smolarz et al. 2022). This process involves the transfer of bioactive molecules such as proteins and microRNAs, leading to replication stress, altered apoptosis, and changes in gene expression in recipient cells (Smolarz et al. 2022). EVs from irradiated HNC cells have been shown to trigger early effects in target cells through ligand‐receptor interactions and carry proteins primarily associated with membrane structures, reflecting radiation‐induced cellular changes (Smolarz et al. 2022).

In OSCC, exosomal miR‐503‐3p has been implicated in promoting radio resistance by suppressing apoptosis via the BAK axis, with higher circulating levels correlating with poorer patient survival (Yamana et al. 2022). EVs released from clinically radioresistant cells also confer resistance to neighbouring cells, further supporting the role of the miR‐503‐3p–BAK axis in regulating radiation‐induced apoptosis and resistance in OSCC (Yamana et al. 2022).

These findings fit into the broader concept that exosomes and their miRNA cargo may serve as a link between radiation‐activated DNA damage response and inflammation‐related processes such as the senescence‐associated secretory phenotype when the cells enter a permanent growth arrest due to DNA damage induced by radiation (Abramowicz et al. 2020).

However, these findings should be interpreted with caution due to some limitations, such as lower purity and low homogeneity in exosomal preparations which means that non‐EV components, such as proteins, lipoproteins, or cellular debris, may contaminate the sample, which can interfere with downstream analyses and lead to misleading results. Low homogeneity refers to the presence of vesicles with highly variable sizes, cargo, or functional properties, making it difficult to draw consistent conclusions about specific EV function, biomarker reliability, or therapeutic potential, these factors limit the accuracy of experimental findings. Also, contamination from serum‐derived peptides is a concern (Welsh et al. 2024). Additionally, exposure to ionizing radiation significantly alters the protein composition of exosomes, with up‐ or downregulation of proteins involved in transcription, translation, cell division, and signalling, indicating that exosomal cargo reflects stress‐induced changes in cellular processes (Jelonek et al. 2015). Overall, exosomes serve as mediators of intercellular communication after radiation exposure, impacting tumour behaviour, immune modulation, and therapeutic outcomes.

Their impact however is not always favourable for the patient. EVs from radiated cells enhance cancer cells migratory capacity by activating the AKT pathway which support metastasis (Mutschelknaus et al. 2017). A study found that EVs released after radiation exposure can promote more aggressive, migratory behaviour in cancer cells leading to an increase in metastatic potential. Exosomes also mediate radiation‐ and chemotherapy‐induced PD‐L1 upregulation in HNC cells and EVs cargo, potentially contributing to treatment resistance and immune suppression (Affolter et al. 2023). Tumour cells can upregulate PD‐L1 in response to platinum‐based chemoradiotherapy, and this regulation is partly mediated by exosomes. By increasing PD‐L1 expression on both the tumour cell surface and within exosomal cargo, cancer cells may suppress T cell activity, promoting immune evasion and contributing to resistance to chemoradiotherapy (Affolter et al. 2023). In cisplatin‐resistant (cisRes) OSCC cells and patients, exosomal miR‐30a levels are downregulated, while Beclin1 expression is upregulated suggesting that the miR‐30a delivered by EVs could sensitise the cancer cells to cisplatin (Kulkarni et al. 2020).

Collectively, these findings underscore the central role of EVs and their molecular cargo in modulating response to therapy in HNC, influencing chemoresistance, radiosensitivity, immune interactions, and potential for recurrences.

## 4 Discussion

EVs are membrane bound nanoscale particles secreted by all cell types and are involved in intercellular communication via their cargo, which includes proteins, lipids, and nucleic acids. Their content reflects the physiological or pathological status of the originating cells, making them an attractive source of biomarkers for early detection, diagnosis, prognosis, and therapeutic intervention in cancer. The role of EV in HNC cancer is not well defined; therefore, this systematic review was conducted to find, collate and critically appraise published literature on the role of EV in HNC and OPMD. The literature search was conducted at two time points, to capture the most recently published articles in the field. The year‐wise distribution of included studies showed that there is a growing interest in this area of research. The included studies focused on several key themes, including EV isolation and characterisation techniques, the role of EVs in cancer progression and metastasis in HNC and OPMDs, immune modulation, EV potential as diagnostic and prognostic biomarkers, and their applications in therapeutic strategies.

Most of the studies used UC for the isolation of EVs, often in combination with other isolation methods to increase the purity. TEM, WB and NTA were the most commonly used characterisation techniques. The reported isolation and characterisation techniques align with the general research in EVs and specifications published by ISEV (Royo et al. 2020). However, the high variability in isolation and characterisation techniques continues to make comparisons across studies challenging. There is therefore a need to produce unified guidelines to ensure reproducibility across studies.

Beyond variability, the lack of harmonised isolation protocols has important biological and clinical implications. Differences in centrifugation speed, precipitation methods, filtration steps, and storage conditions could significantly alter EV yield, purity, and functionality. Consequently, reported biomarkers may reflect technical artefacts rather than true disease‐specific signatures. In addition, co‐isolation of lipoproteins, protein aggregates, and other non‐vesicular particles may confound downstream molecular analyses, particularly in plasma‐derived samples. Without standardised workflows and transparent reporting of pre‐analytical variables, cross‐study comparisons and external validation of candidate biomarkers remain challenging (Welsh et al. 2024).

EVs were reported to be players in cancer progression and metastasis by promoting tumour invasion or by supressing the anti‐tumour immune responses. Some EVs could play opposite roles by inhibiting cancer invasion or promoting cancer progression, their dual role depends on the EV source and cargo. Emerging evidence suggests that EV heterogeneity represents both a major challenge and an opportunity for clinical translation, as distinct EV subpopulations may carry unique diagnostic, prognostic, and therapeutic information (Carney et al. 2025). This context‐dependent behaviour underscores the biological complexity of EV‐mediated communication within the tumour microenvironment. Tumour‐derived EVs may promote angiogenesis, immune evasion, and metastatic niche formation, whereas EVs derived from stromal or immune cells may exert tumour‐suppressive functions. Such heterogeneity suggests that therapeutic strategies targeting EVs must consider cellular origin, molecular composition, and disease stage to avoid unintended effects.

One of the most significant challenges in HNC management remains the delayed diagnosis, with many patients presenting at advanced stages. Findings from this systematic review show that EVs may serve as promising diagnostic tools. Studies reported differential expression of EVs in plasma or saliva from OSCC patients compared to healthy individuals (Hofmann et al. 2022). An interesting finding was the identification of two distinct panels of cancer exclusive EV miRNAs from plasma and saliva that could discriminate HPV positive from HPV negative HNC patients, offering the potential for stratified diagnosis (Hofmann et al. 2022). Moreover, these markers may be specific to cargo within EVs, as free circulating miRNAs did not show similar diagnostic power, reinforcing the importance of EV‐enclosed molecules (Xu et al. 2022). Importantly, the superior diagnostic performance of EV‐enclosed miRNAs compared to free circulating miRNAs highlights the protective and stabilising function of the vesicular membrane in EVs. Encapsulation shields RNA cargo from enzymatic degradation, potentially enhancing detection sensitivity in biofluids such as saliva and plasma. This reinforces the rationale for prioritising EV‐associated biomarkers over whole‐fluid analyses in future diagnostic assay development.

In addition to diagnosis, EVs show promise in predicting prognosis. Several studies showed that EV cargo can reflect cancer aggressiveness, metastatic potential, and patient outcomes. For example, 90 kDa‐glycosylated LGALS3BP was found to be variably enriched in EVs from different OSCC cell lines, with higher levels observed in aggressive subtypes such as HSC3 and CAL27 (Cela et al. 2024). Our systematic review also highlighted the potential of EVs as biomarkers for predicting malignant transformation in OPMD. Several studies indicate that changes in EV content occur during malignant transformation. For example, EV‐associated RNAs, such as exosomal ADAMTS9‐AS2, have been implicated in dysplastic lesions and may represent early indicators of malignant transformation (Zhou et al. 2021).

EVs present innovative opportunities to revolutionise current therapeutic strategies for HNC. EVs derived from mesenchymal stem cells, especially those from adipose‐derived stem cells (ADSCs), have demonstrated anti‐fibrotic and anti‐inflammatory properties (Liu, Li, et al. 2021), which can be useful to halt disease progression in OSF. Additionally, EVs are being engineered for drug delivery, taking advantage of their biocompatibility and specific targeting properties via surface receptors, offering novel pathways for precision medicine and targeted therapy in HNC and OPMD.

The most recent systematic review specific to EVs in OSCC and OPMDs was published in February 2020, summarising 55 studies up to December 2019 (Yap et al. 2020). That systematic review provided a valuable early overview of EVs’ role in OSCC and OPMDs, highlighted EVs as multi‐functional players in cancer progression, with emerging implications for diagnostics, prognosis, and treatment avenues. Our systematic review covers the wealth of recent evidence that reflects the ever‐growing interest in studying EVs in HNC. Indeed, around 80% of all the identified literature about the topic was published after the date of the first systematic review.

Even with the promising evidence for the use of EVs in HNC, several limitations remain. A key limitation is the lack of standardised, reliable methods for isolating and characterising EV populations. Existing isolation techniques often produce EV preparations with suboptimal purity and yield, frequently containing variable amounts of protein aggregates and lipoproteins. These contaminants may originate from the cells themselves or from the culture medium and may or may not be associated with EVs (Welsh et al. 2024). Future research should prioritise standardising protocols for EV isolation and characterisation to improve reproducibility.

More research is needed to identify highly sensitive and specific EV biomarkers for HNCs and/or OPMD which could help in early detection, screening, risk assessment and prognosis. Longitudinal studies in OPMD patients assessing EVs as predictive tools for malignant transformation are also lacking. Increasing evidence suggests that single EV biomarkers may lack sufficient discriminatory power when used in isolation. Integrated biomarker panels that combine EV‐derived molecular signatures with established clinical and pathological variables may provide superior diagnostic and prognostic performance. Combining EV analysis, spatial multi‐omics, tissue architecture, HPV status, quantification of the tumour immune microenvironment (including immune checkpoint expression) with clinical data such TNM stage, smoking and alcohol history, radiomic imaging features could enhance the ability to predict malignant transformation and identify high risk patients. When incorporated into multimodal predictive models, these combined datasets demonstrate improved sensitivity, specificity, and risk stratification compared with single‐modality approaches. Such integrative strategies reflect a move towards precision oncology, where molecular and clinical data converge to guide personalised management decisions (Chen et al. 2023; Zandberg et al. 2024). Similarly, multimodal diagnostic approaches integrating molecular information from multiple biofluids have demonstrated promising accuracy in HNC detection. For example, fused Raman spectroscopic analysis of paired plasma and saliva samples achieved high diagnostic performance for HNC, highlighting the potential value of combining complementary liquid biopsy datasets with advanced analytical modelling approaches (Koster et al. 2022).

Artificial intelligence (AI) and advanced analytics play a central role in both the discovery and validation of EV biomarkers in HNC. Machine learning (ML) methods can identify complex, non‐linear patterns across EV cargo profiles and clinical covariates that traditional statistics often fail to capture. Recent studies have applied ML approaches to exosomal proteomics and RNA data, generating robust diagnostic and prognostic signatures with high accuracy. For example, a study identified exosome‐related gene signatures in HNSCC that stratified patients by prognosis and predicted drug sensitivity (Cai et al. 2025). Another study developed a six‐miRNA signature that achieved an AUC of 0.82 for stage classification in HNSCC patients and with strong survival stratification performance (Zhao and Cui 2020). Emerging AI‐driven approaches provide powerful tools for biomarker discovery, identification of therapeutic targets, patient stratification and multimodal model development, while advances in EV engineering are opening avenues for novel therapeutic applications (Cai et al. 2025; Kumar et al. 2020; Zhao and Cui 2020).

Future research should focus on large‐scale prospective validation of integrated biomarker panels, the development of standardised protocols for EV production and analysis and carefully designed preclinical and clinical studies to establish EVs not only as biomarkers but also as safe and effective therapeutic agents. This suggests a significant emphasis on preclinical research with the integration of patient‐derived specimens reflecting a promising translational shift from bench to bedside. The use of non‐invasive biospecimens such as saliva, urine and blood which are rich in EVs aligns with the broader push towards minimally invasive diagnostic approaches in cancer patients. Moving forward, translational efforts should prioritise large‐scale prospective validation of EV‐based biomarker panels across diverse populations. Longitudinal studies in OPMD cohorts are particularly critical to determine whether EV signatures can reliably predict malignant transformation before clinical or histopathological progression becomes evident. This has significant translational potential in improving patient outcome through early detection while a cure is still possible.

## 5 Conclusion

This systematic review highlights the multifaceted roles of EVs in HNCs and OPMDs, positioning them as active mediators of tumour biology and promising clinical tools. EV cargo reflects key processes including tumour progression, metastasis, immune modulation, and HPV‐associated disease stratification, supporting their potential utility in minimally invasive diagnosis and prognostic assessment. Despite ongoing challenges related to methodological variability and limited large‐scale validation, advances in multimodal biomarker integration, artificial intelligence–driven analytics, and EV engineering are accelerating progress toward clinical translation. With rigorous standardisation and prospective validation, EVs hold substantial promise not only as biomarkers but also as therapeutic agents and drug delivery platforms within precision oncology frameworks.

## Author Contributions

**Abdallah Swaid**: conceptualization (supporting), formal analysis (lead), methodology (lead), visualization (lead), writing – original draft (lead); review and editing (supporting). **Nadisha Piyarathne**: formal analysis (supporting), review and editing (supporting). **Andrea Holme**: review and editing (lead). Study supervision (supporting). **David Muirhead**: review and editing (lead). Study supervision (supporting). **Gabriel Landini**: Study supervision (supporting). **Rasha Abu‐Eid**: conceptualization (lead), formal analysis (supporting), methodology (supporting), writing – original draft (supporting), review and editing (lead). Study supervision (lead)

## Funding

AS is financially supported by a scholarship from Ibn Sina University for Medical Sciences.

## Conflicts of Interest

The authors declare no conflicts of interest.

## Supporting information




**Supporting Table 1**: Details of the 237 included manuscripts with the main findings. **Supporting Table 2**: Quality assessment of the 237 included manuscripts.

## Data Availability

Raw data generated from this study will be available upon reasonable request from the corresponding author.
