# Supplementary material for: Extracellular Vesicles in Head and Neck Cancer—A Systematic Review
Source: J Extracell Biol. 2026 Jul 17;5(7):e70164. doi: 10.1002/jex2.70164 (PMC13378098; doi:10.1002/jex2.70164)
Supplement: Supplementary file 1 — Supporting Table 1: Details of the 237 included manuscripts with the main findings. Supporting Table 2: Quality assessment of the 237 included manuscripts. [file JEX2-5-e70164-s001.docx]

| **Supplementary Table 1**: Details of the 237 included manuscripts with the main findings | | | | | | | |
| --- | --- | --- | --- | --- | --- | --- | --- |
| **Author and year** | **Patient/ animal samples** | **Cell lines** | **Sample type** | **EV isolation methods** | **EV detection and analysis methods** | **Exosomal biomarkers** | **Main findings** |
| (Ogawa, Ono et al. 2024) | HNSCC  7 Stage I  6 Stage II  8 Stage III  7 Stage IV  5 Normal tissues | HNSCC cell lines SAS, HSC3, HSC4 | cell culture supernatant | TER, UF | TEM, WB, PSD,  FM | CD9, EpCAM, HSP90 and GAPDH | ATP7B expression was markedly higher in advanced-stage and poorly differentiated HNSCC tissues and compared to early-stage and well-differentiated cases.  ATP7B-driven EV release contributes significantly to cisplatin (CDDP) resistance in HNSCC, identifying ATP7B as a potential therapeutic target. |
| (Tengler, Tiedtke et al. 2024) | 19 HNC patients  8 healthy donors | NA | saliva - stimulated using cotton swab | SEC,  dUC | TEM, NTA, WB, FCM. | CTLA-4, CD9, PD-L1, FasL, TGF-β+NKG2D+CD63, CD81 | Ultracentrifugation (UCopt: 3 h + 3 h) yielded the highest particle-to-protein ratio and was identified as the preferred salivary EV isolation method.  HNC patient salivary EVs particularly from late-stage cases had higher PD-L1, CTLA-4, and tetraspanin levels than healthy donors. PD-L1, FasL, and TGF-β may serve as diagnostic markers, while salivary EVs from both groups showed some immunosuppressive effects on NK and T cells. |
| (Cela, Alberto Caponio et al. 2024) | NA | OSCC cell linesHSC3, CAL27, HOC621, HSC2, HSC4, SAS,  A253 | cell culture supernatant | dUC | WB, IHC, qRT-PCR, ELISA | CD9, CD63 | LGALS3BP is overexpressed in OSCC and enriched in OSCC-derived EVs, promoting a more aggressive phenotype and representing a potential target for ADC-based therapy. |
| (Seki, Yamana et al. 2024) | 37 patients  patients with recurrent/metastatic oral squamous cell carcinoma  10 Controls | NA | serum of patients with Recurrent/Metastatic OSCC treated with ICI | NA | IHC, WB, NTA | CD9, PD-L1 | High PD-L1 EV levels were associated with poorer overall survival, although patients with higher levels tended to respond better to ICI treatment. |
| (Sun, Wang et al. 2024) | NA | CAL27, SCC25 | cell culture supernatant | UC | PCR, WB | FLAG, GAPDH | OSCC-derived exosomal miR-21 promotes angiogenesis by targeting RMND5A, whose inhibition enhances endothelial cell proliferation, migration, and tube formation via ERK and NF-κB activation. This pathway represents a potential therapeutic target for OSCC. |
| (Kang, Oh et al. 2024) | NA | UMSCC1  CVCL_7707)   FaDu CVCL_1218) | CAF from cell lines and primary cell culture | TER, UC | TEM, Confocal LSM, NTA | CD63 | CAF-P-derived EVs carrying hsa-miR-876-3p downregulated IGFBP3 and GATA1, enhancing cisplatin resistance, while anti-miR-876-3p restored with cisplatin sensitivity in OSCC. |
| (Wang, Zhou et al. 2023) | HNSCC specimens normal tissue specimens from precancerous tissues | FaDu, Cal27 | tissue and cell lines | UC | TEM, NTA, FCM, qpcr, WB | miR-21, α-SMA and FAP, actin | miR-21 was upregulated in HNSCC tissues and cell lines, and exosomal miR-21 from FaDu and Cal-27 cells induced CAF-like features in Human Bone Marrow Derived-Mesenchymal Stem Cells (hBMSCs), marked by increased FAP and α-SMA expression and altered cell cycle distribution. miR-21 inhibition suppressed this transition and reversed these changes, highlighting its role in HNSCC–stroma communication. |
| (Hofmann, Harasymczuk et al. 2023) | Cohort 1  (Tumour, Plasma) of 37HNSCC patients  10 age- and gender-matched HD  Cohort 2 (Exosomes)  20 HNSCC patients  8 age- and gender-matched HDs | PCI13 CVCL_C182) | blood plasma | SEC | TEM, NTA, WB, ELISA | Arg-1, TSG101, CD63, CD9 | high tumour Arg-1 exosomal expression was linked to favourable clinicopathology and longer RFS, whereas high plasma Arg-1 correlated with unfavourable features. Exosomal Arg-1 may serve as an accurate biomarker for lymph node metastases and represents a novel mechanism for intercellular enzyme transport promoting metastasis. |
| (Ludwig, Yerneni et al. 2023) | 36 HNCC patients  The 4-nitroquinoline 1-oxide (4-NQO) murine oral carcinogenesis model | NA | blood plasma | SEC | TEM, TRPS, WB | TGF beta, CD9, CD63 | In murine oral carcinomas progression correlated with increased tumoral, serum, and exosomal TGFβ levels, suggesting their potential as biomarkers. In HNSCC, circulating exosomes carry active TGFβ, which may provide a more clinically useful measure than tumour tissue or soluble TGFβ levels, as conventional measurements show poor correlation with progression or clinicopathologic data. |
| (You, Wang et al. 2023) | 26 tissue samples from OSCC patients. cancer and adjacent tissues | human oral mucosa fbroblasts (hOMFs), CAL 27 | cell culture supernatant | exoEasy Maxi kit | TEM, FCM nFCM | CD9, CD63, CD81, HSP90 and TSG101, miR-374c-3p | CAF‑Exos are internalized by OSCC cells and promote their proliferation, migration, invasion, and antiapoptotic capacity. MiR-3529-3p is abundant in CAF-Exos and is significantly higher than that in hOMF-Exos. Exosomes from tumour tissue had significantly higher miR-3529-3p expression than those from adjacent tissue.  This supports the in vitro findings and adds clinical relevance. |
| (Affolter, Liebel et al. 2023) | NA | UM‑SCC‑11B, UM‑SCC‑14C and UM‑SCC‑22B | cell culture supernatant | SEC | TEM, NTA | PD‑L1 and TRAIL | Radiotherapy, chemotherapy, and combined chemoradiotherapy upregulated PD-L1 expression in HNSCC cell lines and, in some cases, in their exosomes. Findings suggest tumour cell–mediated regulation of PD-L1 in response to platinum-based CRT. |
| (Liu, Li et al. 2023) | 30 OSCC Pt, 14 Control | UMSCC, CAL27,  CAF | Tissue and cell culture supernatant | UC | TEM | CD63, CD9, HSP70, | CAF-derived sEVs enriched in αLOX bound collagen I via integrin α2β1 and promoted collagen crosslinking, which induced EMT in OSCC through the FAK/paxillin/ROCK/YAP pathway in the cancer cell lines.  OSCC patient tissue samples were analysed to confirm that CAF markers (like αLOX) and collagen crosslinking are elevated in tumour stroma compared to normal tissue |
| (Ye, Duan et al. 2023) | NA | HN30, SCC15 | Tissue and cell culture supernatant | UC | TEM |  | Hypoxic HNSCC-derived exosomal miR-21 activates NFs into CAFs by targeting YOD1, thereby promoting tumour invasion and metastasis. |
| (Hill, Calder et al. 2023) | 28 HPV-positive HNSCC patients12HPV‑positive HNSCC patients | A431NC and A431XmiR146a cells | plasma and cell culture supernatant | ExoRelease™ technology | NTA | CD63, and the absence of the cis‐Golgi protein, GM130 and the housekeeping protein, GAPDH | The Dsg2/miR-146a/IL-8 pathway may serve as a predictive biomarker panel for ICI treatment outcomes in HPV-positive HNSCC.  Human samples provided clinical evidence plasma sEV miR-146a / IL-8 / Dsg2 correlate with ICI response in HPV+ HNSCC patients.  Cell lines and engineered A431 + UMSCC lines: used to demonstrate causality mechanism, that exosomal miR-146a can modulate IL-8 and influence cell viability, serving as a mechanistic proof that the miR‑146a/IL‑8 axis could underlie the clinical observations |
| (He, Guo et al. 2023) | OSCC tissues and normal oral mucosa tissues | HSC6 CAL33, SCC25 | tissue and cell culture supernatant | dUC | TEM, NTA | CD63, HSP70, CD9, and CD81 | CAF-derived exosomal miR-146b-5p promotes OSCC malignancy by targeting HIPK3, highlighting exosomal miRNAs as potential diagnostic biomarkers and therapeutic targets. |
| (Zhang, Guo et al. 2023) | Paracancerous tissues were normal oral mucosal tissues and connective tissues in the samples.  Streptozotocin-induced diabetic mice | HUVECs, HaCa, THFs | Tissue, cell culture supernatant | UC, SEC | BCA, TEM, NTA | CD9, CD6, CD81, GM130, b tubulin | Under MGO-induced diabetic stress, OSCC Tissue Exos, stimulate proliferation of fibroblasts, endothelial cells, and keratinocytes and enhance cell migration.  OSCC Tissue Exos accelerated wound healing (faster closure, better re-epithelialization, more angiogenesis) and  Improved collagen deposition and granulation tissue formation. |
| (You, Du et al. 2023) | NA | SCC9, SCC25, CAL27 | cell culture supernatant | F, UF | mass spectrometry | seven-gene PYGL  ACTN2  TSPAN15  EXT2  PLAU  ITGA5  HPRT1 | A seven-gene risk model identified HPRT1 and PYGL as key exosome-associated regulators linked to immune inhibition in HNSCC, though their roles in immune evasion require further study. |
| (Tengler, Schütz et al. 2023) | 32 HNC patients  12 No Evidence of Disease  16 HD | Human umbilical vein cells | venous blood | SEC | NTA, TEM | (CD9 and CD63), TSG101. Grp94 ApoA1 | Plasma sEV pro-angiogenic activity increases with HNC stage, due to more tumour-derived sEVs, making TEV-targeted anti-angiogenic strategies a potential therapeutic approach. When HUVECs are treated with plasma sEVs from higher-stage HNC patients, their angiogenic activity (e.g., proliferation, tube formation) is significantly increased. |
| (Feng, Xiao et al. 2023) | 75 OSCC patients 10 healthy | Cal27 | Saliva and cell culture supernatant | microfluidic chip, UC | TEM, FCM  DLS | Annexin V+ EGFR+ EV. | In saliva EGFR⁺ EV levels were found to be significantly higher in OSCC patients than in healthy controls, with the highest levels in late-stage disease.  The Annexin V⁺/Annexin V⁻ EGFR⁺ EV ratio in saliva may serve as a dynamic, non-invasive indicator of tumour progression.  The Cal27 cell line EVs reflect the clinical EV profiles, supporting mechanistic and functional studies. |
| (Li, Zhou et al. 2023) | NA | SSC7 | cell culture supernatant | UC | TEM, NTA, WB | .CD9, TSG101 and CD63, calnexin | OSCC-derived EVs promote tumour progression by regulating inflammatory cytokines and activating the IL-17A-induced signalling pathway |
| (Gonzalez-Callejo, Guo et al. 2023) | NA | mEER cell line model, MOC2 | cell culture supernatant | genetic modification (labelled EVs | FCM, IF | .CD63-eGFP -GFP | A novel platform was developed to track CSC-derived EVs in the TME under physiological conditions, revealing that tEV^CSC preferentially target MHC-II⁻ macrophages and PD-1⁺ T cells, mirroring immune subsets in the CSC niche and suggesting a role in immune modulation. |
| (Zhu, Yang et al. 2023) | NA | C3H and C57BL mice | cell culture supernatant | dUC | iTRAQ, SEM, DLS, ELISA | 90 K / CD63 and TSG101, but not albumin or calnexin in sEVs. | The knockdown of 90 K significantly inhibited the growth of xenograft tumour in immunocompetent C3H and C57BL mice, these results suggest that the immunomodulatory role of EV90 K depends on MDSCs rather than Tregs and that 90 K is required for the TDE delivery of miR21 to target cells. |
| (Theodoraki, Hofmann et al. 2023) | 25 HNC patients  10 normals | NA | blood | SEC | TEM, WB, FCM, NTA | CD63, CD9, TSG101, CD16+  exosomes. | CD16⁺ exosomes and CD16⁺ monocyte subsets may serve as liquid biomarkers in HNSCC, with changes in monocyte populations linked to altered plasma exosome size, reflecting patient immune status. |
| (Xiao, Wang et al. 2023) | 40 HNSCC patients and healthy donors | Cal 27, B16F10 | Tissue, plasma, and cell culture supernatant | UC | TEM, NTA, ELISA, FCM, WB | PD-L1 | increased sEV numbers and sEV PD-L1 distinguished patients from healthy donors. HRS, an ESCRT core protein, regulated sEV PD-L1 secretion, with knockdown reducing PD-L1 levels. HRS was linked to antitumor immunity and ICB response, making it a promising target to improve immunotherapy efficacy. |
| (Ludwig, Yerneni et al. 2023) | 520 primary HNSCC  44 normal controls | NA | plasma. | SEC, UF | TEM, TRPS, WB, BCA protein assay kit | CD63, CD9, Calnexin and Grp94 | HNSCC TMEs are enriched in TGFβ, and patient plasma exosomes carry higher TGFβ levels than those from healthy donors. Bead-assisted flow cytometry enabled quantification of exosome-surface TGFβ, though its use as a reliable progression biomarker remains challenging. |
| (Faur, Dinu et al. 2023) | 29 patient’s oral cavity oropharyngeal cancer  22 healthy volunteers | NA | saliva | UC | NTA, Raman | thiocyanate | Oral and oropharyngeal cancer patients had lower thiocyanate levels than healthy controls, with detection showing 75.9% sensitivity and 54.5% specificity using full-range spectra. This proof-of-concept study shows that SERS analysis of salivary exosomes, combined with multivariate chemometric methods, could serve as a promising liquid biopsy approach for early detection of oral and oropharyngeal cancer. |
| (Sun, Wang et al. 2023) | 30OSCC patients  30 healthy | NA | saliva | bi-functionalized magnetic beads (BiMBs)/UC | TEM, NTA, Western Blotting, and MS, | CD9, CD81, and TSG101 | BiMBs isolation yielded over four times more EVs than ultracentrifugation. HEP2, a known OSCC biomarker, showed the most sensitive surgical response, with decreased levels in all patients’ post-operation. |
| (Vasiljevic, Tarle et al. 2023) | 5 OSCC patients  3 mouse tumour grafts. | Detroit 562, FaDu, SQ20B | cell culture supernatant, tumour tissue | PureExo® Exosome Isolation kit | TEM, NTA, WB | TLR3, anti-CD63, calnexin | External irradiation was the most effective trigger for TLR3 activation, with TLR3 agonists carried in EVs. Activity was reduced by RNase or TLR3 blockade, indicating that endogenous ligands from the tumour can drive TLR3 signalling and potentially promote cancer progression without external ligands. |
| (Jha, Alam et al. 2023) | 150  OSCC patients tissue samples | f SCC4/9 cells, CisR SCC4/9 | cell culture supernatant | exoEasy Maxi kit | WB | PD-L1 | findings suggest that EV-associated PD-L1 may contribute to OSCC progression and immune modulation, and that targeting the Jak2–Stat3/MAPK–AP1 axis could reduce EV-mediated PD-L1 signalling for improved therapeutic outcomes |
| (Huang, Zeng et al. 2023) | NA | HSC3, SCC25 | cell culture supernatant | UC | TEM. NTA, WB, PCR | CD63, CD81, and TSG101. exosomal miR-205-5p | TSCC-derived exosomal miR-205-5p promotes HUVEC angiogenesis by targeting AMOT, suggesting its reduction could be a strategy for anti-angiogenic therapy. |
| (Xu, Liu et al. 2023) | NA | SCCVII, DC2.4, L929 | cell culture supernatant | UC | Confocal laser scanning microscope |  | A hybrid nanovaccine (Hy-M-Exo), combining tumor-derived exosomes with dendritic cell membranes, was developed for HNSCC. By incorporating CCR7, it efficiently targeted lymph nodes, activated T cells, and demonstrated strong therapeutic effects in mouse models, overcoming low immunogenicity and poor LN targeting of traditional dendritic cell vaccines. |
| (Bozyk, Tang et al. 2023) | three cohorts of participants: cancer-free healthy controls to Aim-1 (N ¼ 20), OPMD patients (N ¼ 20), and early-stage (I and II) OSCC patients (N ¼ 10). | NA | saliva | dUC, UC | TEM, WB, NTA, Mass Spectrometry | CD9-CD63+CD81 (AMER3, LOXL2 and AL9A1 | Salivary exosome concentration and size did not differ between healthy controls, OPMD, and OSCC patients. Two novel exosomal protein panels were identified to distinguish OPMD (AMER3, LOXL2, AL9A1) and OSCC (PSB7, AMER3, LOXL2), with whole-mouth saliva (WMS) sampling being the optimal isolation method. |
| (Wei, Sun et al. 2023) | OSCC patient | NA | saliva | Ti (IV)-functionalized magnetic beads and octa-arginine R8+ | Multiple Reaction Monitoring Cubed (MRM3) OR tandem Mass Spectrometry (MS/MS) | Phosphoproteomics, including FLNA, IBP-3, and S100-A9, | Salivary EVs showed differential phosphopeptide patterns among healthy, pre-surgery, and post-surgery OSCC patients, with phosphorylated alpha-amylase (AMY) significantly increased post-surgery. This study also introduced the first highly sensitive targeted MS method for detecting endogenous phosphoproteins in human saliva EVs. |
| (Han, Zhou et al. 2023) | Sixty fresh cervical LNs from 20 patients clinically diagnosed with T2-3 N0(4 HNSCC patients) | NA | fresh cervical LNs and cell culture supernatant | UC | TEM, NTA, Mass  Spectrometry, WB. | CD9, ALG-2-interacting protein X (Alix), CD81, and Rab5 | Cancer cell-derived EVs carrying IFNGR1 induce PD-L1 expression on FRCs via JAK1-STAT1/JNK1-STAT1 pathways, leading to CD8⁺ T cell exhaustion and creating an immunosuppressive pre-metastatic niche in sentinel lymph nodes, which promotes metastasis in HNSCC. Increased FRC proportion correlates with lymph node micro-metastasis. |
| (Li, Bao et al. 2023) | NA | CAL-27, THP-1 | Cell culture supernatant | dUC | TEM, NTA, WB | CD9, CD63 | M2 macrophage-derived exosomes promoteOSCC cell proliferation, invasion, and migration and inhibit OSCC cell apoptosis bytransferring miRNA-23a-3p into OSCC cells |
| (Zorrilla, Perez-Sayans et al. 2019) | 27 OSCC patients | NA | plasma | dUC | NTA |  | Surgical removal of OSCC significantly reduces plasma exosome levels, but elevated postsurgical concentrations predict early recurrence and poorer survival, supporting exosome measurement as a prognostic tool for clinical management. |
| (Juvkam, Zlygosteva et al. 2023) | NA | (E10 cells) | cell culture supernatant | UF, SEC | TEM, NTA, liquid chromatography– Mass Spectrometry | CD9 and CD81 within the e 100 most common proteins detected in EVs  according to the Vesiclepedia databa | X-ray irradiation of OSCC cells produced higher EV concentrations than proton therapy, while proton-derived EVs showed downregulation of key solute carrier proteins (SLC1A5, SLC7A5, SLC3A2), potentially impairing recipient cell growth and DNA repair. |
| (Chen, Gao et al. 2023) | NA | CAL27 and HSC3 human HNSCC | cell culture supernatant | UC |  |  | Fn OMVs can promote cancer metastasis to the lungs and Fn OMVs can promote autophagy. Treatment with CHQ to block autophagic flux inhibited cancer cell migration and invasion |
| (Galvão, Rode et al. 2023) | NA | OSCC cell lines-derived | cell culture supernatant | ExoQuick kit | NTA, RTqPCR | miR-92b-3p (A) and miR-103a-3p | EVPs from OSCC cell lines showed altered miRNA profiles compared to HaCaT cells, with upregulation of miR-20a-5p, miR-92b-3p, and miR-93-5p and downregulation of miR-31-5p, indicating changes in miRNAs targeting APP processing related to tumour aggressiveness. |
| (Bano, Vats et al. 2023) | (n=120) non-smoker’s control group (n=40) (n=40)  of individuals diagnosed with oral cancer tobacco consumers (smokers) (n=40) | NA | Saliva | Total Exosome Isolation™ | TEM, DLS, NTA, WB, FTIR | CD63 | Tobacco consumers and oral cancer groups showed significantly larger and more concentrated  exosomes compared to the healthy group |
| (Ludwig, Yerneni et al. 2022) | (The cancer genome atlas (TCGA) analysis)519 HNSCC 44 normal | NA | cell culture supernatant | SEC | TEM, TRPS, WB, MS | (Ludwig, Yerneni et al. 2023)Alix, CD63, and TSG101 as well as negative markers Calnexin and Grp94 carried by TEX | TGFβ⁺ tumour-derived exosomes (TEX) drive tumour progression by promoting TGFβ-dependent chemotaxis and activation of macrophages, reprogramming them toward a pro-angiogenic phenotype and functions. In a murine oral carcinogenesis model, TGFβ⁺ TEX enhanced tumour growth, underscoring the need to suppress TEX-mediated pro-tumour/angiogenic activities as a therapeutic strategy. |
| (Li, Guo et al. 2022) | Each group comprised 30 patients | NA | Serum exosomes | (SBI System Biosciences) | TEM, NTA, WB | CD9, CD66,63 and HSP70 | The expressions of the lncRNAs, namely MAGI2-AS3 and CCDC144NL-AS1, were significantly upregulated in rOSCC and OSCC-LNM. MAGI2-AS3 was overexpressed in cancer tissue compared to other control groups2. |
| (Wu, Ye et al. 2022) | 519 OSCC tissues, 44 normal tissues | Cal27, CSCs | Tissue and cell culture supernatant | differential centrifugation and ultracentrifuged. | TEM, NTA WB | CD81, CD63, calnexinCD63 and CD9 were highly expressed, but calnexin was not expressed on the surfaces of sEVs | OSCC cancer stem cell (CSC)-derived small extracellular vesicles (sEVs) enriched in lncRNA UCA1 induce M2 macrophage polarization by targeting LAMC2 via the PI3K/AKT pathway. This CSC-sEV-mediated modulation of the immunosuppressive microenvironment promotes OSCC cell migration, invasion, and tumorigenicity, highlighting CSC-sEVs and M2 tumour-associated macrophages as potential therapeutic targets. |
| (Zhuang, Wang et al. 2022) | NA | CAL 27 and SCC-9 | cell culture supernatant | exosome concentration solution (ECS) kit | TEM, NTA and WB | expressed CD81, CD63 and TSG101 | Phenformin suppresses angiogenesis in OSCC by modulating miR-1246 and miR-205 levels in tumour cell-derived exosomes, indicating its potential to reshape the tumour microenvironment and inhibit OSCC growth. |
| (Hofmann, Abou Kors et al. 2022) | 11 HNSCC patients and five healthy donors (HD) | NA | serum and saliva | dUC | TEM, NTA, WB | expressed CD81, CD63 and TSG102 | A first-of-its-kind comparison of plasma- and saliva-derived exosomal miRNAs in HNSCC identified 29 tumour-exclusive miRNAs (linked to TP53, TGFB1, PRDM1, FOXO1, and CDH1 signalling), leading to two biomarker panels with strong potential for liquid biopsy applications in distinguishing HNSCC patients from healthy individuals, and for assessing HPV status and UICC stage. |
| (Deng, Meng et al. 2022) | NA | HN6 OSCC cells | cell culture supernatant | dUC | TEM, NTA, WB | CD9, TSG101 | Cholesterol-modified miR-34a can be efficiently loaded into HEK293T-derived exosomes via coincubation, and these miR-34a-loaded exosomes suppress HN6 cell proliferation, migration, and invasion by downregulating SATB2. |
| (Li, Han et al. 2022) | patients requiring histopathological biopsy, including mucocele, OLK, and OSCC | N-MSCs (mucocele-derived MSCs), OLK-MSCs (OLK-derived MSCs), and Ca-MSCs (OSCC-derived MSCs) | cell culture supernatant | UC | TEM, NTA, WB, MS | CD9, CD63, CD81, Hsp70, TSG101, and calnexin | Exosomes from OLK-MSCs and Ca-MSCs exhibit strong pro-angiogenic effects by delivering high levels of MMP1, which enhances HUVEC migration and angiogenesis. |
| (Mayne, Woods et al. 2022) | 20 GORD  39 OPSCCs  19 Controls | NA | serum | ExoQuick™ kit | NTA commercial miRNeasy Serum/Plasma kit |  | A novel method, StaVarSel, identified a robust panel of serum sEV-derived miRNAs for detecting HPV+ OPSCC, offering a potential approach for developing diagnostic biomarkers for other head and neck cancers. |
| (Chen, Li et al. 2022) | 50 paired OSCC samples and normal adjacent | SCC-9, CAl27 Human immortalized HaCaT | clinical samples, cell culture supernatant, and animal | dUC | WB, (RT)PCR, | has_circ_0069313 2. PDL1 3.b-actin | has_circ_0069313 was upregulated in OSCC tissues and predicts poor prognosis |
| (Wang, Zhang et al. 2022) | 111 clinical samples tissues | SCC090, SCC47, CAl27, SAS and Human gingival fibroblast cell line HGF-1 | cell culture supernatant | UC | TEM, NTA, WB, Real time PCR | exosomal miR-9-5p, CD9, CD63, and TSG10, calnexin | HPV+ HNSCC-derived exosomal miR-9-5p inhibits TGF-β signalling-mediated fibroblast phenotypic transformation through NOX4, which is related to the excellent prognosis of HPV patients |
| (Smolarz, Skoczylas et al. 2022) | NA | FaDu, UM-SCC6 | cell culture supernatant | SEC | TEM, dynamic light scattering | CD9 and CD63 | Exosomes from irradiated FaDu cells can induce a bystander effect in recipient cells through replication stress, with early effects mediated by ligand–receptor interactions; about half of their characteristic proteins are membrane-associated. |
| (Bai, Hou et al. 2022) | NA | HN4 cells CNE2 | tissue and cell culture supernatant | Total Exosome Isolation a 0.22- mm PES membrane filter for diameter detection Reagent | WB, TEM, NTA | CD9 CD63 CD81 | exosome-specific markers (CD9, CD63, CD81) are upregulated, and the exocyst complex appears to regulate exosome secretion before Rab11a-mediated MVB–plasma membrane fusion; inhibiting exocyst proteins causes MVB accumulation, reduces exosome release, and may suppress cancer cell proliferation. |
| (Wang, Jiao et al. 2022) | NA | HN4, HUVEC cells | cell culture supernatant | Total Exosome Isolation Reagent | NTA, WB and qRT-PCR | GRP78 and ATF6 TSG101  and CD63 | miR-424-5p is the main component in  exosomes 2. the present study revealed  that LAMC1 was a direct target of miR-424-5p and the loss of  the LAMC1 repressed Wnt/β-catenin signalling pathway |
| (Wu, Liu et al. 2022) | OC patients  Free controls | NA | Serum samples | UC | Maldi-MS |  | Eight glycan structures were significantly elevated in OC-derived microvesicles, with greater increases in early-stage than advanced-stage disease; serum N-glycomic profiling also revealed significant alterations in 11 glycans, highlighting potential glycan-based biomarkers for early OC detection. |
| (Yamana, Yoshida et al. 2022) | 55 OSCC patients | SAS, and HSC-2 CRR cell line SAS-R | cell culture supernatant | UF, SEC | WB, TEM, NTA | CD9, CD63CD81 and ALIX | EVs contribute to radio resistance in OSCC via the miR-503-3p–BAK axis, identifying it as a potential therapeutic target, while circulating miR-503-3p may serve as a prognostic biomarker; however, SAS cell proliferation was unaffected by treatment with SAS or SAS-R EVs. |
| (Yuan, Wang et al. 2021) | 100 OSCC patients | HN4 | 100 OSCC patients | a total exosome isolation reagent | TEM, Nanosizer | CD63, CD81 TSG101 | ER-stressed OSCC cells release exosomes that polarize macrophages to the pro-tumour M2 phenotype and upregulate PD-L1, suggesting that ER stress enables OSCC cells to “educate” macrophages to support tumour growth. |
| (Xu, Liu et al. 2022) | All 20 4NQO-treated tongue samples were classified as follows: hyperplasia (n = 1), mild dysplasia (n = 2), moderate dysplasia (n = 6),  severe dysplasia (n = 6) and invasive squamous cell carcinoma (n = 5). | NA | blood | exoeasy maxi kit ultracentrifugation | WB, NTA, TEM, MS | CD63, CD9 TSG101, Calnexin | potential EV biomarkers and underlying pathological changes in early OSCC as well as the presence of oral-derived EVs in plasma and the need for pan-EV markers |
| (Yang, Ding et al. 2022) | 73 OSCC patients 113 healthy | NA | serum | UC | TEM, NTA |  | A saponin-based method enables rapid, high-throughput detection of intra-vesicular proteins directly from serum without EV isolation, effectively increasing detectable SCCA levels and improving its diagnostic accuracy for OSCC. |
| (Lou, Shi et al. 2022) | 65 patients  oral cancer | HN30, cal27 | cell culture supernatant | UC | TEM, NTA, WB, RT-PCR | CD81, CD9, NFIB | Exosomal miR-626 can facilitate the development of oral cancer by inhibiting the expression of its target NFIB. Exosomal miR-626 might be a therapeutic target for oral cancer |
| (Li, Qiu et al. 2022) | NA | UM-SCC083A and UPCI-SCC029B | cell culture supernatant | dUC | TEM, WB | miR-144/451a, s CD63, CD81,  and TSG101 | A novel biomimetic nanosystem (MEXO/CA) was developed to deliver the miR-144/451a cluster via exosome-like nanoparticles, increasing CD63, CD81, and TSG101 expression and more effectively inhibiting OSCC cell proliferation, migration, and invasion compared with free miR-144/451a. |
| (Shafiaa, Baghdadi et al. 2022) | NA | Laryngeal carcinoma cell line (HEp-2) | cell culture supernatant | UC | WB, TEM | CD31 and  CD81 | MSC-derived exosomes may be a viable supplementary anticancer therapy for  HNSCC by reducing these cells' invasiveness 2. MSC-derived exosomes have the potential to  be an additional anticancer therapy for  HNSCC by reducing the ability of these cells  to invade. |
| (Hong, Yu et al. 2022) | 5 patients with oral squamous cell carcinoma (OSCC) and 5 patients with oral ulcer 5 healthy samples | CAL27, Jurkat | saliva and cell culture supernatant | centrifuged at 120,000 g one-step simultaneous detected on the wedge-shaped and high magnetic field gradient mediated microfluidic chip. | WB, NTA | CD63, CD9 and CD81, CD45 and EpCAM could be chosen  as the biomarkers to distinguish oral cancer from oral ulcers | A magnetic material–based one-step detection platform was developed for simultaneous detection of oral ulcer– and oral cancer–derived exosomes, enabling early disease diagnosis with strong clinical application potential. |
| (Wang, Huang et al. 2022) | DMBA and control groups; each group had 3 animals | NA | saliva | UC | LC-MS | CD63 | found unique proteins present (desmocollin-2) or absent (Glucagon-cAMP-PKA-CREB pathway-related proteins) in the salivary exosomes of the pre-radiation DMBA-treated group (PreD 2. salivary exosomes carry proteins absent in tumour tissue and lackproteins that should be expressed in tumour tissue. |
| (Jiang, Zhou et al. 2022) | NA | s FaDu, CNE-2Z and Hep-2; the human esophageal epithelial cell line HEEC; human myeloid leukemia mononuclear cells THP-1; and HEK293T cells | cell culture supernatant | TER | EM, WB | CD9, CD63 | M1 macrophage–derived exosomes, via the lncRNA HOTTIP, suppress HNSCC progression by sponging miR-19a-3p and miR-19b-3p, thereby activating the TLR5/NF-κB pathway and promoting antitumor M1 macrophage polarization—highlighting a potential immunotherapy strategy. |
| (Benecke, Chiang et al. 2022) | 18 HNSCC Patients | NR | plasma EVs | galectin‑based  magnetic glycan recognition particles, EXÖBead® VS SEC | FCM, NTA, TEM, CRYO-EM | CD9, CD63, CD81 and TSG101 | Flow cytometry analysis showed that CD45⁻ PanEV⁺, EpCAM⁺ PanEV⁺, and PD-L1⁺ PanEV⁺ levels were significantly elevated in tumour patients compared to healthy controls. The EXÖBead® technique provided a reliable and functional method for rapid EV isolation. |
| (Liu, Zhang et al. 2022) | 25 four-week-old female BALB/C nude mice | SHED cell, (HUVECs) | cell culture supernatant | exosome concentration solution (ECS) kit | TEM, NTA, WB FACS | CD63 and TSG101 | Exosomes derived from stem cells of human deciduous exfoliated teeth (SHED-Exos) were efficiently taken up by endothelial cells and inhibited their proliferation, migration, and angiogenesis in vitro and in vivo by delivering miR-100-5p and miR-1246 to target VEGFA, resulting in a significant anti-tumour effect in OSCC. |
| (Liu, Li et al. 2021) | 3 healthy females | NA | tissue | UC | TEM, NTA, WB | CD63 | study suggest that ADSC‑Exos may represent a promising strategy for OSF  treatment by targeting the p38 MAPK signalling pathway |
| (Yang, Liu et al. 2021) | 3 patients,  20 female nude mice | SCC9 CAL27, BGC823 HCC-LM3 | cellular supernatant fluid, cell culture supernatant | UC | TEM, FCM, dynamic light scattering | IgG, CD9, CD63, and CD81 | A novel Au-EV nanomaterial was specifically taken up by SCC9 cells and combined with NIR irradiation, enhanced apoptosis. Let-7a-3p carried by EVs-Chrysin induced apoptosis, and Au-EVs with NIR significantly inhibited tumour growth in vivo. |
| (Wang, Zhu et al. 2021) | 31 HNSCC eight classical (CL), ten basal (BA), seven mesenchymal (MS), and six atypical (AT) cases 10 normal nasopharynx | HN4, NP69 and HN6 | tissue and cell culture supernatant | UF, total exosome isolation, UC | TEM, WB, NTA | CD9, CD63 TSG101 | Tumour-derived exosomes enriched in miR-185-3p promote proliferation, colony formation, tumour growth, and drug response in HNSCC by targeting RAB25, highlighting a potential therapeutic target. |
| (Cema, Dzudzilo et al. 2021) | 50 patients  20 patients’ healthy tissue | NA | saliva | OncAlert® |  | CD44 | Total protein level may serve as an indicator of early microenvironmental changes in leukoplakia but should be interpreted alongside SolCD44 testing for accurate assessment. |
| (Huang, Hsueh et al. 2021) | three pairs of HNSCC tissues and adjacent normal tissues | NA | plasma | UC | TEM, NTA IEM, Mass Spectrometry |  | HNSCC-derived sEVs carrying TGFβ1 activate normal fibroblasts into CAFs via the TGFβ1/fibronectin pathway, linking this mechanism to tumour clinicopathologic features and highlighting plasma sEV TGFβ1 as a potential diagnostic biomarker. |
| (Silva, Marti et al. 2021) | 15 OPHSCC patients  30 OSCC patients | SCC25, FaDu | plasma | UC, Total Exosome Isolation Reagent | TRPS, TEM, FCM | CD9, CD63 and CD81 | EVs from HNSCC cell lines impair monocyte-derived dendritic cell (mono-DC) maturation and function and carry miRNAs—such as miR-17-5p, miR-21, miR-16, miR-24, miR-181a, and a newly identified miR-23b—that mirror those found in HNSCC patient plasma, suggesting EV-mediated communication between tumours and immune cells. |
| (Yang, Luo et al. 2021) | NA | CAL 27 WSU-HN6 | Bitter melon derived extracellular vesicles | electrophoresis and dialysis-based methods | TEM, NTA WB | caspase 3 protein expression | PDEVs can enhance the therapeutic efficacy of chemotherapeutic agents and reduce cancer cell drug resistance, providing proof-of-concept for future PDEVs-based therapies. |
| (Goudsmit, Leprevost et al. 2021) | NA | UM-SCC-38, UM-SCC-47, UM-SCC-118, UM-SCC-104, and UPCI: SCC152 | serum-depleted, conditioned cell culture supernatant | PEG) precipitation/ultracentrifugation | TEM, NTA, Wes ProteinSimple Western, LC-MS, MS. | tenascin-C, HLA-A, E-cadherin, EGFR, EPHA2, and cytokeratin 19.cd9 | Extracellular vesicles (EVs) released by HNSCC cells carry selectively packaged proteins that do not fully mirror the originating cell’s protein profile but appear to support tumour-promoting processes such as metastasis, survival, immune evasion, microenvironment modification, and drug resistance. EV protein cargo varies based on characteristics of the source cell, including HPV status, and several proteins—tenascin-C, HLA-A, E-cadherin, EGFR, EPHA2, and cytokeratin 19—were identified as promising candidates for further evaluation as diagnostic or prognostic HNSCC biomarkers, highlighting their potential use in clinical liquid biopsy applications. |
| (Gluszko, Szczepanski et al. 2021) | NA | HNSCC cells (FaDu, PCI-30, SCC-25) and HaCaT keratinocytes | cell culture supernatant | SEC | CRYO-EM/WB, NTA, TEM immunoblotting, high- resolution MS | CD63 and CD9 | 15% of proteins detected in small extracellular vesicles (sEVs) were unique to hypoxic HNSCC cells, which shared a distinct signature of seven hypoxia-related proteins (KT33B, DYSF, STON2, MLX, LIPA3, NEK5, P12L1) and showed enrichment in pro-angiogenic proteins. The protein profiles of hypoxic sEVs reflected the degree of tumour hypoxia, suggesting their potential as sEV-based biomarkers for assessing hypoxic conditions. Adaptation of HNSCC cells to hypoxia was associated with increased sEV release carrying this unique protein profile, highlighting their value in evaluating tumour hypoxia. |
| (Liu, Li et al. 2021) | NA | SCC25 | cell culture supernatant | UC, UF | WB, a validated liquid chromatography tandem mass spectrometry assay | CD63, CD9 and CD81 | A gelatin biomimetic exosome co-carrier was successfully engineered to co-deliver SNS032 and TRAIL (MSCT-EXO/G-SNS032), achieving strong anti-OSCC activity in SCC25 cells by inducing apoptosis and concurrently modulating multiple pathways—downregulating JAK/STAT (JAK2/STAT3), suppressing anti-apoptotic proteins, and activating p53 and its downstream effectors. This synergistic nano-system shows high inhibitory efficacy and suggests a promising strategy for multidrug-resistant tumours, though findings are preliminary as they were demonstrated in a single cell line. |
| (Tang, Wan et al. 2021) | Ten p16INK4a (p16)-positive OPC patients  20 healthy controls (age and gender matched | NA | salivary exosomes | dUC | NTA, WB, TEM MASS SPEC | CD9, CD63 and CD81 | Salivary exosomes from HPV-driven oropharyngeal cancer (OPC) patients exhibited elevated levels of six key glycolytic enzymes (ALDOA, GAPDH, LDHA, LDHB, PGK1, and PKM), suggesting that these exosomes mediate a reciprocal interplay between glucose metabolism and HPV-driven OPC. The presence of HPV16 in salivary exosomes may further promote viral transmission and carcinogenesis, influencing exosomal protein content. Importantly, this study highlights the diagnostic potential of glycolytic enzymes in salivary exosomes for distinguishing HPV-driven OPC patients from healthy controls. |
| (Han, Zhang et al. 2021) | 10 patients’ intermediate stage of OSF | NA | tissue | different centrifugation and filtration | TEM, NTA, WB | CD9, CD63 and calnexin | Extracellular vesicles (EVs) carrying miR-375 inhibited the fibrosis of fibrotic buccal mucosal fibroblasts (fBMFs) by targeting FOXF1. Adipose-derived stem cell EVs (ADSC-EVs) suppressed OSF progression by inhibiting fBMF fibrosis through the miR-375/FOXF1 axis. |
| (Lu, Eguchi et al. 2021) | NA | HSC-3 and THP-1 | cell culture supernatant | combination method of size exclusion chromatography and concentration filters (SEC-CF) / PBP | TEM, WB | CD9, CD63, and two homologs of HSP90-α and β | The SEC-CF method effectively purifies both small and large exosomes (EXO-S and EXO-L) with high molecular transfer activity, enabling efficient delivery of molecular cargo to target cells. Notably, exosomes derived from macrophage-like cells using this method can reduce the viability of oral carcinoma cells, highlighting their potential therapeutic application. |
| (Yan, Wang et al. 2021) | NA | OECM1, HUVECs | serum and cell culture supernatant | ExoQuick ULTRA | TEM | Tsg101, Alix, and CD63. | Exosomal miR-130b-3p acts as a negative regulator of PTEN and promotes tubular formation in OSCC, indicating its potential role in angiogenesis. The study highlights the use of exosomes as molecular delivery tools to modulate tumour progression in vitro and in vivo, emphasizing the potential of exosome-based strategies for clinical applications. However, in vivo effects and validation in additional cell lines require further investigation. |
| (Ai, Wei et al. 2021) | NA | SCC-4 and CAL-27 | cell culture supernatant | UC | TEM, WB | CD63 and TSG101 | Exo-LBX1-AS1 secreted from RBPJ-overexpressing cells inhibits OSCC progression by regulating the LBX1-AS1/miR-182-5p/FOXO3 pathway. LBX1-AS1 suppresses OSCC cell proliferation and invasion, and RBPJ-OE-derived exosomes reduce tumour growth in vitro and in vivo. These findings suggest that LBX1-AS1 could serve as a diagnostic biomarker and a potential therapeutic target for OSCC. |
| (Zhou, Zhu et al. 2021) | 10 normal mucous samples  10 OSF samples  20 OSCC samples | CAL-27 and SCC9 | tissue cell culture supernatant | ExoQuick™ | TEM, NTA, WB | TSG101 and CD9 | Exosomal ADAMTS9-AS2 plays a crucial tumour-suppressive role in the cell microenvironment during OSF-to-OSCC progression. Downregulation of ADAMTS9-AS2 is associated with poor prognosis, while its presence in exosomes suggests it could serve as a promising biomarker for early detection and monitoring of OSCC. |
| (Zhu, Ji et al. 2021) | NA | SCC25 | cell culture supernatant | UC | SEM, desktop scanner | CD9 and CD63 | This study demonstrated, for the first time, that a desktop scanner can be used to quantitatively profile extracellular vesicle (EV) secretion from single cells without prior cell counting. The approach is low-cost, portable, dependable, and user-friendly, and it enables accurate quantification of EVs per cell using calibration curves generated from custom EV standards, providing a practical tool for single-cell EV analysis. |
| (Busso-Lopes, Carnielli et al. 2018) | NA | SCC-9 /LN1 | cell culture supernatant | dUC | NTA, uptake assay, high resolution microscopy (TEM, CRYO-EM | protein markers Annexin-2 and Flotillin-1 | Using an integrative multi-omics approach combining proteomics, miRNA profiling, metabolomics, and lipidomics, this study characterized EVs from primary tumours and paired metastatic oral cancer cell lines. The analysis identified a set of EV-associated molecules—including potential “hub proteins”—linked to metastasis, which may modulate signalling pathways in recipient cells. These EV cargo molecules could serve as prognostic markers for aggressiveness and lymph node metastasis in OSCC. |
| (Qu, Leung et al. 2021) | Ten non-cancer controls and fourteen patients with OTSCC | NA | plasma | SEC | TEM, NTA, WB | TSG101, CD9 | Comparative analysis of EVs from non-cancer controls and oral tongue squamous cell carcinoma (OTSCC) patients—with or without lymph node involvement—identified 43 unique EV-associated proteins with deregulated expression. Among these, actin, serum paraoxonase/arylesterase 1, integrin alpha-3, myosin-9, and agrin were highlighted as potential discriminatory biomarkers capable of distinguishing OTSCC patients based on nodal status or from non-cancer controls. |
| (Zhu, Cao et al. 2021) | Sixty-six patients with HNSCC | SCC-9 and CAL-27 | cell culture supernatant and serum | UC | SEM, WB | miR-192/215 | Hypoxia increases the levels of miR-192/215 in small extracellular vesicles (sEVs), which are internalized by stromal fibroblasts. These miR-192/215-rich sEVs downregulate CAV1 and activate TGF-β signalling, promoting the differentiation of fibroblasts into cancer-associated fibroblast (CAF)-like cells. This feedback mechanism supports tumour progression and remodelling of the hypoxic tumour microenvironment in HNSCC. Furthermore, sEVs derived from tumour tissues—rather than peripheral blood—may serve as potential carriers of biomarkers for HNSCC. |
| (Zhu, Wang et al. 2021) | One hundred and thirty patients with newly diagnosed HNSCC  mice (females, 6 weeks | SCC-9 and CAL-27 | Serum and cell culture supernatant | UC | SEM | LOXL2 | The hypoxic tumour microenvironment in HNSCC promotes local invasion of non-hypoxic tumour cells and stimulates the formation of pre-metastatic niches (PMNs). These effects are mediated by HNSCC-derived small extracellular vesicles (sEVs), which deliver LOXL2 to non-hypoxic HNSCC cells and fibroblasts, inducing epithelial-mesenchymal transition (EMT) in tumour cells and fibronectin (FN) production in fibroblasts, thereby facilitating tumour progression and metastatic niche preparation. |
| (Bottino, Rodrigues-Junior et al. 2021) | NA | HNSCC (NCC–HN19) and the HEK293T cell lines | cell culture supernatant | the supernatant was filtered on a 0.22 μm filter and further concentrated 20X by tangential flow filtration on a 50 kDa Ultra-15 Centrifugal Filter (Millipore) by centrifugation at 1200×g. | NTA | CD9 and ALIX | HNSCC-derived vesicle-free supernatant (VSF) modulates macrophage inflammatory responses by reducing IL-1β and caspase-1 secretion. This effect is associated with the enrichment of TGF-β–modulatory factors in the VSF, which specifically impacts the priming phase of NLRP3 inflammasome activation, highlighting a mechanism by which HNSCC cells can suppress macrophage-mediated inflammation. |
| (Gong, Donnelly et al. 2021) | 195 HNSCC patients | MOC2-E6/E7 and NOOC1 | cell culture supernatant | Total Exosome Isolation | FCM, NTA | cd63, PDL-1 Galectinn | Elevated MX1 protein expression in HNSCC tumour cells is associated with poor patient prognosis, suggesting its potential as a prognostic biomarker. |
| (Cohen, Betzer et al. 2021) | NA | MSC-exo, and exosomes from the A431 human epidermal carcinoma cell line (A431-exo) | cell culture supernatant | UC | NTA, WB, HR-SEM. | CD63 | GNP labelling combined with in vivo CT tracking of exosomes indicated differences in tumour targeting.  between several types of exosomes. Moreover, the labelling of exosomes allowed for quantification of intra-tumour exosome accumulation and biodistribution of the different exosome types |
| (Pang, Wang et al. 2021) | 5 normal oral epithelial tissues and 45 OSCC | Cal-27 and SCC25 / human  monocytes cell line THP-1 | cell culture supernatant | UC | qPCR, Western Blot | PDL-1, calnexin, CD63, α-tublin--CD9, CD63,CD81, CMTM6 | OSCC cells secrete exosomal CMTM6, which induces M2-like macrophage polarization via the ERK1/2 signalling pathway, promoting tumour progression.  CMTM6 expression correlates with CD163⁺ macrophage infiltration and adverse clinical features in OSCC patients.  Targeting CMTM6, potentially in combination with PD-L1 blockade or immunotherapy, could represent a novel therapeutic strategy for OSCC. |
| (Nakamichi, Sakakura et al. 2021) | serum samples:  29 OSCC  21 HC  saliva samples:  23 OSCC  20 HC | NA | Serum and saliva | UC | WB, TEM, and ELISA | Alix, CD9, CD63, CD81 | 1. Serum/salivary exoAlix levels were significantly higher in patients with OSCC than in HC 2.: Serum and salivary exoAlix were identified as potential diagnostic OSCC  biomarkers. Serum exoAlix was suitable for prediction of therapeutic responses |
| (Li, Shi et al. 2021) | 40 OSCC | OSCC cell lines (SCC-4, HSC-3, TSCC1, SCC090,  and HN-4) and normal human oral keratinocyte  cell line (NHOK) | cell culture supernatant | ExoQuick precipitation | TEM, RT-qPCR and  WB | CD9, CD63 | Exosomes transfer APCDD1L-AS1, which promotes 5-fluorouracil (5-FU) resistance in OSCC cells.  The mechanism involves the miR-1224-5p/NSD2 axis, where APCDD1L-AS1 sponges miR-1224-5p, leading to upregulation of NSD2.  Targeting exosomal APCDD1L-AS1 or its downstream axis may provide a novel therapeutic approach to overcome chemoresistance in OSCC. |
| (Guo, Mao et al. 2021) | NA | SCC-9, SCC-15, and FaDu, HepG2 | cell culture supernatant | UC | WB, NB Qrt-PCR | HSP70, CD63, and TSG101//ANLN, | High expression of ANLN in HNSCC patients is associated with poor prognosis. The study demonstrated that ANLN-210 is packaged into exosomes and delivered to macrophages via the RNA-binding protein hnRNPC. Once transferred, exosomal ANLN-210 promotes macrophage polarization through the PTEN/PI3K/Akt signalling pathway, which in turn stimulates tumour growth, highlighting a novel mechanism by which tumour -derived exosomes modulate the immune microenvironment to promote HNSCC progression. |
| (Guo, Jiang et al. 2021) | 10 HD  20 OSCC | NA | serum | UC | TEM, FCM nano analyzer | CD9, CD63, and HSP70 | CRP, VWF, and LRG as potential clinically relevant biomarkers for OSCC. It also suggested that OSCC-derived small extracellular vesicles (sEVs) may promote the migration of oral cells. Notably, combinations of these biomarkers demonstrated higher diagnostic or predictive value compared to individual markers, indicating that multi-marker panels could improve clinical assessment in OSCC. |
| (He, Shao et al. 2021) | NA | CAL 27 | cell culture supernatant | UC | TEM | CD63 | A novel electrochemical method was established for the detection of salivary exosomes. The system combined engineered red blood cell membranes (RBCMs) to provide antifouling and targeting capabilities with silver nanoparticles (AgNPs) to generate strong electrochemical signals. This dual design enabled sensitive and precise detection, achieving a detection limit of 2.07 × 10² particles/mL, outperforming existing methods. Additionally, the approach demonstrated excellent reproducibility and effectively detected target exosomes in saliva samples, highlighting its potential for clinical applications. |
| (He, Zhang et al. 2021) | NA | HUVECs, Cal27 | OSCC tissue or peripheral blood serum | ExoQuick, UC | qRT-PCR, WB, TEM | Tsg101, Alix, and CD63. | The study demonstrated that PIK3R1 expression is decreased while miR-221 expression is increased in OSCC tissues compared to normal controls. Functional analyses suggested that OSCC-derived exosomal miR-221 promotes HUVEC migration and angiogenesis by targeting PIK3R1. These findings indicate that both miR-221 and PIK3R1 may serve as potential therapeutic targets for the clinical treatment of OSCC. |
| (He, Guo et al. 2021) | 184 OSCC  196 HD | NA | Plasma | ExoQuick | qRT-PCR | exosomal miR-130a | provided the first evidence that  plasma-derived exosomal miR-130a expression was significantly increased in OSCC patients and associated with late T-stage, advanced TNM stage, and poorly differentiated grade. Moreover, exosomal miR-130a may serve as a promising diagnostic and prognostic biomarker for OSCC  patients. |
| (Lee, Wu et al. 2020) | NA | SAS and HSC-3 | cell culture supernatant | total exosome isolation kit | WB, ELISA | Alix, c-Met, STAT3, CD44, and PD-L1. | Tumorsphere-Derived Exosomes (Exosp) Promoted c-Met/STAT3/CD44/PD-L1 Expressions, Cisplatin Resistance, and Tumour-Initiating Ability.HNC0014 also normalized the tumour microenvironment by reducing CAF transformation, in part,  by reducing tumorsphere-derived exosomes cargoes. |
| (Zheng, Song et al. 2020) | 108 OSCC  50 Healthy | HN6, CAL27 | cell culture supernatant | UC | TEM, qRT-PCR and Western blot analysis, | TSG101, CD9, CD63,  CANX | present study described the roles of EVs-transmitted miRNAs on erlotinib resistance. Targeting the dysregulated immune system could be the effective method to overcome erlotinib-resistance in HNSCC cells. Erlotinib inhibited canonical EGFR signalling and increased proliferation in resistant cells |
| (Guerreiro, Ovstebo et al. 2020) | NA | E10 (OSCC), pancreatic ductal adenocarcinoma, human melanoma brain metastasis | cell culture supernatant | SEC | NTA, TEM, WB | E10 and BxPC3 were positive for CD9 marker, while its expression was extremely low in the vesicles from H3. The EVs were examined for the detection of the markers CD9, CD81, and CD63 by WB. Here only CD9 was detected. | Proteomic analysis of the vesicles isolated from cell culture supernatant from the three cell lines identified 678 different proteins from the E10 derived Evs. 25% of the proteins were common to three cell lines. the panels of proteins associated with cell proliferation, migration, and adhesion were extensive for both E10 and BxPC3 |
| (Luo, Liu et al. 2020) | 108 patients OSCC 50 healthy | OSCC cell lines (SCC4, SCC9, SCC25, HN12, CAL27) | exosomes from serum | ExoEasy Maxi Kit | TEM, qPCR | cd63, TSG101 | patients with high exosomal circ_0000199 had higher tumour recurrence rate and higher mortality rate than the patients with low exosomal circ_0000199 |
| (Wang, Wang et al. 2020) | 80 OSCC tumours  7 normal mucosa tissues | CAL27, HUVECs | cell culture supernatant from cal27 | UC | WB, RTPCR, TEM, DLS | Tsg101, CD63, Hsp90 | miR-210-3p is significantly overexpressed in OSCC tissues compared to normal oral mucosa and has been reported as a potential diagnostic biomarker in other diseases, including Graves’ disease and colorectal cancer. Tumour-derived exosomes serve as key mediators in the tumour microenvironment, transferring miR-210-3p from OSCC cells (e.g., CAL27) to recipient cells such as HUVECs. This transfer upregulates miR-210-3p in HUVECs, promoting angiogenesis by targeting ephrinA3 and potentially activating the PI3K/AKT signalling pathway, thereby enhancing endothelial cell proliferation and migration. These findings highlight the pivotal role of exosomal miR-210-3p in OSCC angiogenesis and intercellular communication within the tumour microenvironment. |
| (Beccard, Hofmann et al. 2020) | 41 HNSCC patients | NA | plasma | size exclusion chromatography | TEM, WB, NTA | TSG101, CD45, EpCAM | Exosomes derived from the plasma of high UICC stage HNSCC patients exhibit stronger immunosuppressive effects compared to those from low-stage patients. Notably, CD45⁻ and CD45⁺ exosomes have different functional impacts: CD45⁻ tumour-derived exosomes (TEX-enriched) primarily reflect tumour aggressiveness, making them a promising liquid biomarker for cancer progression, while CD45⁺ hematopoietic exosomes serve as indicators of immune suppression within the tumour microenvironment. This distinction provides new insights into TEX- and non-TEX-mediated modulation of the TME and potential biomarker applications in HNSCC. |
| (Jin, Jin et al. 2020) | 145 patients with OSCC | TCA8113 cell line | tissue cell culture supernatant | ExoQuick-TC | qRT-PCR, WB | snail., ZEB1 GARPDH, (a-SMA and b-catenin) | The study reveals a novel molecular mechanism by which TIRY in cancer-associated fibroblasts (CAFs) modulates OSCC progression and identifies TIRY as a potential therapeutic target. OSCC tissues showed increased TIRY expression and decreased miR-14 levels, with higher TIRY correlating with poor prognosis. Mechanistically, TIRY overexpression in CAFs reduced the levels of miR-14 and Snail in CAF-derived exosomes, thereby limiting miR-14 delivery to epithelial cancer cells and promoting tumour invasion and metastasis. Conversely, TIRY knockdown enhanced miR-14 transfer via exosomes, significantly reducing OSCC invasion and metastasis. |
| (Higaki, Shintani et al. 2020) | NA | A431 cell | cell culture supernatant | MagCapture™ Exosome Isolation Kit PS | SEM, WB | presence of CD9 and absence of cytochrome c | ED-71 stimulates exosomal miR-6887-5p in  SCC/OSCC cells, and that miR-6887-5p suppresses tumour growth in vitro and in vivo as well as colony formation of SCC/OSCC cells via direct targeting of HBp17/FGFBP-1. |
| (Amit, Takahashi et al. 2020) | OSCC induced mouse model, adjacent normal tissue, no tumour mouse model | NA | tissue | dUC | NTA, TEM, WB | CD63, microRNA | Cancer derived EV control neurogenesis. Cancer Evs drive sensory nerve reprogramming |
| (Tong, Mao et al. 2020) | 47 HNSCC samples/ 516 cases (303 cases were radiotherapy-treated, 223 and 34 were defined as complete response and no response) | SCC90, SCC47, SCC104, SAS, CAL33, CAL27 | cell culture supernatant and tissue | UC | TEM, NTA, WB RT-PCR | CD9, CD63, TSG101, Calnexin | HPV-positive HNSCC miR-9–enriched exosomes, are significantly higher in CD9 levels compared to HPV-negative HNSCC.  These miR-9–rich exosomes polarize macrophages toward the M1 phenotype by downregulating PPARδ.  M1 polarization enhances radiosensitivity of HPV-positive HNSCC cells.  Overall, exosomal miR-9 is identified as a key mediator linking HPV infection to improved HNSCC response to radiation therapy. |
| (He, Ping et al. 2020) | 45 preoperative OSCC patients compared to 10 normal controls. 30 tissue samples | HSC6 | saliva | ExoQuick-TCTM | TEM, NTA, RT-PCR | CD63, CD81 TSG101 GADPH | Salivary exosomal miR-24-3p is a potential novel diagnostic biomarker for OSCC, and miR-24-3p can maintain the proliferation of OSCC cells through targeting PER1 |
| (Abramowicz, Łabaj et al. 2020) | NA | FaDu | cell culture supernatant | dUC UF and precipitation | TEM, DLSA, WB | CD63, CD81, TSG101, ALIX, GRP94, PHB1 | exosomes and their miRNA cargo could represent a link between radiation-activated DDR and inflammation-related processes like SASP. |
| (Ono, Sogawa et al. 2020) | NA | HSC-3, HSC-3-M3, Ca9-22, HO-1-u-1, SAS, HSC-2, HSC-4, and THP-1 | cell culture supernatant | Total Exosome Isolation | WB, TEM | CD63, CD9, HSP90α, HSP90β, MMP9 | Metastatic oral cancer cells secrete HSP90-high EVs, which transfer to recipient cells. These EVs trigger epithelial–mesenchymal transition (EMT) in normal epithelial cells, and promote migration, invasion, tumorigenesis, and M2-like macrophage polarization.  Triple siRNA silencing of CDC37/HSP90α/HSP90β reduces EMT marker expression in oral cancer cells.  HSP90-depleted EVs show diminished transmission ability and reduced pro-malignant effects in recipient cells. |
| (Kulkarni, Gondaliya et al. 2020) | Volunteers Healthy 7 Volunteers (with tobacco history)  7 OSCC patients  6 OSCC patients with tumour recurrence (post cisplatin treatment) | SCC084 | Serum, cell culture supernatant | UC | WB, Qpcr, RT.PCR | CD9, β-Actin | In cisplatin-resistant (cisRes) OSCC cells and patients, exosomal miR-30a levels are downregulated, while Beclin1 expression is upregulated. |
| (Qiu, Chen et al. 2020) | NA | SCC7, SCC25, Cal27 and C2C12 | cell culture supernatant | UC | DLS, TEM, WB | CD81, TSG101, calnexin | Tunicamycin-treated OSCC conditioned media induces C2C12 myotube atrophy and apoptosis via TERS signalling.  OSCC-derived exosomes mediate this TERS signalling, promoting muscle cell atrophy and apoptosis.  Exosomal miR-181a-3p is identified as a potential key regulator of this process. |
| (Li, Liu et al. 2020) | 4 OSCC patients | primary CAFs | cell culture supernatant | UC then Total Exosome Isolation Kit | BCA protein kit, WB | CD63, CD81, CD9, heat shock protein 70 AND calnexin | CAF-secreted VEGF binds to HSPG on the surface of small EVs (sEVs), potentially contributing to bevacizumab resistance in OSCC.  Heparinase treatment releases VEGF from sEVs, and cells exposed to this dissociated VEGF become sensitive to bevacizumab.  Combining heparinase with bevacizumab may enhance anti-angiogenic therapy in patients with important levels of sEV-bound VEGF. |
| (Zhang, Tang et al. 2020) | 30 OSCC patients | CAL-27, SCC-15, and HOK | cell culture supernatant | ExoQuick | TEM, (qRT-PCR), WB | CD63 and CD9 | Exosomal circGDI2 regulates malignant behaviours of OSCC cells.  It acts through the miR-424-5p/SCAI axis.  circGDI2 is highlighted as a potential novel exosome-based biomarker and therapeutic target for OSCC. |
| (Shoff, Booker et al. 2020) | NA | SCC4, SCC15, SCC25 and CAL27 | cell culture supernatant | Total Exosome Isolation reagent | NTA | miR-21 and miR-34. | Exosomes from OSCC stem cell populations show consistent downregulation or loss of miR-21 and miR-34.  This suggests a unique miRNA profile characteristic of oral cancer stem cells. These results emphasize the need to investigate the roles of miR-21 and miR-34 in tumour progression. Developing a refined miRNA profile could help identify tumours with poor prognosis. |
| (Qiu, Sun et al. 2020) | NA | SCC25 | cell culture supernatant | UC | TEM, WB, MASS SPEC | CD63, CD9 and TRAIL | MSCT-derived exosomes (MSCT-EXO) can be used as a CTX delivery system, representing a potential anticancer strategy.  CTX-loaded MSCT-EXO induces apoptosis in OSCC cells, partly by inhibiting the PI3K/Akt/mTOR signaling pathway.  MSCT-EXO enhances the apoptotic effect of CTX specifically in SCC25 cells. |
| (Ludwig, Sharma et al. 2020) | NA | HPV (+) cell lines (UD-SCC-2, UM-SCC47, UPCI: SCC90), HPV (−) cell lines (PCI-13 and PCI 30) | cell culture supernatant | SEC | qRT-PCR , nano string analysis | genomic | genomic analysis of exosomes produced by HNC cell lines indicates that exosomes can discriminate the HPV (+) from HPV (−) cells |
| (Sanada, Islam et al. 2020) | 36 serum samples from HNSCC patients  seven healthy volunteers | NA | Serum samples | UC, Total Exosome Isolation | ELISA, Immunolabeling | LOXL2 | Serum exosomal LOXL2 levels are elevated in HNSCC patients.  Higher exosomal LOXL2 levels are associated with low-grade HNSCC, suggesting potential as a diagnostic or prognostic biomarker. |
| (Ludwig, Yerneni et al. 2020) | 12 patients with HNSCC  5 normal donors | UMSCC47 | cell culture supernatant and tissue | mini-SEC | TEM, TRSPS, WB UPLC, MS/MS, FCM, HPLC | adenosine | Tumour-derived exosomes (TEX) carry a complex cargo, including adenosine (ADO), ADO metabolites, and active ectonucleotides, which promote angiogenesis. A2BR-mediated signalling is identified as a novel pathway for exosome-induced angiogenesis. TEX reprogram endothelial cells (ECs) to an angiogenic phenotype both by direct interaction and indirectly by modulating other TME cells, such as macrophages. |
| (Jin, Huang et al. 2020) | 10 patient’s Normal oral mucosa | OSCC cell line CAL-27 and human oral epithelial cells (HOECs) | cell culture supernatant | ultracentrifugation | WB, TEMP and Particle Size Analyzers | ALIX, TSG101 | Three lncRNAs—NR-026892.1, NR-126435.1, and NR-036586.1—were identified as potential diagnostic biomarkers for OSCC.  These lncRNAs were significantly differentially expressed in CAL 27-derived exosomes compared to HOEC-derived exosomes, as well as in whole CAL 27 cells versus HOECs. |
| (Zhu, Qin et al. 2020) | NA | OC cell lines (WSU‑HN4 and SCC‑9) | cell culture supernatant | UF, affinity  chromatography ExoEasy Maxi kit | TEM, NTA, WB, MS | CD63, Rab5, CD9, Alix | OC cell-derived exosomes (OCEXs) are internalized by NK cells, altering NK cell surface receptor expression.  Short-term exposure to OCEXs enhances NK cell function, while long-term exposure leads to reduced NK cell cytotoxicity |
| (Tomita, Sasabe et al. 2020) | NA | OSC-4 cells, THP-1 | cell culture supernatant | Total Exosome Isolation kit | (BCA) assay | CD9, CD63 Rab-5B | Macrophage-derived exosomes are taken up by OSCC cells and can promote chemoresistance. This effect occurs through activation of the AKT/GSK-3β signaling pathway.  Consequently, OSCC cells become less sensitive to chemotherapeutic agents. |
| (Razzo, Ludwig et al. 2020) | NA | Murine HNSCC cell line SCCVII, human HPV (+) HNSCC line SCC90 and HPV (−) HNSCC line PCI-13 | cell culture supernatant | UF, UC | TEM, TRPS, WB | FasL, PD-L1, CD39, CD73 and a truncated form of COX-2 | The 4NQO-induced OSCC model demonstrates that tumour -derived exosomes (TEX) from both murine and human sources contribute to chemical carcinogenesis.  TEX promote cell transformation, tumour progression, and reprogramming of the host immune system, reducing its protective effects. |
| (Wang, Li et al. 2020) | 10 HPV-OPC patients | NA | saliva | acoustofluidic (the fusion of acoustics and microfluidics) | WB, TEM, RT-PCR | CD63, TSG101, and HSP90 | HPV16 can be successfully detected from salivary exosomes using an acoustofluidic platform.  This method offers advantages for salivary exosome-based liquid biopsy, including early detection, risk assessment, and screening for HPV-associated oropharyngeal cancer (HPV-OPC). The approach provides a non-invasive, sensitive, and rapid platform for early detection, risk assessment, and screening of HPV-OPC. |
| (Sato, Vasaikar et al. 2019) | NA | HUVECs, HMVECs and OSC19, Detroit 562, and SCC61 | cell culture supernatant | UC | NTA, BCA, TEM, WB | CD31 | HNSCC-derived EVs carry PHB2, which can induce ephrin-B reverse signalling. This mechanism promotes tumour angiogenesis. EVs uniquely facilitate angiogenesis by transporting Eph transmembrane receptors to nonadjacent endothelial cells, triggering ephrin reverse signalling. |
| (Li, Zhou et al. 2019) | 30 OSCC | NA | serum | SBI system Smart SEC | TEM, nano analyser, WB | CD9, CD63, and HSP70 | ApoA1, CXCL7, PF4V1, and F13A1 are identified as potential predictive circulating biomarkers for OSCC with lymph node metastasis (LNM).  These biomarkers are not useful for prognosis.  Combined use of these biomarkers is recommended to reduce clinical misdiagnosis of OSCC-LNM. |
| (Xie, Du et al. 2019) | 36 oral cancers | HEK-293T cell, human oral cancer cell lines (CAL27, TCA8113, SCC9, SCC25, HN4), and  normal oral cell line (NOK) | cell culture supernatant | UC | WB, RTqPCR | CD9, CD63 | miR-101-3p inhibits oral cancer development and progression by targeting COL10A1.  Human bone marrow-derived MSCs (hBMSCs) transfer miR-101-3p to TCA8113 cells via exosomes, suppressing their proliferation, invasion, and migration. |
| (Li, Han et al. 2019) | NA | DOK cell line, SCC15 cell line | cell culture supernatant | dUC | TEM, BCA, Confocal, WB | CD63 and CD9 | MSCs interact with both dysplastic oral keratinocytes and oral tumour cells, influencing oral carcinogenesis. MSC-derived exosomes contain miR-8485, which functions as an oncogene in oral cancer development. Highlights the importance of MSCs from premalignant lesions and the exosomes they secrete in promoting tumour progression. |
| (Wang, Qin et al. 2019) | 115 HNC tissues 102 normal oral epithelial tissues; 64 pairs of HNC tissues and adjacent normal tissues; 86 plasma samples from HNC patients and 36 plasma samples from healthy individuals; and 35 pairs of post-operation and pre-operation plasma samples from HNC patients | HN4, HN30 CAL 27, 293 T and MC-3 T3-E1 | cell culture supernatant | UC, UF | TEM, NTA, RTPCR | Alix, HSP90, CD63, CD9 and CD81 | CAF-derived exosomes have lower levels of miR-3188 compared to normal fibroblasts (NFs).  miR-3188 acts as a signal mediator between CAFs and tumour cells. Loss of exosomal miR-3188 promotes malignant phenotypes of HNC cells by derepressing BCL2. In vivo studies suggest that exosomal miR-3188 has therapeutic potential for inhibiting HNC growth. |
| (Zlotogorski-Hurvitz, Dekel et al. 2019) | 21 OC patients  13 HI | NA | blood | dUF | TEM. NTA, WB, FTIR | CD9, CD81 and CD63 | Oral cancer (OC) salivary exosomes exhibit a specific IR spectral signature.  These exosomes can be accurately distinguished from healthy individual (HI) exosomes.  Differentiation is achieved by detecting subtle conformational changes in proteins, lipids, and nucleic acids using optimized artificial neural networks, even with small data sets. |
| (Rodrigues-Junior, Tan et al. 2019) | 38 HNSCC patients | NA | FaDuATCC HTB-43 and SCC25-ATCC CRL-1628) or patient-derived (NCC-HN120 and NCC-HN137) | tangential flow filtration on a 50 kDa Ultra-15 UF | ZetaView, NanoSight, SEM, ELISA, WB | CD81, CD9 | TGFβ3, transmitted via EVs, plays a key role in HNSCC resistance to cytotoxic therapy.  Elevated levels of TGFβ3 in plasma EVs serve as an independent predictor of disease progression in patients with locally advanced HNSCC undergoing chemoradiotherapy. |
| (Ji, Qi et al. 2019) | NA | SCC25, UM-SCC6. | cell culture supernatant | Multipexed | AFM | CD81 and CD63, CD9, HSP70 and EpCAM | Decreased levels of certain EV phenotypes (e.g., CD63+ EVs) are associated with invasiveness in OSCC cell lines and primary OSCC cells. The study established multiplexed detection of EV and cytokine secretion from the same single cells, allowing analysis of cellular communication in a multidimensional manner.  Tiered functional subgroups with distinct secretion profiles were identified using clustering and principal component analysis, showing that different subgroups dominate EV or cytokine secretion. This technology enables comprehensive single-cell evaluation of EV secretion heterogeneity, complementing current single-cell and EV research. |
| (Ramayanti, Verkuijlen et al. 2019) | 33 Healthy  39 NPC  29 non-NPC malignancies | EBV positive NPC cell line C666-1 and EBV negative cell lines NPC-derived HONE1 | serum | SEC | WB, TEM | BART13-3p | Circulating EV-bound EBV-BART13-3p shows high specificity (97%) for nasopharyngeal carcinoma (NPC) detection.  This reflects active secretion from NPC tumour cells.  EV-bound BART13-3p is a promising NPC-selective biomarker suitable for screening strategies, especially in endemic regions. |
| (Rodrigues-Junior, Tan et al. 2019) | locally advanced HNSCC who responded (CR, n = 6) or presented incomplete response (NR, n = 6) to CRT | NA | plasma | using beads coated with CTB | Antibody Array | Proteins | Biomarker candidates were identified in plasma EVs and crude plasma.  These biomarkers can stratify HNSCC patients based on their response to chemoradiotherapy (CRT). |
| (Qin, Guo et al. 2019) | 80 pairs of tumour and adjacent normal tissues  40 HNC plasma and 30 physicals | SCC-4, SCC-9, SCC-25, CAL 27, and 293T cells, HN4res | Plasm, cell culture supernatant | dUC for CM, exoquick for plasma | NTA, TEM, BCA Protein | exosome or exosomal miR-196a depletion from CAFs could functionally restore the cisplatin response in HNC cells | Exosomes from CAFs or cisplatin-treated CAFs promote growth and survival of recipient HNC cells.  miR-196a is functionally transferred from CAFs to HNC cells via exosomes, mediating these effects. |
| (Dourado, Korvala et al. 2019) | NA | HSC-3, SASSCC-15 and SCC-25/Five primary cell cultures of both CAF and normal fibroblasts (NOF) were established, respectively, from OSCC and healthy human oral mucosa. | cell culture supernatant | dUC | qRT-PCR, WB, NTA and ImmunoEM | CD81, TSG101, FLOT1, and ALIX | CAF-derived EVs (CAF-EVs) promote migration and a budding pattern of invasion in OSCC cells.  CAF-EV-treated tumour cells show altered gene expression, including upregulation of invasion-related genes such as ROCK2, FLOT1, and FAM129B, and changes in metabolic pathways.  Demonstrates active transfer of EV cargo from the tumour stroma to tumour cells, elucidating molecular mechanisms driving OSCC progression. |
| (Jiang, Xu et al. 2019) | 3 patients with OSCC | CAL27 | cell culture supernatant | dUC | SEM, TEM, DLS, WB, FCM | GAPDH, FAB | Microvesicles (MVs) from tumour cells regulate glucometabolic reprogramming in surrounding stromal cells, creating a tumour-supportive microenvironment.  This illustrates a mechanism of tumour progression via tumour–stromal crosstalk through the reverse Warburg effect mediated by TMVs. These findings highlight potential targets for OSCC prevention and treatment. |
| (Li, Lu et al. 2019) | NA | Human gd T cells were expanded and cultured from human PBMCs | cell culture supernatant | ExoQuick EV | WB, FCM, qRT-PCR | CD63, tubulin | gdTDEs delivering miR-138 exert synergistic therapeutic effects on OSCC.  The benefits arise from direct anti-tumour effects on OSCC cells and immunostimulatory effects on T cells.  gdTDEs can function as an efficient drug delivery system (DDS) for microRNAs in cancer treatment. |
| (Zhong, Ren et al. 2019) | 65 patients with OSCC, 21 patients with oral ulcer (OU), and 42 healthy donors were purified | NA | Saliva | dUC | TEM, DLS, CFSE and FCM |  | level of SMVs is elevated in patients with OSCC, and the increased level of SMVs is closely correlated with the clinical data and outcomes of OSCC patients. |
| (Li, Cao et al. 2019) | NA | cell lines SCC-9, SCC-4, and CAL-27/SCC cell line SCC-VII | cell culture supernatant | ExoQuick | WB | CD81, CD63 and tubulin | OSCC tumour-derived exosomes (TEXs) stimulate γδ T-cell expansion and function. Hypoxia attenuates this effect by enhancing MDSC-mediated suppression via the miR-21/PTEN/PD-L1 axis. Therapeutic targeting of miR-21 and PD-L1 improves outcomes in OSCC-bearing immune-competent mice.  Tumour oxygen levels regulate the balance between anti- and pro-tumoral γδ T cells by altering TEX content, which in turn modulates MDSC function through the miR-21/PTEN/PD-L1 axis. Suggests a potential combined exosome inhibition and immune checkpoint therapy for OSCC. |
| (Wang, Chen et al. 2019) | NA | FaDu | cell culture supernatant | PEG-mediated precipitation | TEM, WB, BCA protein | CD63, GAPDH | Exosomes from 293 cells can encapsulate TRPP2 siRNA in a concentration-dependent manner and remain stable in serum and against nucleases.  FaDu cells efficiently take up the exosome/TRPP2 siRNA complexes.  Treatment with these complexes suppresses TRPP2 expression, inhibits EMT, and reduces migration and invasion in FaDu cells.  Suggests the potential of exosome/TRPP2 siRNA complexes as RNA-based gene therapy for head and neck cancer (HNC). |
| (Wang, Liou et al. 2019) | NA | LN1-1, OEC-M1, HUVECs | cell culture supernatant | dUC | EM, DLS, WB | CD9, CD63 and CD81 | LN1-1 cell-derived EVs (LN1-1 EVs) promote lymphatic endothelial cell (LEC) migration, tube formation, and uptake more effectively than OEC-M1 EVs.  Laminin-332 proteins (α3, β3, γ2) are highly expressed in LN1-1 EVs.  Clinically, higher levels of EV-borne laminin-332 are found in plasma from OSCC patients with lymph node metastasis compared to healthy controls and OSCC patients without metastasis.  Suggests EV-laminin-332 as a novel noninvasive biomarker for detecting lymph node metastasis in OSCC |
| (Wang, Yin et al. 2019) | NA | Murine bone marrow-derived MSCs ( | cell culture supernatant | GETTM Exosome Isolation Kit (for stem cells) | NTA, EB, qPCR, TEM | CD9, CD81 and flotillin-1 | Locally delivered EVs carrying miR-185 were assessed as a therapeutic strategy for oral potentially malignant disorders (OPMD) in a hamster model.  Genetically modified MSC-derived EVs were able to modulate inflammation, inhibit cell proliferation, and promote apoptosis, demonstrating potential efficacy for OPMD treatment. |
| (Cai, Qiao et al. 2019) | Twenty-four specimens of OSCC tissue and that of paired adjacent tissues | SCC-9 and CAL-27/ normal human oral epithelial cell (HIOEC) line | cell culture supernatant | ExoQuick precipitation solution | qRT-PCR, WB, FCM | Exosome-enclosed miR-29a-3p promoted polarization of macrophages to the M2 subtype, which subsequently enhanced the ability of proliferation and invasion of OSCC | miR-23a-3p is enriched in OSCC-derived exosomes.  These exosomes are taken up by unpolarized macrophages, driving their polarization toward the pro-tumour M2 subtype via SOCS1/STAT6 signaling.  OSCC cells use exosome-enclosed miR-23a-3p to modify the tumour microenvironment, promoting tumour cell proliferation and invasion.  Provides a theoretical basis for targeting OSCC exosomal miR-23a-3p in therapy. |
| (Sun, Xu et al. 2019) | Forty‑seven OSCC patients | CAL27 | cell culture supernatant | a Hieff™ Quick Exosome Isolation Kit | WB, Transfection | miR-382-5p | CAF-derived exosomes transport miR-382-5p to OSCC cells. This transfer promotes OSCC cell migration and invasion, revealing a mechanism of CAF-induced OSCC progression. |
| (Ono, Eguchi et al. 2018) | NA | HSC-3 OSCC, metastatic subline HSC-3-M330 | cell culture supernatant | polymer-based precipitation VS UC | TEM, Nno analyzer, WB, mass spec | CD9, EpCAM | Metastatic oral cancer cells secrete extracellular vesicles (EVs) enriched with molecular chaperones, including HSP90α and HSP90β.  HSP90, TRAP1, and HSP105 in EVs are identified as potential biomarkers for the metastatic phenotype and as prognostic biomarkers in head and neck cancers (HNCs). |
| (Chen, Feng et al. 2018) | NA | J1.1 and 8E5/LAV, 2D10 and C22G cells | cell culture supernatant and human plasma | dUC | TEM, WB | CD63, CD9, and CD81 | Exosomes from HIV-1-infected cells promote cancer growth and progression.  This effect is mediated through HIV TAR RNA carried by the exosomes. |
| (Fujiwara, Eguchi et al. 2018) | NA | HSC-3, human oral squamous (epithelial) cell line RT7 | cell culture supernatant | Total Exosome Isolation | TEM, WB | CD9, EGFR | OSCC-derived exosomes induce epithelial–mesenchymal transition (EMT) in epithelial cells, representing a novel mechanism of tumour progression and carcinogenesis.  The anti-EGFR antibody cetuximab can inhibit the EMT-inducing effects of these exosomes. |
| (Guerreiro, Vestad et al. 2018) | NA | human oral squamous cell carcinoma (PE/CA-PJ49/E10)), pancreatic adenocarcinoma (BxPC3), and a human melanoma brain metastasis (H3) | cell culture supernatant | UF ,SEC | NTA, immunoaffinity capture, flow, WB, TEM | CD63 | A novel method for isolation and seperation of Evs were described and validated  a robust, cost-effective method to isolate high-purity extracellular vesicles (EVs) from cell culture media using exosome-depleted serum, ultrafiltration, and size-exclusion chromatography, enabling reliable EV characterization, and supporting biomarker and therapeutic research. |
| (Xiao, Zhang et al. 2018) | NA | Human immortal oral epithelial cell line cells (HIOEC) and oral leukoplakia cell line cells (Leuk1), OSCC cell lines SCC25 and Cal27 | cell culture supernatant | UF, UC | NTA, | Alix, CD63, CD9, and Rab5 | Oral cancer-derived exosomes transfer THBS1, which polarizes macrophages into M1-like tumour-associated macrophages (TAMs).  Targeting M1-like TAMs may have therapeutic potential to control tumour cell migration in OSCC. |
| (Qu, Leung et al. 2021) | OTSCC=14, HC=10 | NA | blood plasma EV | SEC | TEM, NTA, WB | TSG101, CD9, negative marker Calnexin | deregulated EV proteins in plasma from patients with and without nodal OTSCC compared with non-cancer controls.43 unique candidate EV-associated proteins with deregulated expression pattern |
| (Theodoraki, Hoffmann et al. 2018) | parients =40, six healthy controls were male | NA | plasma | miniSEC | qNAno/ TEM, TRPS, WB, BCA protien | Tsg101 | Plasma-derived exosomes show potential as non-invasive cancer biomarkers.  Fractionating exosomes into CD3⁺ and CD3⁻/CD44v3-enriched subsets allows assessment of relative levels and functions of immunoregulatory proteins from different immune and non-immune cell populations. This approach provides insights into the immune landscape and tumour biology in cancer patients. |
| (Tamkovich, Yunusova et al. 2018) | colorectal cancer=20, HNSCC=10, Lung=5/healthy donors (n = 10 | NA | plasma | UF, UC | TEM, FCM, WB | CD81, Cd9 | Various exosome types circulate in blood plasma under normal conditions and during cancer development.  Tumour-associated exosomes can be enriched using immunosorption with antibodies against CD9, CD81, and proteins of 10–15 kDa, which serve as universal markers. Enriching for tumour-derived exosomes in plasma increases diagnostic sensitivity and specificity by reducing background from exosomes secreted by normal cells. |
| (Theodoraki, Yerneni et al. 2018) | 40 HNSCC patients | NA | plasma | SEC | qNano (Izon), TEM, WB, FCM, ELISA | CD63, pdl | Exosomal PD-L1 levels correlate with disease activity, UICC stage, and lymph node status in HNSCC patients. Plasma soluble PD-L1 (sPD-L1) and exosomal PD-1 levels do not show such correlations.  Exosomal PD-L1, but not sPD-L1, is associated with disease progression, making circulating PD-L1⁺ exosomes a promising biomarker for disease and immune activity in HNSCC. |
| (Wang, Qin et al. 2018) | NA | CAL 27 and SCC-25 | cell culture supernatant | UF, affinity chromatography | EM, NTA, immunoblotting. | CD63, CD9, heat shock protein 70 (HSP70), Ras-related protein 5 (Rab5), and ALG2-interacting protein (Alix) | Oral cancer cell-derived exosomes (OCEXs) enhance NK cell cytotoxicity.  This effect is mediated through the IRF-3 signaling pathway.  Exosomal NAP1 is identified as a key factor in promoting NK cell activation, revealing a novel mechanism of OCEXs’ immunomodulatory effects. |
| (Qadir, Aziz et al. 2018) | NA | 3 normal oral keratinocytes (OK113, NK4, NOK368) and 5 malignant (Ca1, CaLH2, SQCC/Y1, SVpgC2a and SVFN8) cell lines | cell culture supernatant | UC | SEM, TEM, Immuno-gold TEM, DLC, RT-Qpcr, NTA, WB | CD63, CD9 and CD81 | Both normal and cancer-derived exosomes can modulate gene expression pathways in recipient normal cells.  Cancer exosomes can reprogram the transcriptome of recipient cells, promoting angiogenesis, immune evasion, cell fate changes, and metastasis.  The study identifies molecular pathways and biomarkers that could be exploited for liquid-biopsy diagnostics and immunotherapy development. |
| (Principe, Mejia-Guerrero et al. 2018) | 69  OTSCC tissues | (primary CAF, AF and SCC25, SCC4) | cell culture supernatant | UC | TEM, WB, MASS, SPEC | MFAP5 | CAF-derived exosomes significantly increase proliferation and migration of OTSCC cells compared to exosome-depleted media, contributing to tumour progression.  MFAP5 is highly expressed in CAF-secreted fractions, including exosomes and conditioned media, suggesting it as a potential marker of the CAF phenotype in OTSCC. |
| (Momen-Heravi and Bala 2018) | 34  OSCC | CAL27, | cell culture supernatant and plasma | polymer precipitation  method with ExoQuick-TC™ | NTA, WB ELISA | miR-2, miR-27. | EVs are elevated in the plasma of OSCC patients and carry oncogenic miRNAs such as miR-21 and miR-27.  Circulating EVs reflect a tumour-derived miRNA signature, consistent with OSCC cell lines.  EVs are taken up by monocytes, triggering NF-κB activation and creating a pro-inflammatory, pro-tumorigenic environment with increased IL-6, CCL2, PGE2, and MMP9.  Inhibition of miR-21 in monocytes reduces this pro-inflammatory phenotype, highlighting its role in EV-mediated tumour-promoting inflammation. |
| (Li, Tao et al. 2018) | NA | Human oral keratinocytes (HOK), OSCC cells, including CAL27 and SCC15 | cell culture supernatant | sucrose-gradient centrifugation | TEM, WB | CD63 and GM130 | OSCC cells acquire a more aggressive phenotype by taking up CAF-derived exosomes lacking miR-34a-5p.  Transfer of miR-34a-5p regulates OSCC proliferation and motility via the AKT/GSK-3β/β-catenin/Snail signalling pathway.  Targeting the miR-34a-5p/AXL axis may represent a potential therapeutic strategy for OSCC. |
| (de Andrade, de Oliveira et al. 2018) | NA | (HUVEC), two cell lines of oral squamous cell carcinoma (OSCC) (SCC15 and HSC3) | cell culture supernatant | polymer-based precipitation (ExoQuick) | NTA, TEM | CD63 and Annexin II. | EVs from different OSCC cell lines have distinct effects on angiogenesis, with some promoting and others inhibiting endothelial cell responses.  SCC15-derived EVs stimulate angiogenesis, endothelial cell apoptosis, and migration, whereas HSC3-derived EVs have the opposite effects.  These results highlight the complexity of EV-mediated tumour–endothelial interactions and provide insights for future therapeutic strategies targeting EVs. |
| (Langevin, Kuhnell et al. 2017) | 5 patients with incident primary HNSCC  5 HD | H413 Detroit 562 FaDu Cal 27 Normal oral epithelial cells | cell culture supernatant, saliva | dUC | NTA, TEM, WB | CD81, TSG101 | Exosomal miRNA profiles differ significantly between HNSCC cells and normal oral epithelial cells, with substantial overlap among four HNSCC cell lines.  Several cancer-specific exosomal miRNAs identified in vitro are found at elevated levels in saliva from HNSCC patients compared to healthy controls.  These findings suggest the potential of salivary exosomal miRNAs as non-invasive biomarkers for HNSCC. |
| (Liu, Chen et al. 2017) | NA | cisplatin-resistant cell lines (HSC-3-R and SCC-9-R) | cell culture supernatant | dUC | TEM, qNano analysis, WB | CD63, CD81, PTEN, PDCD4, GAPDH | Exosomes from cisplatin-resistant OSCC cells transfer miR-21 to recipient OSCC cells, inducing cisplatin resistance by targeting PTEN and PDCD4.  miR-21 has diagnostic and prognostic value in cancer.  Targeting miR-21 or using it as a biomarker may improve therapeutic outcomes for cancer patients. |
| (Al-Samadi, Awad et al. 2017) | 3 healthy donors | Two human OTSCC cell lines, HSC-3, and SCC-25 | CM | two-step ultracentrifugation procedure | NTA, immuno-electron microscope | CD63 | EVs from SCC-25 cells enhanced cytotoxic activity of CD8⁺ T cells and NK cells more than EVs from HSC-3 cells.  SCC-25 EVs reduced IL-13 mRNA levels, suggesting a role in modulating immune responses. |
| (Overmiller, Pierluissi et al. 2017) | NA | HaCaT cells | cell culture supernatant and BLOOD | UC ExoQuick-TC/ Serum-derived EVs were isolated by preclearing circulating immunoglobulins with protein A Sepharose beads | NTA, WB | CD9 and CD63 | SCC EVs were enriched with the C-terminal fragment of desmoglein 2 (Dsg2), a cadherin often overexpressed in malignancies.  Dsg2 overexpression increased EV release and mitogenic cargo (EGFR, c-Src).  Inhibiting Dsg2 ectodomain shedding (via GM6001) led to accumulation of full-length Dsg2 in EVs and reduced EV release.  Fibroblast uptake of SCC EVs (tracked via GFP-tagged Dsg2) was confirmed; EVs activated Erk1/2 and Akt pathways, enhancing fibroblast proliferation.  In vivo, Dsg2 was upregulated in head and neck SCC, and patient serum EVs were enriched in Dsg2 C-terminal fragment and EGFR |
| (Menck, Bleckmann et al. 2017) | n=330(79 head & neck cancers, 74 lung cancers, 41 breast cancers, 28 colorectal cancers and 108 with other cancer forms/ control=103 (Healthy=63, nonhealthy=41) | Human MCF-7, MDA-MB-231 and SK-BR-3 breast cancer, A549 lung cancer as well as SW480 colorectal cancer cells | human peripheral blood sample | UC | FCM, WB, EM, NTA, qRT-pcr | Tublin, Hsp90, Tsg101, syntenin, CD81, CD9 | Tumour-associated MVs carrying MUC1, EGFR, and EpCAM were elevated in a subtype-specific manner, while EMMPRIN+ MVs were increased across all tumour types and linked to poor survival. Combining all four markers distinguished patients from healthy controls (AUC ≤ 0.85). Functionally, cancer patient MVs promoted tumour-supportive macrophages and enhanced invasion via glycosylated EMMPRIN. |
| (Maybruck, Pfannenstiel et al. 2017) | NA | Human colon carcinoma cell line Caco-2 and squamous cell carcinoma (SCC) of the head and neck cancer cell lines: Tu167, SCC0209, HN60 | cell culture supernatant | UC | Mass spectrometry | Gal-1, HSP70, Lamin A, b-Actin | Tumour-derived exosomes (TDEs) from multiple head and neck cancer cell lines induce CD8+ T cell formation, primarily via galectin-1. |
| (Yunusova, Tamkovich et al. 2017) | 2O ovarian, 20 colorectal, 8 squamous cell head and neck carcinoma  10 healthy  donors | NA | blood and ascites | UC | TEM, FCM | CD9, CD63, CD81 and CD24 | Exosome subpopulations in patient biological fluids were similar across cancer types, but protein concentrations varied. |
| (Rabinowits, Bowden et al. 2017) | 5 head and neck SCC patients | NA | Plasma | ExoQuick serum | TaqMan-Based miRNA Profiling | miRNA | Aberrant miRNA expression was detected in both tumour tissue and plasma of tongue SCC patients, indicating potential as a biomarker. Circulating exosomes provided a more reliable means of assessing tumour-miRNA expression. |
| (Yuana, Jiang et al. 2017) | NA | FaDu | cell culture supernatant | without any isolation or purification | FCM, immuno-magnetic bead capture assay, immunogold EM, WB | CD9, CD63, and alix, tsg101 | USMB treatment of FaDu cells triggered EV release via exocytosis.  These EVs were heterogeneous in size and displayed CD9, CD63, and/or phosphatidylserine.  In untreated cells, EV markers Alix, TSG101, CD9, and CD63 were below detection limits. |
| (Mutschelknaus, Azimzadeh et al. 2017) | NA | human cell lines BHY, FaDu | cell culture supernatant | UC | TEM, NTA | ALIX, TSG101, GAPDH | Exosomes from irradiated head and neck cancer cells enhanced recipient cell migration via increased AKT signalling.  Proteomic analysis identified radiation-regulated exosomal proteins as potential drivers, though other exosomal components may also contribute.  Clinically, this exosome-mediated communication could promote metastasis during radiotherapy, making it a promising target to improve treatment outcomes. |
| (Li, Li et al. 2016) | One hundred and eight patients with newly diagnosed OSCC, 108 volunteers without any malignancy | OSCC cell lines SCC-9 and CAL-27 | cell culture supernatant | ExoQuick exosome precipitation | SEM, qRT-PCR, Immunofluorescence and immunohistochemistry, WB | CD81, CD63, Tubulin | Hypoxia elevates miR-21 in tumour-derived exosomes, which are taken up by normoxic cells and promote a prometastatic phenotype.  These exosomes may function as messengers, transferring miRNAs between hypoxic and normoxic OSCC cells to remodel the tumour microenvironment. |
| (Sakha, Muramatsu et al. 2016) | NA | HOC313-P, TSU as well as HeLa cells | cell culture supernatant | SEC | WB, immunogold-TEM, Fluorescence microscopy, 3D confocal microscopy | CD63, CD9, CD81 | Exosomal miR-1246 promotes cell motility and invasion by suppressing DENND2D.  Loss of DENND2D enhances migration and invasion in HOC313-P, TSU, and HeLa cells. |
| (Languino, Singh et al. 2016) | NA | Primary keratinocytes and fibroblasts | cell culture supernatant | dUC | Immunoblotting, | TβRII, TSG101, CD9 | Fibroblast-derived exosomes restore TGFβ signalling in SCC keratinocytes lacking TβRII, which are otherwise unresponsive to TGFβ ligand.  This highlights a potent tumour–stroma communication mechanism enabling reactivation of TGFβ signalling in SCC. |
| (Vered, Lehtonen et al. 2015) | TSCC n = 64 | HSC-3 cells | cell culture supernatant | ExoQuick | ELISA, WB, and immuno-electron microscopy | CD63, CD9 and CD81 | CAV1 accumulation in the tumour microenvironment predicts poor prognosis.  CAV1 is present in cancer cells during EMT and in fibroblasts transitioning to CAFs, with its delivery to the TME mediated by cancer cell–derived exosomes. |
| (Winck, Ribeiro et al. 2015) | 9 healthy  21 oral cancers | NA | saliva | UC | Immunoblotting, TEM, NTA MASS SPEC | Proteins | Proteomic analysis revealed that salivary EVs in OSCC contain proteins linked to humoral immune responses, inflammation, metal transport, and cell growth/proliferation.  Saliva from OSCC patients — even without visible lesions — showed overexpression of cancer-related proteins compared to healthy controls.  The exact mechanisms by which saliva components and immune cells modulate tumour cell responses remain unclear, but EV-mediated communication may play a key role in immune–tumour interactions that promote tumour growth. |
| (Kim, Wieckowski et al. 2005) | 27 patients, 20 normal donors | OSCC cell line, PCI-13 | sera and cell culture supernatant | SEC, UC | TEM, WB, immunoelectron microscopy | FasL | Sera from OSCC patients often contain FasL-positive microvesicles carrying 42 kDa FasL, which trigger both receptor- and mitochondria-mediated apoptosis in T cells (caspase-3 activation, cytochrome c release, loss of mitochondrial potential, and reduced TCR-ζ expression).  Their activity is partially inhibited by anti-Fas antibody (ZB4), and FasL+ microvesicle levels correlate with tumour burden and nodal involvement, suggesting a role in tumour immune evasion. |
| (Hong, Funk et al. 2016) | 3 AML  3 HNSCC  3 NC | NA | plasma | mini-SEC | qNano, TEM, WB, BCA protein assay kit | CD9, TSG101 | Plasma-derived exosomes showed a greater tendency to aggregate than exosomes from AML (Kasumi) or HNSCC (PCI-13) cell lines when isolated via mini-SEC.  The mini-SEC method offers a simple, low-cost, one-step approach that can partially remove debris and produce morphologically intact, immunoglobulin-depleted plasma exosome fractions capable of mediating intercellular communication. |
| (Man, Li et al. 2023) | 63 OSCC,  24 healthy donors  24 oral dysplasia patients | CAL27 | salivary, cell culture supernatant | dUC | TEM, WB, proteomics, FCM, NTA, Mass spec | AXL, EGFR, CD8, GAPDH | Elevated non-apoptotic tumoral microvesicles (S-MVs) were observed in OSCC, with significantly higher levels in T3–T4 stage patients compared to T1–T2 stages or healthy controls.  In an OSCC mouse model, these MVs correlated with tumour volume, and EGFR overexpression increased their production, enhancing normal epithelial cell migration.  The findings suggest that non-apoptotic tumoral MVs, intricately linked to EGFR signalling activation, are a promising progressive biomarker for OSCC. |
| (Flemming, Hill et al. 2020) | NA | Dsg2 mutant cell lines, Human A431 (epidermoid skin carcinoma) or UMSCC1 (oral SCC | cell culture supernatant | UC | WB, NTA, RNAseq, RT PcR | CD63 and CD9, GAPDH | Intercellular communication via cell–cell adhesion, cytokine release, and EV secretion is coordinated and essential for tumour growth and progression, and these processes may serve as prognostic markers to guide treatment strategies. |
| (Bernareggi, Xie et al. 2022) | NA | GSC lines 387, CW468, D456, and 1517 / Cal27, Detroit568 and UMSCC17B | cell culture supernatant | ExoQuick and UC | FCM, TEM | CHMP2A, GAPDH | Deletion of CHMP2A in CAL27 HNSCC cells enhances NK cell–mediated killing in a xenograft model, revealing that EV secretion contributes to tumour immune escape and suggesting that targeting CHMP2A could improve NK cell–based immunotherapy. |
| (Jelonek, Wojakowska et al. 2015) | NA | FaDu | cell culture supernatant | Total Exosome Isolation Kit, UC | WB, TEM, MS | CD63 | Exosomes isolated from human HNSCC cells showed lower purity and heterogeneity, with some contamination from FBS peptides.  Exposure to ionizing radiation significantly altered their protein composition: certain proteins involved in transcription, translation, cell division, and signalling were up- or downregulated, indicating that exosomal cargo reflects radiation-induced cellular changes, such as transient suppression of transcription/translation or stress signalling. |
| (Dong, Zhang et al. 2023) | NA | HEK293 cells, OSCC cell lines SCC084/R, SCC131/R, and SCC9/R | cell culture supernatant | UC | TEM, DLS, WB | Positive TSG101 and CD63 expressionscalnexin was negative | Exosomal circNR4A1 enhances OSCC sensitivity to anticancer drugs, by modulating the K-RAS/ERK and p53 signalling pathways. |
| (El‐Labban and Osorio‐Herrera 1986) | 10 cases squamous cell carcinoma taken from buccal mucosa, tongue, and larynx |  |  |  | Light Microscopy, EM |  | The findings align with previous reports on civatte bodies in oral lichen planus, now recognized as a form of apoptotic body, and with descriptions of individual cell keratinization in Bowen’s disease and sunburn cells. Unlike earlier observations, these changes occur not only in the S phase but also in cells undergoing mitosis. |
| (Yuan, Wang et al. 2021) | Fifty-two combined OSCC and paracancerous samples | Two OSCC cell lines (HN4 and HN6) | cell culture supernatant | UC | TEM, NanoSight, PALS | TSG101, CD63 | TAM-derived exosomes carrying miR-31-5p promote OSCC cell proliferation and development by downregulating LATS2 and inactivating the Hippo signalling pathway.  These exosomes may represent a potential therapeutic target, but further studies are needed to clarify their role in OSCC metastasis and tumour microenvironment. |
| (Man, Li et al. 2023) | 61 OSCC | NA | Saliva | dUC | TEM, FCM.NTA, WB | CD4, CD8, EGFR | A higher CD8-to-EGFR ratio in S-MVs is associated with better prognosis in OSCC patients and may serve as a potential positive prognostic indicator. |
| (Omori, Noguchi et al. 2024) | NA | Ca9-22, OSC20, OSC19, and Sas | cell culture supernatant | ExoQuick™ Exosome Pre-cipitation kit ( | WB, TEM | TSG101 and CD63 | PD-L1 contributes to invasion and metastasis of oral cancer through partial EMT (pEMT), with its expression induced by bacterial LPS and TGF-β. OSCC-derived exosomes exposed to LPS exhibit high PD-L1 levels, suggesting that exosomes may function as carriers of PD-L1. |
| (Miao, Liu et al. 2024) | NA | NIH3T3 cells and SCC7 | cell culture supernatant | Exosome Isolation Kit (084,001, Beibei Biotechnology | NTA, BCA, TEM, WB, LSMS | TGF-β1, GAPDH and Alix | OSCC-derived EVs play a significant role in driving fibroblast differentiation into metastasis-associated CAFs (mCAFs) and in modulating specific collagen types within the extracellular matrix (ECM). |
| (Apeltrath, Simon et al. 2024) | 50 HNC Pt | NA | serum | miRCURY Exosome Serum/Plasma Kit | Reverse Transcription, Real-time PCR amplification of cDNA | EV-miR-21, -1246, -200c, -let-7a, -11a, and -26 | EV-miR-21 remains a promising tumour marker and provides new insights into EV-miR-181a and EV-miR-let-7a in relation to HPV-associated HNSCC, which is becoming increasingly prevalent. |
| (Li, Wang et al. 2024) | 30 OSCC patients | NA | serum | UC | TEM, WB, NTA | miRNA let-7c- 5p | let-7c-5p is upregulated in serum-derived exosomes and promotes proliferation, migration, and invasion of cancer cells by downregulating TAGLN expression. |
| (Huang, Chou et al. 2024) | NA | OSCC cell lines (OEC-M1 and TW2.6) | cell culture supernatant | UC | TEM, NTA, WB | CD9  and CD81, miR-421 | lncRNA MEG3 functions as a tumour suppressor by regulating endothelial angiogenesis via the exosomal miR-421/HS2ST1 axis. |
| (Yu, Wu et al. 2024)) | 37 patients with recurrent/metastatic oral squamous cell carcinoma. | NA | Saliva, cell culture supernatant | UC | NTA, TEM, WB, Nano-FCM | CD45, EpCAM, and PD-L1 | In OSCC patients, I-PD-L1⁺ EVs are reduced compared to healthy individuals, but levels of heterogeneous PD-L1⁺ EVs vary across patient groups. During immunotherapy, responders show decreased total PD-L1⁺ EVs, T-PD-L1⁺ EVs, and I-PD-L1⁺ EVs, whereas nonresponders exhibit increased I-PD-L1⁺ EVs. Detection using a step-wedge microfluidic chip enables simultaneous monitoring of PD-L1⁺ EV subtypes, improving the prediction of immunotherapy outcomes. |
| (Bugajova, Raudenska et al. 2024) | 41  of oral squamous cell carcinoma (OSCC) patients (P1-P10) and healthy volunteers (H1-H10) | FaDu (HPV− HNSCC line) | Blood serum, Cell culture supernatant | exoRNeasy Maxi Kit (Qiagen) | RNA-seq, TEM, WB | CD9, TSG101, EpCAM, Cav-1, flotillin, annexin V | Elevated mtRNAs were found in EVs from oropharyngeal HNSCC compared with other HNSCC sites. Glutamine (Gln) starvation increased oxidative stress, leading to more mtRNA packaging into EVs. No significant difference in mtRNA levels between HNSCC patients and healthy controls, due to the use of ISEV-compliant EV isolation methods. |
| (Xie, Zhong et al. 2024) | 17 | Primary buccal oral mucosa fibroblast, HaCat cell lines and HEK293T cells | cell culture supernatant | dUC, exosome isolation reagent | NTA, WB, dio staining | CD9, CD63, CD81 | Arecoline exposure induces the release of exosomes enriched in miR-17-5p. These exosomes promote collagen deposition and drive fibroblast differentiation into myofibroblasts, suggesting a mechanism by which arecoline contributes to oral fibrotic or tumour-supportive microenvironments. |
| (Sato, Toh et al. 2025) | Part 1: N=17 (RNA-seq); Part 2: N=20 (qPCR validation) | NA | plasma | UC | RNA-seq (discovery), RT-qPCR (validation), IHC (HLA-E protein) | HLA-E mRNA (primary biomarker) | High PEX HLA-E mRNA associated with poor survival (0% 2-year OS in high-score group). Proposed dual checkpoint blockade (anti-PD-1 + anti-NKG2A) for non-responders. |
| (Pande, Bettadaiah et al. 2024) | NA | KB-3-1, HEp-2 | CURD | UC | TEM, SEM, DLS, MS | Phospholipids, neutral lipids, fatty acids (GC-MS) | Exo-CAA showed higher bio accessibility (3.78%) vs free CAA (1.81%) Reduced IC50 in HNC cells, Downregulated EphA2/STAT3, induced apoptosis |
| (Liu, Zhao et al. 2024) | NA, through the TCGA-GDC portal | NOK, SCC-4, and CAL-27 | cell culture supernatant |  | (qRT-PCR) | LINC01564 | LINC01564 overexpression in HNSCCKnockdown reduces proliferation/invasion, correlates with poor prognosis, and altered immune microenvironment |
| (Theodoraki, Huber et al. 2024) | 29 HNSCC patients | NA | plasma | UC | NA | PD-L1, PD-L2, CTLA-4, TIM-3, LAG-3 | HNSCC exosomes increase M2 macrophage markers (CD206) Upregulate PD-L1/CTLA-4 on macrophages, Induce CXCL4 secretion |
| (Yang, Zhang et al. 2024) | In vitro: HSC6/CAL27 cell lines In vivo: n=24 BALB/c mice (6/group | HSC6, CAL27 (OSCC) | cell culture supernatant | UC, TER | NanoFCM  WB  LC-MS/MS | SLC1A5 (glutamine transporter, CD63, CD9, TSG101 | 2Gy-sEVs increase proliferation in recipient cells Deliver SLC1A5 to enhance glutamine uptake SLC1A5 inhibition (V-9302) reverses pro-tumour effects |
| (Xu, Cheng et al. 2024) | 48 HNSCC tissue samples | HN4, HN6, CAL27 | Tumour tissues, cell culture supernatant | UC | WB, functional assays | ACLY | sEV-ACLY acetylates p65 to upregulate CXCR1  BA (ACLY inhibitor) blocks transformation  Clinical correlation of ACLY/CXCR1 |
| (Rubenich, Domagalski et al. 2024)(2024) | Patients: n=76 (34.2% F, 65.8% M)  In vitro: 5 biological replicates | UMSCC47, FaDu, SCC9, PCI52 | Tumour tissues, plasma, cell culture supernatant | UC, mini-SEC (Sepharose CL-2B) | TRPS SEM   WB | CD9, TSG101, HSP70 (negative for Calnexin) | NLR increases with HNSCC stage (p<0.001 stage IV vs I), TEX from SCC47 cell line upregulate neutrophil CD73/PD-L1 -CD73 inhibition blocks TEX-mediated immunosuppression. |
| (Ha, Choi et al. 2025) | NA | FaDu, UMSCC1 | Bacterial EVs | UC - DOUBLE CHECK | NTA, TEM, RNA-seq, qRT-PCR, functional assays | miR-146a | Aa-EVs upregulate IL-6/CXCL8/VEGFC (RNA-seq) - Induce miR-146a-5p (3.94-2.23x) - Enhance invasion (Matrigel assay) Promote HNSCC progression via: Cell cycle acceleration (S/G2) and miR-146a-mediated invasion |
| (Metsäniitty, Hasnat et al. 2024) | NA | HSC-3 (OSCC cell line) | Bacterial EVs, | UC | Functional assays - Tumour size, metastasis quantification | LPS O-antigen | CDT-deficient EVs increased tumour area  LPS O-antigen-deficient EVs reduced metastasis  P. gingivalis EVs showed no significant effects |
| (Lv, Liu et al. 2024) | In vitro: ≥3 replicates In vivo: n=6 mice/group Clinical: 60 patients (30 with/without LN metastasis) | SCC-25, CAL-27 (OCSCC), CAFs, normal oral epithelial cells | cell culture supernatant | UC | TEM, NTA WB | CD81/TSG101/FLOT1/ALIX, Calnexin | CAF-EVs promote OCSCC proliferation/migration/invasion  BMI1/ITGB1 knockdown reverses pro-tumour effects  High BMI1/ITGB1 correlates with LN metastasis and poor prognosis (OS/RFS) |
| (Søland, Lipka et al. 2024) | NA | - NOFs (normal oral fibroblasts) - OSCC (PE/CA-PJ49/E10) - PDAC human pancreatic adenocarcinoma  - MBM (melanoma brain metastasis) | cell culture supernatant | UC | TEM, WB, NTA, Fluorescent Labelling (Dil): | CD81, TSG101, FLOT1, ALIX - calnexin | EV effects are cancer-type dependent:  OSCC-EVs: increased proliferation (short-term) and upregulated ANO1/KCNMA1  PDAC-EVs: decreased viability but increased migration at low doses  MBM-EVs: increased proliferation at 24 h, variable migration effects  CAF activation: All EVs upregulated CAF markers (ACTA2, FAP, PDGFR)  Dose response: Non-linear effects observed, e.g., migration inhibition at highest EV doses |
| (Li, Cai et al. 2024) | OSCC patients |  | Postoperative drainage fluid (PDF) from OSCC patients | Antibody-grafted microbeads (immunoaffinity), Microfluidic chips | TEM, SEM, NTA, WB, LC-MS/MS | CD9, ALI, GM130, Calnexin | ASPH⁺ PDF-EVs elevated in OSCC patients with lymph node metastasis (LNM).  Microfluidic immunoaffinity isolation achieved >90% capture and release efficiency for ASPH⁺ EVs.  ASPH expression correlates with poor OSCC prognosis (validated by TCGA data and IHC). |
| (Inyang, Evans et al. 2024) | HPV+ HNSCC patient biopsies (IHC for TRPV1) Plasma-derived sEVs from HPV+ vs. HPV- patients | HNSCC cells (e.g., UM-SCC-47) HPV- HNSCC cells (negative controls) Normal oral keratinocytes (additional controls) | Plasma | UC | TEM, WB, NTA | CD63+, TSG101+, Alix+ | HPV⁺ HNC sEVs are specifically pro-nociceptive compared to HPV⁻ sEVs.  TRPV1⁺ neurons are the main targets of sEV-mediated pain signalling.  sEV-associated miR-34a-5p induces TRPV1 overexpression, leading to hyperalgesia. |
| (Li, Lin et al. 2024) | NA | Cal 27, SCC25 | cell culture supernatant | UC | TEM, NTA | CD63+, TSG101+ | sEV miRNA signature is associated with lymph node metastasis and poorer survival in patients in TCGA-HNSC dataset. |
| (Li, Yin et al. 2023) | NA | SCC7 | cell culture supernatant | UC | TEM | GCGC-motif miRNAs (e.g., miR-24-1, miR-103b, miR-127) | GCGC-motif miRNAs (e.g., miR-24-1, miR-103b, miR-127) are selectively packaged into OSCC-derived exosomes.  These exosomal miRNAs promote lymph node metastasis by targeting NK cells via TGF-β signalling.  They serve as potential diagnostic biomarkers (82% sensitivity) and prognostic indicators (HR = 2.98) |
| (Fu, Xiong et al. 2024) | samples of residual peripheral blood for routine blood tests before sur- gery (n = 48), saliva (n = 46), and lymphatic fluid (n = 44) were collected from the OSCC patients (n = 52). In addition, 191 biopsies were collected from the patients underwent surgical resection. | NA | tissue, saliva and blood, lymphatic fluids | UC, for tissue mor steps needed | WB, TEM, NTA, DLS, MS | (CD81, CD9, CD63, TSG10 | Tumour-derived small extracellular vesicles (Ti-sEVs) in OSCC show distinct spatiotemporal distributions linked to patient prognosis.  Low PT-sEVs/Tu-sEVs ratio identifies early-stage OSCC patients at elevated risk of recurrence.  High PT-sEVs/Tu-sEVs ratio predicts responders to anti-PD-1 immunotherapy.  While promising for clinical stratification, further work is needed to simplify the method and clarify Ti-sEV origins, trafficking, and immunotherapy effects. |
| (Zhou, Zhou et al. 2024) | NA | CAL27  B16-F10 Raw 264.7 | cell culture supernatant | Total Exosome Isolation Kit, UF | DLS,  TEM, SEM. FT-IR, HPLC, NMR (for GT-RG). | Golgi-targeted ROS generator and a CSF1R inhibitor | The study demonstrates that synthetic EVs loaded with a Golgi-targeted ROS generator and a CSF1R inhibitor (GT-RGEV@CSFRi) can significantly enhance the efficacy of photodynamic therapy and immunotherapy in tumours. These engineered EVs increase ROS-induced apoptosis in cancer cells, reprogram tumour-associated macrophages toward an anti-tumour phenotype, and promote CD8⁺ T-cell infiltration, all while exhibiting no systemic toxicity. This approach highlights the potential of multifunctional, targeted EV-based therapies to improve anti-cancer immune responses and therapeutic outcomes. |
| (Metsäniitty, Hasnat et al. 2024) | NA | SCC-24A, HSC-3 cells | cell culture supernatant | UC | NanoDrop 100 spectrophotometer, NTA, Silver Stain protein gel electrophoresis |  | EVs derived from periodontopathogens may modulate the behaviour of oral cancer cells, exerting either inhibitory or stimulatory effects. Notably, in vitro findings suggest that EVs from Aggregatibacter actinomycetemcomitans may have a potential antitumorigenic effect on metastatic oral squamous cell carcinoma (OSCC) cells. |
| (Yan, Liu et al. 2024) | Thirty HNSCC tissue samples | CAL27, SCC25 | cell culture supernatant | UC | WB, FCM, NTA, TEM | TSG101, CD63, CD9 | TAMs promote angiogenesis in HNSCC by transferring exosomal miR-21-5p to endothelial cells, activating the YAP1/HIF-1α axis |
| (Liu, Mu et al. 2025) | NA | HSC-3, aDu, CAL-27, and THP-1 | cell culture supernatant | ExoQuick-TC kit | TEM, NTA, WB | ALIX,CD81, and CD9 | An increase in MIR4435-2HG levels was observed in tumour-associated macrophages (TAMs), and TAM-derived exosomes promoted migration, invasion, and epithelial-mesenchymal transition (EMT) marker expression in HNSCC cells by upregulating MIR4435-2HG. |
| (Ding, Chen et al. 2024) | NA | SCC cell lines (CAL27, Tca-8113, and SCC-25) | cell culture supernatant | UC | NTA, WB, TEM | CD9 and CD63 | TSCC cells secrete miR-558, which is delivered to HUVECs, where it enhances angiogenesis by directly regulating HPSE expression, thereby promoting tumour vascularization. |
| (Duan, Zhou et al. 2024) | 161 cases of HNSCC tissue specimens and 54 cases of preoperative plasma | SCC15 and SCC2, HN4, HN6, and HN30 | cell culture supernatant | UC | TEM, NTA | miR-5100 | Hypoxic conditions in HNSCC upregulate miR-5100 via HIF1α-mediated transcription, leading to QKI suppression in tumour cells and fibroblasts, which promotes tumour invasion and CAF activation, suggesting miR-5100 as a potential diagnostic and therapeutic target. |
| (Pelaez-Prestel, Gonzalez-Martin et al. 2024) | NA | H413 and TR146 | cell culture supernatant | tangential flow filtration, SEC | microBCA Protein Assay Kit, WB | CD81 | OSCC cell–derived conditioned medium (CM) drives monocyte differentiation into immunosuppressive TAM-like macrophages, potentially mediated by proteins such as PAI-1, FH, and VEGFA, although further proteomic validation and knockout experiments are required. Transcriptomic profiling revealed upregulation of immune-related genes, including IL2RA (CD25), suggesting a role for IL-2 signaling in their immunosuppressive function. These findings highlight the need for in vivo studies and analyses of human OSCC samples to confirm the relevance of this macrophage polarization in the tumour microenvironment. |
| (Yadav, Chaudhary et al. 2024) | Cancer Genome Atlas (TCGA). A total of 558 data- sets of primary HNSCC tumour (n = 514) and normal head and neck tissues (n = 44) | HPV-negative (OCT1) and HPV16-positive (93VU147T, UDSCC2 | cell culture supernatant + tissue | Total Exosome Isolation kit then Exosome Quantitation Kit | TEM, WB | HPV, HSP70 and TSG101 | Exosomes from HPV-positive and HPV-negative HNSCC cells carry distinct transcript profiles that may modulate protein synthesis and degradation in recipient cells. Notably, they also contain a unique set of transcripts associated with pathways linked to neurological disorders, according to TCGA analyses. |
| (Zhou, Liu et al. 2024) | tumour tissue samples of 23 OSCC from patients and 10 normal oral mucosa tissues from patients with no pathological evidence of OSCC | UM-SCC6 and CAL-27, | cell culture supernatant | UC | WB, TEM NTA, FCM, WB | VEGF , CD9, CD63 | Targeting HSPGs with specific antagonists may help overcome OSCC resistance to EV-associated VEGF and could represent a promising alternative strategy to conventional anti-VEGF therapy. |
| (Seki, Yamana et al. 2024) | 37 patients  blood patients with R/M  OSCC who were treated with immune checkpoint inhibitors (ICIs)  10 healthy volunteers | NA | blood |  | enzyme-linked immunosorbent assay PDL-1 | PDL-1, CD9 | Lower pretreatment serum PD-L1–positive extracellular vesicle (EV) levels are associated with longer overall survival in recurrent/metastatic OSCC patients receiving immune checkpoint inhibitors, suggesting that high PD-L1 EV levels may indicate T-cell exhaustion and poor prognosis, and highlighting their potential as both a prognostic biomarker and a therapeutic target. |
| (Cheng, Xie et al. 2024) | blood samples of oral squamous cell carcinoma (OSCC) patients.  (P1-P10) and healthy volunteers (H1-H10 | SCC25 cell culture | cell culture supernatant | UC | TEM, NTA | CD9, CD81, GAPDH, EpCAM, GM130, TSG101 | A novel approach integrates cholesterol-EpCAM proximity ligation–induced MNAzyme assembly with lanthanide-labelled ICP-MS detection, enabling separation-free, highly selective analysis of cancer-derived exosomes in human plasma, offering potential for early cancer diagnosis and adaptability to various tumour types through protein-specific aptamers. |
| (Wu, Shan et al. 2024) |  | Cal-27, SCC-25, and SCC-1 | cell culture supernatant |  | TEM | CD81, CD63, ALIX, TSG101 | A targeted RGD-modified exosome system (ID@RGD-EXO) was developed for precise oral cancer imaging, chemo phototherapy, and metastatic lymph node ablation, showing improved therapeutic efficacy, minimized toxicity, and enhanced surgical outcomes. |
| (Rizwan, Mahjabeen et al. 2025) | blood samples from400 verified HNC and  400 age- and gender-matched healthy individuals as control | NA | blood | a total exosome isolation kit, UC | TEM, NTA immunoelectronmicroscopy, ELISA | CD9, CD63, and CD81, miR-17,miR-20a and TGFBR2 | In head and neck cancer (HNC) patients, serum exosomes showed downregulation of miR-17 and miR-20a and upregulation of TGFBR2, which were associated with increased oxidative stress and disease aggressiveness, highlighting their potential as diagnostic and prognostic biomarkers as well as therapeutic targets. |
| (Li, Liu et al. 2025) | NA | Jurkat, NK-92MI | cell culture supernatant | UC | EVs- Containing Droplets |  | This study is the first to characteriSe the heterogeneity of small extracellular vesicles (sEVs) in HNSCC at droplet resolution, uncovering distinct mRNA and lncRNA cargo origins, and showing that T cell-derived sEVs can enhance NK cell cytotoxicity, while emphasizing the need for in vivo validation and improved isolation methods for future translational applications. |
| (Yang, Yang et al. 2025) | NA | SCC9 | cell culture supernatant | UF | TEM, Nano-FCM | CD9, CD63, Cd81 | Synthesized AMNCs were combined with NK cell-derived exosomes to create Exo-AMNCs, which effectively induced ferroptosis in OSCC cells via the FTO/GPX4 axis, significantly suppressing tumour growth in vivo. |
| (Madhu and Kannan 2025) | NC and HC serum samples | HEp-2 control and 4EBP1 KD | cell culture supernatant, Serum | Exoquick precipitation solution | DLS, TEM, WB | cd9, Alix, drp1, 4ebp1 | This study is the first to identify 4EBP1 in serum exosomes of head and neck cancer (HNC) patients, showing its upregulation compared to healthy controls and its role in promoting tumour growth and migration through DRP1/FIS1/TGFβ signalling; knockdown of 4EBP1 suppresses malignancy by restoring PTEN, underscoring its potential as both a diagnostic biomarker and therapeutic target. |
| (Liu, Jiang et al. 2025) | NA | MOC2 cells | cell culture supernatant | UC | TEM, particle sizer, WB | CD47, E-cadherin | Quercetin and chlorin e6 co-loaded extracellular vesicles (CQ@EVs) effectively suppress OSCC tumour growth by enabling targeted, light-activated drug delivery, inducing immunogenic cell death, enhancing antitumor immune responses, and reducing PD-L1 expression on cancer cells. |
| (Hu, Shu et al. 2025) | NA | SAS, Ca9-22, and TW2.6. | cell culture supernatant | total exosome isolation kit | NTA, WB | miR-320d | Downregulation of exosomal miR-320d by GBP5 may lead to upregulation of FAM49B, thereby promoting OSCC tumour growth and progression. |
| (Xia, Wang et al. 2025) | Tissue samples of 175 OSCC  patients and 25 healthy | HEK293T and CAL27 | cell culture supernatant, Tissues | UC | NTA, TEM, WB | CD9, CD63, GABDH | PCBP2 is the first protein identified as mediating EGFR-driven miRNA sorting into extracellular vesicles, providing new insights into tumour angiogenesis and suggesting potential diagnostic and therapeutic applications in epithelial cancers, pending further validation. |
| (Yadav, Tripathi et al. 2025) | NA | HPV16-positive (93VU147T, UDSCC2) and HPV-negative (OCT1) HNSCC cell lines | cell culture supernatant | Total Exosome Isolation kit | TEM, DLS | NA | HPV-negative tumour-derived exosomes are efficiently taken up by macrophages, promoting M1 polarization through STAT1, NF-κB, and AP1 signalling pathways, highlighting a novel mechanism by which tumour exosomes modulate macrophage-mediated dynamics within the HNSCC tumour microenvironment. |
| (Wang, Xiao et al. 2025) | NA | HSC3 and HEK 293 T | cell culture supernatant | UC | NTA, TEM, WB, MS | CD9, CD81, CD63 | Hypoxia, via the HIF-1α/HRS signalling pathway, enhances endosomal system activity, thereby promoting small extracellular vesicle (sEV) secretion, offering new insights into the complex interactions between hypoxia and the tumour microenvironment. |
| (Ko, Peng et al. 2025) | Forty-nine patients diagnosed with primary OSCC Surgical specimens and blood  samples | SAS/TW2.6 | cell culture supernatant | UC | TEM, WB, FCM | CD9, HSP70 | Arecoline-induced extracellular vesicles (EVs) promote DNMT-mediated methylation of the tumour suppressor gene PAX1, leading to its downregulation and contributing to OSCC progression and immune evasion via the IFIT1/PD-L1 signalling pathway, highlighting the PAX1/IFIT1/PD-L1 axis as a potential therapeutic biomarker. |
| (Chen, Song et al. 2025) | 11 patients with HNSCC and six patients without cancer | AMC-HN-8, FaDu | tissue, serum | ExoEasy Maxi Kit | TEM, MS, WB, RNA qpcr | CD9, TSG101 and CD63 | This study provides a preliminary characterization of the metabolic profile of HNSCC, identifying spermine and its synthetic pathways as potential diagnostic biomarkers and therapeutic targets. |
| (Lipka, Søland et al. 2025) | NA | human oral squamous cell carcinoma line (PE/CA-PJ49/E10; ECACC, NOF | cell culture supernatant | UF, SEC | NTA, TEM, WB, FCM | CD9 positive | OSCC-derived EVs enrich metabolic pathways such as glycolysis, the citric acid cycle, and amino acid metabolism in normal oral fibroblasts (NOFs), suggesting a phenotype switch that promotes a pro-tumorigenic state. |
| (Zhao, Dong et al. 2025) | oral cancer lesion; n = 73) and healthy volunteers with oral ulcer(s) (n = 62) |  | saliva. | dUC | DLS, TEM. FCM | NA | Salivary apoptotic microvesicles are significantly elevated in patients with non-healing oral ulcers and confirmed oral cancer, correlating with key clinicopathological features, and may serve as promising non-invasive biomarkers for early detection and disease monitoring. |
| (Wang, Wang et al. 2025) | NA | SCC7 and HOK | cell culture supernatant | UC | TEM, NTA, WB, fluorescence imaging | tsg101, CD9, CD81 | TDN-engineered M1 macrophage-derived extracellular vesicles (TDN@EVs) enhance antitumor protein delivery, induce ferroptosis through HSC70-mediated GPX4 degradation, and exhibit strong therapeutic potential for OSCC, with high stability and biosafety. |
| (Li, Chen et al. 2025) | NA | CAL-27 cell line | cell culture supernatant | UC | TEM, XPS, FTIR, CLSM, NTA, ICON AFM, WB, N30E nano flow analyzer | CD63, CD9, TSG101, and HSP70 | A label-free aptasensor using a “one exosome to multiple carbon dots” signal conversion and nano-micro dual-scale amplification enables sensitive, selective detection of OSCC-derived exosomes in artificial saliva, offering good linearity, low detection limits, anti-interference capability, and simple fabrication—showing strong potential for non-invasive, community-level OSCC and other cancer screening. |
| (Zhang, Liu et al. 2025) | OSCC and adjacent non-cancerous tissue | HSC3, HSC6, and 293 T human cell lines | cell culture supernatant | dUC | TEM, NTA, WB | CD81, TSG101, and CD63 | The study demonstrates that oral squamous cell carcinoma (OSCC) cells accept miR-148b-3p-depleted exosomes from cancer-associated fibroblasts (CAFs), leading to reduced miR-148b-3p levels in OSCC cells. This decrease promotes tumour aggressiveness by suppressing cuproptosis (a copper-induced cell death pathway) through upregulation of ATP7A, while also enhancing malignant behaviours. The findings highlight the miR-148b-3p/ATP7A axis as a potential therapeutic target for oral cancer treatment. |
| (Bano, Vats et al. 2025) | Saliva samples were collected from non-smokers (n = 20), smokers (n = 20), and patients with oral cancer (n = 20). | NA | saliva | total exosomal kit | (HPLC), (FTIR), DLS, NTA, and TEM, WB | CD63 | smoking triggers distinct biochemical alterations in salivary exosomes, uncovering a unique set of non-invasive biomarkers that may facilitate early oral cancer detection and prevention in high-risk individuals by exposing molecular changes prior to clinical manifestation. |
| (Li, Liu et al. 2025) | NA | NK-92MI cell line | cell culture supernatant | UC | WB, RNA RT-qPCR | NA | This groundbreaking study is the first to decode the heterogeneity of sEVs in head and neck squamous cell carcinoma (HNSCC) at single-vesicle resolution, identifying unique RNA signatures from different cell types. A key discovery is the role of T cell-derived sEVs in modulating NK cell function, shedding light on tumour-immune interactions. These insights enable more accurate in vitro models and open new avenues for clinical research on sEVs in HNSCC progression and therapy. |
| (Toh, Leong et al. 2024) | NA | Patient-derived cell line, HUVEC, NCCL247 | cell culture supernatant | UC, UF | NTA, SEM, WB | EGFR, EPCAM, cd9 cd54, GM130, GAPDH, CD9+CD63+CD81+ | The identification of the exosomal/EV-associated EGFR IsoD splice variant introduces a promising co-therapeutic approach to enhance tyrosine kinase inhibitor (TKI) efficacy, particularly in wild-type EGFR-overexpressing cancers. As a naturally occurring but understudied isoform, EGFR IsoD represents a potential modulator of drug response—and one of many functionally relevant EV-packaged splice variants yet to be discovered. This breakthrough not only advances the targeting of EGFR-driven cancers but also paves the way for uncovering similar therapeutically exploitable variants across other receptor tyrosine kinases (RTKs), offering new strategies to overcome kinase inhibitor resistance in refractory malignancies. |
| (Inyang, Evans et al. 2022) | NA | mEERL | cell culture supernatant | UF, Total Exosome Isolation reagent | NTA, WB | CD9 and TSG101 | Targeting TRPV1+ neurons (via QX-314 or TRPV1 antagonists) and inhibiting small extracellular vesicle (sEV) release from cancer cells emerge as promising therapeutic approaches. Disrupting eukaryotic initiation factor (eIF)-dependent translation pathways may hold promise for suppressing tumour progression. Additionally, AMPK activators like NCLS demonstrate dual benefits: inhibiting metastasis and tumour growth while exerting known analgesic effects, offering a multifaceted strategy for cancer therapy and pain management. |

**Acronyms:**

HNC: Head and neck cancer

HNSCC: Head and neck squamous cell carcinoma

OSCC: oral squamous cell carcinoma

TEM: transmission electron microscope

WB: Western blot

FM: Fluorescent microscopy

FCM: Flow cytometry

TRPS: Tenable resistive pulse sensing

PSD: Particle Size Distribution.

IHC: Immunohistochemistry staining

AFM: Atomic Force Microscope

MS: Mass Spectroscopy

OPSCC: Oropharyngeal squamous cell carcinoma

GORD: Gastroesophageal reflux disease

AML: Acute myeloid leukaemia

NA: Not applicable

**Supplementary Table 2**: Quality assessment of the 237 included manuscripts

| **Reference** | **QA tool** | Score | Grading |
| --- | --- | --- | --- |
| Ogawa 2023 | JBI CRITICAL APPRAISAL CHECKLIST FOR  ANALYTICAL CROSS SECTIONAL STUDIES | 6 out of 8 | Good |
| Tengler 2024 | JBI CRITICAL APPRAISAL CHECKLIST FOR  CASE CONTROL STUDIES | 9 out of 10 | Good |
| Cela 2023 | JBI CRITICAL APPRAISAL CHECKLIST FOR  ANALYTICAL CROSS SECTIONAL STUDIES | 6 out of 8 | Good |
| Seki 2023 | JBI CRITICAL APPRAISAL CHECKLIST FOR  ANALYTICAL CROSS SECTIONAL STUDIES | 7 out of 8 | Good |
| Sun 2024 | JBI CRITICAL APPRAISAL CHECKLIST FOR  ANALYTICAL CROSS SECTIONAL STUDIES | 6 out of 8 | Good |
| Kang 2024 | JBI CRITICAL APPRAISAL CHECKLIST FOR  ANALYTICAL CROSS SECTIONAL STUDIES | 6 out of 8 | Good |
| Wang 2023 | JBI CRITICAL APPRAISAL CHECKLIST FOR  ANALYTICAL CROSS SECTIONAL STUDIES | 5 out of 8 | Fair |
| Hofmann 2023 | JBI CRITICAL APPRAISAL CHECKLIST FOR  CASE CONTROL STUDIES | 6 out of 10 | Fair |
| Ludwigs 2023 | JBI CRITICAL APPRAISAL CHECKLIST FOR  CASE CONTROL STUDIES | 9 out of 10 | Good |
| You 2023 | JBI CRITICAL APPRAISAL CHECKLIST FOR  ANALYTICAL CROSS SECTIONAL STUDIES | 6 out of 8 | Good |
| AFFOLTER 2023 | JBI CRITICAL APPRAISAL CHECKLIST FOR  ANALYTICAL CROSS SECTIONAL STUDIES | 6 out of 8 | Good |
| Liu 2023 | JBI CRITICAL APPRAISAL CHECKLIST FOR  ANALYTICAL CROSS SECTIONAL STUDIES | 6 out of 8 | Good |
| Ye 2023 | JBI CRITICAL APPRAISAL CHECKLIST FOR  ANALYTICAL CROSS SECTIONAL STUDIES | 6 out of 8 | Good |
| Hill 2023 | JBI CRITICAL APPRAISAL CHECKLIST FOR  ANALYTICAL CROSS SECTIONAL STUDIES | 4 out of 8 | Fair |
| He 2023 | JBI CRITICAL APPRAISAL CHECKLIST FOR  ANALYTICAL CROSS SECTIONAL STUDIES | 7 out of 8 | Good |
| Zhang 2023 | JBI CRITICAL APPRAISAL CHECKLIST FOR  ANALYTICAL CROSS SECTIONAL STUDIES | 6 out of 8 | Good |
| You2023 | JBI CRITICAL APPRAISAL CHECKLIST FOR  ANALYTICAL CROSS SECTIONAL STUDIES | 6 out of 8 | Good |
| Tengler 2023 | JBI CRITICAL APPRAISAL CHECKLIST FOR  CASE CONTROL STUDIES | 10 out of 10 | Good |
| Feng2023 | JBI CRITICAL APPRAISAL CHECKLIST FOR  CASE CONTROL STUDIES | 6 out of 10 | Fair |
| Li 2023 | JBI CRITICAL APPRAISAL CHECKLIST FOR  ANALYTICAL CROSS SECTIONAL STUDIES | 6 out of 8 | Good |
| Callejo 2023 | JBI CRITICAL APPRAISAL CHECKLIST FOR  ANALYTICAL CROSS SECTIONAL STUDIES | 6 out of 8 | Good |
| Zhu 2023 | JBI CRITICAL APPRAISAL CHECKLIST FOR  ANALYTICAL CROSS SECTIONAL STUDIES | 8 out of 8 | Good |
| Theodoraki 2023 | JBI CRITICAL APPRAISAL CHECKLIST FOR  CASE CONTROL STUDIES | 6 out of 10 | Fair |
| Xiao 2022 | JBI CRITICAL APPRAISAL CHECKLIST FOR  CASE CONTROL STUDIES | 8 out of 10 | Good |
| Ludwig 2023 | JBI CRITICAL APPRAISAL CHECKLIST FOR  CASE CONTROL STUDIES | 9 out of 10 | Good |
| Faur 2023 | JBI CRITICAL APPRAISAL CHECKLIST FOR  CASE CONTROL STUDIES | 10 out of 10 | Good |
| Sun 2022 | JBI CRITICAL APPRAISAL CHECKLIST FOR  CASE CONTROL STUDIES | 6 out of 10 | Fair |
| Vasiljevic 2023 | JBI CRITICAL APPRAISAL CHECKLIST FOR  ANALYTICAL CROSS SECTIONAL STUDIES | 8 out of 8 | Good |
| Jha 2023 | JBI CRITICAL APPRAISAL CHECKLIST FOR  CASE CONTROL STUDIES | 6 out of 10 | Fair |
| Huang 2023 | JBI CRITICAL APPRAISAL CHECKLIST FOR  ANALYTICAL CROSS SECTIONAL STUDIES | 8 out of 8 | Good |
| Xu 2023 | JBI CRITICAL APPRAISAL CHECKLIST FOR  ANALYTICAL CROSS SECTIONAL STUDIES | 6 out of 8 | Good |
| Bozyk 2023 | JBI CRITICAL APPRAISAL CHECKLIST FOR  CASE CONTROL STUDIES | 10 out of 10 | Good |
| Wei 2024 | JBI CRITICAL APPRAISAL CHECKLIST FOR COHORT STUDIES | 8 out of 11 | Good |
| Han 2023 | JBI CRITICAL APPRAISAL CHECKLIST FOR  ANALYTICAL CROSS SECTIONAL STUDIES | 6 out of 8 | Good |
| Li 2023 | JBI CRITICAL APPRAISAL CHECKLIST FOR  ANALYTICAL CROSS SECTIONAL STUDIES | 6 out of 8 | Good |
| Zorrilla 2023 | JBI Critical Appraisal Checklist for Cohort Studies | 6 out of 11 | fair |
| Juvkam 2023 | JBI CRITICAL APPRAISAL CHECKLIST FOR  ANALYTICAL CROSS SECTIONAL STUDIES | 8 out of 8 | Good |
| Chen 2023 | JBI CRITICAL APPRAISAL CHECKLIST FOR  ANALYTICAL CROSS SECTIONAL STUDIES | 6 out of 8 | Good |
| Galvaro Jr 2023 | JBI CRITICAL APPRAISAL CHECKLIST FOR  ANALYTICAL CROSS SECTIONAL STUDIES | 6 out of 8 | Good |
| Bano 2023 | JBI CRITICAL APPRAISAL CHECKLIST FOR  CASE CONTROL STUDIES | 6 out of 10 | fair |
| Ludwigs 2022 | JBI CRITICAL APPRAISAL CHECKLIST FOR  ANALYTICAL CROSS SECTIONAL STUDIES | 8 out of 8 | Good |
| Li 2022 | JBI CRITICAL APPRAISAL CHECKLIST FOR  CASE CONTROL STUDIES | 9 out of 10 | Good |
| Wu 2022 | JBI CRITICAL APPRAISAL CHECKLIST FOR  ANALYTICAL CROSS SECTIONAL STUDIES | 8 out of 8 | Good |
| Zhuang 2022 | JBI CRITICAL APPRAISAL CHECKLIST FOR  ANALYTICAL CROSS SECTIONAL STUDIES | 8 out of 8 | Good |
| Hofmann 2022 | JBI CRITICAL APPRAISAL CHECKLIST FOR  ANALYTICAL CROSS SECTIONAL STUDIES | 8 out of 8 | Good |
| Deng 2022 | JBI CRITICAL APPRAISAL CHECKLIST FOR  ANALYTICAL CROSS SECTIONAL STUDIES | 8 out of 8 | Good |
| Li 2022 | JBI CRITICAL APPRAISAL CHECKLIST FOR  CASE CONTROL STUDIES | 5 out 10 | Fair |
| Mayne 2020 | JBI CRITICAL APPRAISAL CHECKLIST FOR  CASE CONTROL STUDIES | 8 out 10 | Good |
| Chen 2022 | JBI CRITICAL APPRAISAL CHECKLIST FOR  ANALYTICAL CROSS SECTIONAL STUDIES | 8 out of 8 | Good |
| Wang 2021 | JBI CRITICAL APPRAISAL CHECKLIST FOR  ANALYTICAL CROSS SECTIONAL STUDIES | 6 out of 8 | Good |
| Smolarz 2023 | JBI CRITICAL APPRAISAL CHECKLIST FOR  ANALYTICAL CROSS SECTIONAL STUDIES | 8 out of 8 | Good |
| Bai 2021 | JBI CRITICAL APPRAISAL CHECKLIST FOR  ANALYTICAL CROSS SECTIONAL STUDIES | 8 out of 8 | Good |
| Wang 2022 | JBI CRITICAL APPRAISAL CHECKLIST FOR  ANALYTICAL CROSS SECTIONAL STUDIES | 8 out of 8 | Good |
| Wu 2022 | JBI CRITICAL APPRAISAL CHECKLIST FOR  CASE CONTROL STUDIES | 10 out of 10 | Good |
| Yamana 2023 | JBI CRITICAL APPRAISAL CHECKLIST FOR  ANALYTICAL CROSS SECTIONAL STUDIES | 7 out of 8 | Good |
| Yuan 2022 | JBI CRITICAL APPRAISAL CHECKLIST FOR  ANALYTICAL CROSS SECTIONAL STUDIES | 7 out of 8 | Good |
| Xu 2022 | JBI CRITICAL APPRAISAL CHECKLIST FOR  ANALYTICAL CROSS SECTIONAL STUDIES | 8 out of 8 | Good |
| Yang 2022 | JBI CRITICAL APPRAISAL CHECKLIST FOR  CASE CONTROL STUDIES | 8 out of 10 | Good |
| Lou 2022 | JBI CRITICAL APPRAISAL CHECKLIST FOR  ANALYTICAL CROSS SECTIONAL STUDIES | 7 out of 8 | Good |
| Li 2022 | JBI CRITICAL APPRAISAL CHECKLIST FOR  ANALYTICAL CROSS SECTIONAL STUDIES | 8 out of 8 | Good |
| Shafiaa 2022 | JBI CRITICAL APPRAISAL CHECKLIST FOR  ANALYTICAL CROSS SECTIONAL STUDIES | 5 out of 8 | Fair |
| Hong 2022 | JBI CRITICAL APPRAISAL CHECKLIST FOR  ANALYTICAL CROSS SECTIONAL STUDIES | 6 out of 8 | Good |
| Wang 2021 | JBI CRITICAL APPRAISAL CHECKLIST FOR  ANALYTICAL CROSS SECTIONAL STUDIES | 8 out of 8 | Good |
| Jiang 2022 | JBI CRITICAL APPRAISAL CHECKLIST FOR  ANALYTICAL CROSS SECTIONAL STUDIES | 8 out of 8 | Good |
| Benecke 2022 | JBI CRITICAL APPRAISAL CHECKLIST FOR  CASE CONTROL STUDIES | 7 out of 10 | Fair |
| Faur 2022 | JBI CRITICAL APPRAISAL CHECKLIST FOR  CASE CONTROL STUDIES | 8 out of 10 | Good |
| Liu 2022 | JBI CRITICAL APPRAISAL CHECKLIST FOR  ANALYTICAL CROSS SECTIONAL STUDIES | 8 out of 8 | Good |
| Liu 2022 | JBI CRITICAL APPRAISAL CHECKLIST FOR  ANALYTICAL CROSS SECTIONAL STUDIES | 8 out of 8 | Good |
| Yang 2021 | JBI CRITICAL APPRAISAL CHECKLIST FOR  ANALYTICAL CROSS SECTIONAL STUDIES | 8 out of 8 | Good |
| Wang 2022 | JBI CRITICAL APPRAISAL CHECKLIST FOR  ANALYTICAL CROSS SECTIONAL STUDIES | 7 out of 8 | Good |
| Huang 2021 | JBI CRITICAL APPRAISAL CHECKLIST FOR  ANALYTICAL CROSS SECTIONAL STUDIES | 7 out of 8 | Good |
| Silva 2021 | JBI CRITICAL APPRAISAL CHECKLIST FOR  ANALYTICAL CROSS SECTIONAL STUDIES | 8 out of 8 | Good |
| Yang 2021 | JBI CRITICAL APPRAISAL CHECKLIST FOR  ANALYTICAL CROSS SECTIONAL STUDIES | 8 out of 8 | Good |
| Goudsmit2021 | JBI CRITICAL APPRAISAL CHECKLIST FOR  ANALYTICAL CROSS SECTIONAL STUDIES | 6 out of 8 | Good |
| Gluszko 2021 | JBI CRITICAL APPRAISAL CHECKLIST FOR  ANALYTICAL CROSS SECTIONAL STUDIES | 6 out of 8 | Good |
| Liu 2021 | JBI CRITICAL APPRAISAL CHECKLIST FOR  ANALYTICAL CROSS SECTIONAL STUDIES | 3 out of 8 | Poor |
| Tang 2021 | JBI CRITICAL APPRAISAL CHECKLIST FOR  CASE CONTROL STUDIES | 8 out of 10 | Good |
| Han 2021 | JBI CRITICAL APPRAISAL CHECKLIST FOR  ANALYTICAL CROSS SECTIONAL STUDIES | 7 out of 8 | Good |
| Lu 2021 | JBI CRITICAL APPRAISAL CHECKLIST FOR  ANALYTICAL CROSS SECTIONAL STUDIES | 7 out of 8 | Good |
| Yan 2021 | JBI CRITICAL APPRAISAL CHECKLIST FOR  ANALYTICAL CROSS SECTIONAL STUDIES | 8 out of 8 | Good |
| Ai 2021 | JBI CRITICAL APPRAISAL CHECKLIST FOR  ANALYTICAL CROSS SECTIONAL STUDIES | 3 out of 8 | Poor |
| Zhou 2021 | JBI CRITICAL APPRAISAL CHECKLIST FOR  ANALYTICAL CROSS SECTIONAL STUDIES | 5 out of 8/6 | Fair |
| Zhu 2021 | JBI CRITICAL APPRAISAL CHECKLIST FOR  ANALYTICAL CROSS SECTIONAL STUDIES | 8 out of 8 | Good |
| Busso-Lopes 2021 | JBI CRITICAL APPRAISAL CHECKLIST FOR  ANALYTICAL CROSS SECTIONAL STUDIES | 8 out of 8/5 | Good |
| Qu 2021 | JBI CRITICAL APPRAISAL CHECKLIST FOR  CASE CONTROL STUDIES | 10 out of 10 | Good |
| Bottino 2021 | JBI CRITICAL APPRAISAL CHECKLIST FOR  ANALYTICAL CROSS SECTIONAL STUDIES | 7 out of 8 | Good |
| Gong 2021 | JBI CRITICAL APPRAISAL CHECKLIST FOR  ANALYTICAL CROSS SECTIONAL STUDIES | 6 out of 8 | Good |
| Cohen 2021 | JBI CRITICAL APPRAISAL CHECKLIST FOR  ANALYTICAL CROSS SECTIONAL STUDIES | 8 out of 8 | Good |
| Pang 2021 | JBI CRITICAL APPRAISAL CHECKLIST FOR  ANALYTICAL CROSS SECTIONAL STUDIES | 8 out of 8 | Good |
| Nakamichi 2020 | JBI CRITICAL APPRAISAL CHECKLIST FOR  CASE CONTROL STUDIES | 8 out of 10 | Good |
| Li 2021 | JBI CRITICAL APPRAISAL CHECKLIST FOR  ANALYTICAL CROSS SECTIONAL STUDIES | 8 out of 8 | Good |
| Guo 2021 | JBI CRITICAL APPRAISAL CHECKLIST FOR  ANALYTICAL CROSS SECTIONAL STUDIES | 5 out of 8 | Fair |
| Guo 2021 | JBI CRITICAL APPRAISAL CHECKLIST FOR  CASE CONTROL STUDIES | 6 out of 10 | Fair |
| He 2021 | JBI CRITICAL APPRAISAL CHECKLIST FOR  ANALYTICAL CROSS SECTIONAL STUDIES | 8 out of 8 | Good |
| He 2021 | JBI CRITICAL APPRAISAL CHECKLIST FOR  CASE CONTROL STUDIES | 9 out of 10 | Good |
| He 2021 | JBI CRITICAL APPRAISAL CHECKLIST FOR  ANALYTICAL CROSS SECTIONAL STUDIES | 7 out of 8 | Good |
| lee 2021 | JBI CRITICAL APPRAISAL CHECKLIST FOR  ANALYTICAL CROSS SECTIONAL STUDIES | 6 out of 8 | Good |
| Zheng 2020 | JBI CRITICAL APPRAISAL CHECKLIST FOR  ANALYTICAL CROSS SECTIONAL STUDIES | 7 out of 8 | Good |
| Guerreiro 2020 | JBI CRITICAL APPRAISAL CHECKLIST FOR  ANALYTICAL CROSS SECTIONAL STUDIES | 8 out of 8 | Good |
| Luo 2020 | JBI CRITICAL APPRAISAL CHECKLIST FOR  CASE CONTROL STUDIES | 6 out of 10 | Fair |
| Wang 2020 | JBI CRITICAL APPRAISAL CHECKLIST FOR  ANALYTICAL CROSS SECTIONAL STUDIES | 8 out of 8 | Good |
| Beccard 2020 | JBI CRITICAL APPRAISAL CHECKLIST FOR  ANALYTICAL CROSS SECTIONAL STUDIES | 8 out of 8 | Good |
| Jin 2021 | JBI CRITICAL APPRAISAL CHECKLIST FOR  ANALYTICAL CROSS SECTIONAL STUDIES | 6 out of 8 | Good |
| Higaki 2020 | JBI CRITICAL APPRAISAL CHECKLIST FOR  ANALYTICAL CROSS SECTIONAL STUDIES | 7 out of 8 | Good |
| Amit 2020 | JBI CRITICAL APPRAISAL CHECKLIST FOR  ANALYTICAL CROSS SECTIONAL STUDIES | 7 out of 8 | Good |
| Tong 2020 | JBI CRITICAL APPRAISAL CHECKLIST FOR  ANALYTICAL CROSS SECTIONAL STUDIES | 8 out of 8 | Good |
| He 2020 | JBI CRITICAL APPRAISAL CHECKLIST FOR  CASE CONTROL STUDIES | 10 out of 10 | Good |
| Abramowicz 2020 | JBI CRITICAL APPRAISAL CHECKLIST FOR  ANALYTICAL CROSS SECTIONAL STUDIES | 8 out of 8 | Good |
| Ono 2020 | JBI CRITICAL APPRAISAL CHECKLIST FOR  ANALYTICAL CROSS SECTIONAL STUDIES | 7 out of 8 | Good |
| Kulkarni 2020 | JBI CRITICAL APPRAISAL CHECKLIST FOR  ANALYTICAL CROSS SECTIONAL STUDIES | 5 out of 8 | Good |
| Quin 2020 | JBI CRITICAL APPRAISAL CHECKLIST FOR  ANALYTICAL CROSS SECTIONAL STUDIES | 8 out of 8 | Good |
| Li 2020 | JBI CRITICAL APPRAISAL CHECKLIST FOR  ANALYTICAL CROSS SECTIONAL STUDIES | 6 out of 8 | Good |
| Zhang 2020 | JBI CRITICAL APPRAISAL CHECKLIST FOR  ANALYTICAL CROSS SECTIONAL STUDIES | 8 out of 8 | Good |
| Shoff 2020 | JBI CRITICAL APPRAISAL CHECKLIST FOR  ANALYTICAL CROSS SECTIONAL STUDIES | 4 out of 8 | Poor |
| Quin 2020 | JBI CRITICAL APPRAISAL CHECKLIST FOR  ANALYTICAL CROSS SECTIONAL STUDIES | 6 out of 8 | Good |
| Ludwig 2020 | JBI CRITICAL APPRAISAL CHECKLIST FOR  ANALYTICAL CROSS SECTIONAL STUDIES | 8 out of 8 | Good |
| Sanada 2019 | JBI CRITICAL APPRAISAL CHECKLIST FOR  CASE CONTROL STUDIES | 7 out of 10 | Good |
| Ludwig 2020 | JBI CRITICAL APPRAISAL CHECKLIST FOR  CASE CONTROL STUDIES | 10 out of 10 | Good |
| Jin 2020 | JBI CRITICAL APPRAISAL CHECKLIST FOR  ANALYTICAL CROSS SECTIONAL STUDIES | 8 out of 8 | Good |
| Zhu 2020 | JBI CRITICAL APPRAISAL CHECKLIST FOR  ANALYTICAL CROSS SECTIONAL STUDIES | 8 out of 8 | Good |
| Tomita 2020 | JBI CRITICAL APPRAISAL CHECKLIST FOR  ANALYTICAL CROSS SECTIONAL STUDIES | 8 out of 8 | Good |
| Rasso 2020 | JBI CRITICAL APPRAISAL CHECKLIST FOR  ANALYTICAL CROSS SECTIONAL STUDIES | 8 out of 8 | Good |
| Wang 2020 | JBI CRITICAL APPRAISAL CHECKLIST FOR  CASE CONTROL STUDIES | 7 out of 10 | Good |
| Sato 2019 | JBI CRITICAL APPRAISAL CHECKLIST FOR  ANALYTICAL CROSS SECTIONAL STUDIES | 6 out of 8 | Good |
| Li 2019 | JBI CRITICAL APPRAISAL CHECKLIST FOR  CASE CONTROL STUDIES | 10 out of 10 | Good |
| Xie 2019 | JBI CRITICAL APPRAISAL CHECKLIST FOR  ANALYTICAL CROSS SECTIONAL STUDIES | 7 out of 8 | Good |
| Li 2019 | JBI CRITICAL APPRAISAL CHECKLIST FOR  CASE CONTROL STUDIES | 7 out of 10 | Good |
| Wang 2019 | JBI CRITICAL APPRAISAL CHECKLIST FOR  ANALYTICAL CROSS SECTIONAL STUDIES | 7 out of 8 | Good |
| Ji 2019 | JBI CRITICAL APPRAISAL CHECKLIST FOR  ANALYTICAL CROSS SECTIONAL STUDIES | 8 out of 8 | Good |
| Junior 2019 | JBI CRITICAL APPRAISAL CHECKLIST FOR  ANALYTICAL CROSS SECTIONAL STUDIES | 6 out of 8 | Good |
| Quin 2019 | JBI CRITICAL APPRAISAL CHECKLIST FOR  ANALYTICAL CROSS SECTIONAL STUDIES | 8 out of 8 | Good |
| Dourado 2019 | JBI CRITICAL APPRAISAL CHECKLIST FOR  ANALYTICAL CROSS SECTIONAL STUDIES | 7 out of 8 | Good |
| Jiang 2019 | JBI CRITICAL APPRAISAL CHECKLIST FOR  ANALYTICAL CROSS SECTIONAL STUDIES | 6 out of 8 | Good |
| Li 2019 | JBI CRITICAL APPRAISAL CHECKLIST FOR  ANALYTICAL CROSS SECTIONAL STUDIES | 7 out of 8 | Good |
| Zhong 2019 | JBI CRITICAL APPRAISAL CHECKLIST FOR  CASE CONTROL STUDIES | 8 out of 10 | Good |
| Li 2019 | JBI CRITICAL APPRAISAL CHECKLIST FOR  ANALYTICAL CROSS SECTIONAL STUDIES | 6 out of 8 | Good |
| Wang 2019 | JBI CRITICAL APPRAISAL CHECKLIST FOR  ANALYTICAL CROSS SECTIONAL STUDIES | 6 out of 8 | Good |
| Wang 2019 | JBI CRITICAL APPRAISAL CHECKLIST FOR  ANALYTICAL CROSS SECTIONAL STUDIES | 6 out of 8 | Good |
| Wang 2019 | JBI CRITICAL APPRAISAL CHECKLIST FOR  ANALYTICAL CROSS SECTIONAL STUDIES | 8 out of 8 | Good |
| Cai 2019 | JBI CRITICAL APPRAISAL CHECKLIST FOR  ANALYTICAL CROSS SECTIONAL STUDIES | 6 out of 8 | Good |
| San 2019 | JBI CRITICAL APPRAISAL CHECKLIST FOR  ANALYTICAL CROSS SECTIONAL STUDIES | 4 out of 8 | Fair |
| Ono 2018 | JBI CRITICAL APPRAISAL CHECKLIST FOR  ANALYTICAL CROSS SECTIONAL STUDIES | 8 out of 8 | Good |
| Chen 2018 | JBI CRITICAL APPRAISAL CHECKLIST FOR  ANALYTICAL CROSS SECTIONAL STUDIES | 8 out of 8 | Good |
| Fujiwara 2018 | JBI CRITICAL APPRAISAL CHECKLIST FOR  ANALYTICAL CROSS SECTIONAL STUDIES | 7 out of 8 | Good |
| Guerreiro 2018 | JBI CRITICAL APPRAISAL CHECKLIST FOR  ANALYTICAL CROSS SECTIONAL STUDIES | 7 out of 8 | Good |
| Xiao 2018 | JBI CRITICAL APPRAISAL CHECKLIST FOR  ANALYTICAL CROSS SECTIONAL STUDIES | 7 out of 8 | Good |
| Qu 2021 | JBI CRITICAL APPRAISAL CHECKLIST FOR  CASE CONTROL STUDIES | 9 out of 10 | Good |
| Theodoraki 2018 | JBI CRITICAL APPRAISAL CHECKLIST FOR  CASE CONTROL STUDIES | 8 out of 10 | Good |
| Tamkovicha 2018 | JBI CRITICAL APPRAISAL CHECKLIST FOR  CASE CONTROL STUDIES | 9 out of 10 | Good |
| Theodoraki 2018 | JBI CRITICAL APPRAISAL CHECKLIST FOR  ANALYTICAL CROSS SECTIONAL STUDIES | 8 out of 8 | Good |
| wang 2018 | JBI CRITICAL APPRAISAL CHECKLIST FOR  ANALYTICAL CROSS SECTIONAL STUDIES | 8 out of 8 | Good |
| Qadir 2018 | JBI CRITICAL APPRAISAL CHECKLIST FOR  ANALYTICAL CROSS SECTIONAL STUDIES | 7 out of 8 | Good |
| Principe 2018 | JBI CRITICAL APPRAISAL CHECKLIST FOR  ANALYTICAL CROSS SECTIONAL STUDIES | 6 out of 8 | Good |
| momen-heravi 2018 | JBI CRITICAL APPRAISAL CHECKLIST FOR  CASE CONTROL STUDIES | 10 out of 10 | Good |
| Li 2018 | JBI CRITICAL APPRAISAL CHECKLIST FOR  ANALYTICAL CROSS SECTIONAL STUDIES | 7 out of 8 | Good |
| Andrade 2017 | JBI CRITICAL APPRAISAL CHECKLIST FOR  ANALYTICAL CROSS SECTIONAL STUDIES | 6 out of 8 | Good |
| Langevin 2017 | JBI CRITICAL APPRAISAL CHECKLIST FOR  ANALYTICAL CROSS SECTIONAL STUDIES | 7 out of 8 | Good |
| Liu 2017 | JBI CRITICAL APPRAISAL CHECKLIST FOR  ANALYTICAL CROSS SECTIONAL STUDIES | 6 out of 8 | Good |
| Al samadi 2017 | JBI CRITICAL APPRAISAL CHECKLIST FOR  ANALYTICAL CROSS SECTIONAL STUDIES | 6 out of 8 | Good |
| Overmiller 2017 | JBI CRITICAL APPRAISAL CHECKLIST FOR  ANALYTICAL CROSS SECTIONAL STUDIES | 5 out of 8 | Good |
| Menck 2018 | JBI CRITICAL APPRAISAL CHECKLIST FOR  CASE CONTROL STUDIES | 8 out of 10 | Good |
| Huaitong 2017 | JBI CRITICAL APPRAISAL CHECKLIST FOR  ANALYTICAL CROSS SECTIONAL STUDIES | 5 out of 8 | Fair |
| Maybruck 2017 | JBI CRITICAL APPRAISAL CHECKLIST FOR  ANALYTICAL CROSS SECTIONAL STUDIES | 4 out of 8 | Fair |
| Yunusova 2017 | JBI CRITICAL APPRAISAL CHECKLIST FOR  ANALYTICAL CROSS SECTIONAL STUDIES | 4 out of 8 | Fair |
| Rabinowits 2017 | JBI CRITICAL APPRAISAL CHECKLIST FOR  ANALYTICAL CROSS SECTIONAL STUDIES | 6 out of 8 | Good |
| Yuana 2017 | JBI CRITICAL APPRAISAL CHECKLIST FOR  ANALYTICAL CROSS SECTIONAL STUDIES | 7 out of 8 | Good |
| Mutschelknaus 2017 | JBI CRITICAL APPRAISAL CHECKLIST FOR  ANALYTICAL CROSS SECTIONAL STUDIES | 7 out of 8 | Good |
| Li 2016 | JBI CRITICAL APPRAISAL CHECKLIST FOR  ANALYTICAL CROSS SECTIONAL STUDIES | 6 out of 8 | Good |
| Sakha 2016 | JBI CRITICAL APPRAISAL CHECKLIST FOR  ANALYTICAL CROSS SECTIONAL STUDIES | 7 out of 8 | Good |
| Languino 2016 | JBI CRITICAL APPRAISAL CHECKLIST FOR  ANALYTICAL CROSS SECTIONAL STUDIES | 6 out of 8 | Good |
| Vered 2015 | JBI CRITICAL APPRAISAL CHECKLIST FOR  ANALYTICAL CROSS SECTIONAL STUDIES | 7 out of 8 | Good |
| Winck 2015 | JBI CRITICAL APPRAISAL CHECKLIST FOR  CASE CONTROL STUDIES | 7 out of 10 | Fair |
| Kim 2005 | JBI CRITICAL APPRAISAL CHECKLIST FOR  ANALYTICAL CROSS SECTIONAL STUDIES | 5 out of 8 | Fair |
| Hong 2016 | JBI CRITICAL APPRAISAL CHECKLIST FOR  ANALYTICAL CROSS SECTIONAL STUDIES | 6 out of 8 | Good |
| Man 2022 | JBI CRITICAL APPRAISAL CHECKLIST FOR  CASE CONTROL STUDIES | 9 out of 10 | Good |
| Flemming 2020 | JBI CRITICAL APPRAISAL CHECKLIST FOR  ANALYTICAL CROSS SECTIONAL STUDIES | 6 out of 8 | Good |
| Bernareggi 2022 | JBI CRITICAL APPRAISAL CHECKLIST FOR  ANALYTICAL CROSS SECTIONAL STUDIES | 8 out of 8 | Good |
| Jelonek 2015 | JBI CRITICAL APPRAISAL CHECKLIST FOR  ANALYTICAL CROSS SECTIONAL STUDIES | 7 out of 8 | Good |
| Dong 2022 | JBI CRITICAL APPRAISAL CHECKLIST FOR  ANALYTICAL CROSS SECTIONAL STUDIES | 7 out of 8 | Good |
| Yuan 2021 | JBI CRITICAL APPRAISAL CHECKLIST FOR  ANALYTICAL CROSS SECTIONAL STUDIES | 7 out of 8 | Good |
| Man 2022 | JBI CRITICAL APPRAISAL CHECKLIST FOR  CASE CONTROL STUDIES | 8 out of 10 | Good |
| Omori 2024 | JBI CRITICAL APPRAISAL CHECKLIST FOR  ANALYTICAL CROSS SECTIONAL STUDIES | 6 out of 8 | Good |
| Miao 2024 | JBI CRITICAL APPRAISAL CHECKLIST FOR  ANALYTICAL CROSS SECTIONAL STUDIES | 6 out of 8 | good |
| Apeltrath 2024 | JBI CRITICAL APPRAISAL CHECKLIST FOR COHORT STUDIES | 7 out of 11 | Fair |
| LI 2024 | JBI CRITICAL APPRAISAL CHECKLIST FOR  CASE CONTROL STUDIES | 7 out of 10 | Fair |
| Huang 2024 | JBI CRITICAL APPRAISAL CHECKLIST FOR  ANALYTICAL CROSS SECTIONAL STUDIES | 6 out of 8 | Good |
| yu 2024 | JBI CRITICAL APPRAISAL CHECKLIST FOR COHORT STUDIES | 7 out of 11 | fair |
| Bugajova 2024 | JBI CRITICAL APPRAISAL CHECKLIST FOR COHORT STUDIES | 7 out of 11 | fair |
| Xie 2024 | JBI CRITICAL APPRAISAL CHECKLIST FOR  CASE CONTROL STUDIES | 7 out of 10 | fair |
| Sato 2024 | JBI CRITICAL APPRAISAL CHECKLIST FOR COHORT STUDIES | 7 out of 11 | fair |
| Pande | JBI CRITICAL APPRAISAL CHECKLIST FOR  ANALYTICAL CROSS SECTIONAL STUDIES | 6 out of 8 | Good |
| Liu 2024 | JBI CRITICAL APPRAISAL CHECKLIST FOR  ANALYTICAL CROSS SECTIONAL STUDIES | 5 out of 8 | fair |
| Theodoraki 2024 | JBI CRITICAL APPRAISAL CHECKLIST FOR  CASE CONTROL STUDIES | 6 out of 10 | fair |
| Yang 2024 | JBI CRITICAL APPRAISAL CHECKLIST FOR  ANALYTICAL CROSS SECTIONAL STUDIES | 7 out of 8 | Good |
| Qiaoshi Xu 24 | JBI CRITICAL APPRAISAL CHECKLIST FOR  ANALYTICAL CROSS SECTIONAL STUDIES | 6 out of 8 | Good |
| Rubenich 2024 | JBI CRITICAL APPRAISAL CHECKLIST FOR  ANALYTICAL CROSS SECTIONAL STUDIES | 7 out of 8 | Good |
| Ha 2024 | JBI CRITICAL APPRAISAL CHECKLIST FOR  ANALYTICAL CROSS SECTIONAL STUDIES | 5 out of 8 | Fair |
| Metsäniitty 2024 | JBI CRITICAL APPRAISAL CHECKLIST FOR  ANALYTICAL CROSS SECTIONAL STUDIES | 6 out of 8 | Good |
| Lv 2024 | JBI CRITICAL APPRAISAL CHECKLIST FOR  ANALYTICAL CROSS SECTIONAL STUDIES | 6 out of 8 | Good |
| Søland 2024 | JBI CRITICAL APPRAISAL CHECKLIST FOR  ANALYTICAL CROSS SECTIONAL STUDIES | 6 out of 8 | Good |
| Li 2024 | JBI CRITICAL APPRAISAL CHECKLIST FOR  ANALYTICAL CROSS SECTIONAL STUDIES | 6 out of 8 | Good |
| Inyang 2024 | JBI CRITICAL APPRAISAL CHECKLIST FOR  ANALYTICAL CROSS SECTIONAL STUDIES | 6 out of 8 | Good |
| Li 2024 | JBI CRITICAL APPRAISAL CHECKLIST FOR  ANALYTICAL CROSS SECTIONAL STUDIES | 7 out of 8 | Good |
| Li 2024 | JBI CRITICAL APPRAISAL CHECKLIST FOR  ANALYTICAL CROSS SECTIONAL STUDIES | 6 out of 8 | Good |
| Fu 2024 | JBI CRITICAL APPRAISAL CHECKLIST FOR COHORT STUDIES | 8 out of 11 | Good |
| Yoshida 2024 | JBI CRITICAL APPRAISAL CHECKLIST FOR  ANALYTICAL CROSS SECTIONAL STUDIES | 6 out of 8 | Good |
| Zhou 2024 | JBI CRITICAL APPRAISAL CHECKLIST FOR  ANALYTICAL CROSS SECTIONAL STUDIES | 5 out of 8 | fair |
| Metsäniitty 2024 | JBI CRITICAL APPRAISAL CHECKLIST FOR  ANALYTICAL CROSS SECTIONAL STUDIES | 5 out of 8 | fair |
| Yan 2024 | JBI CRITICAL APPRAISAL CHECKLIST FOR  ANALYTICAL CROSS SECTIONAL STUDIES | 6 out of 8 | Good |
| Liu 2024 | JBI CRITICAL APPRAISAL CHECKLIST FOR  ANALYTICAL CROSS SECTIONAL STUDIES | 6 out of 8 | Good |
| Ding 2024 | JBI CRITICAL APPRAISAL CHECKLIST FOR  ANALYTICAL CROSS SECTIONAL STUDIES | 7 out of 8 | Good |
| Toh 2024 | JBI CRITICAL APPRAISAL CHECKLIST FOR  ANALYTICAL CROSS SECTIONAL STUDIES | 6 out of 8 | Good |
| Prestel 2024 | JBI CRITICAL APPRAISAL CHECKLIST FOR  ANALYTICAL CROSS SECTIONAL STUDIES | 6 out of 8 | Good |
| Yadav 2024 | JBI CRITICAL APPRAISAL CHECKLIST FOR  ANALYTICAL CROSS SECTIONAL STUDIES | 6 OUT OF | Good |
| Zhou 2024 | JBI CRITICAL APPRAISAL CHECKLIST FOR  ANALYTICAL CROSS SECTIONAL STUDIES | 7 OUT OF 8 | Good |
| Seki 2024 | JBI CRITICAL APPRAISAL CHECKLIST FOR  CASE CONTROL STUDIES | 9 out of 10 | Good |
| Cheng 2024 | JBI CRITICAL APPRAISAL CHECKLIST FOR  ANALYTICAL CROSS SECTIONAL STUDIES | 6 out of 8 | Good |
| Wu 2024 | JBI CRITICAL APPRAISAL CHECKLIST FOR  ANALYTICAL CROSS SECTIONAL STUDIES | 7out of 8 | Good |
| Rizwan 2025 | JBI CRITICAL APPRAISAL CHECKLIST FOR COHORT STUDIES | 9 out of 11 | Good |
| Li 2024 | JBI CRITICAL APPRAISAL CHECKLIST FOR  ANALYTICAL CROSS SECTIONAL STUDIES | 6 out of 8 | Good |
| Yang 2024 | JBI CRITICAL APPRAISAL CHECKLIST FOR  ANALYTICAL CROSS SECTIONAL STUDIES | 6 out of 8 | Good |
| Madhu 2025 | JBI CRITICAL APPRAISAL CHECKLIST FOR  ANALYTICAL CROSS SECTIONAL STUDIES | 6 out of 8 | Good |
| Liu 2025 | JBI CRITICAL APPRAISAL CHECKLIST FOR  ANALYTICAL CROSS SECTIONAL STUDIES | 6 out of 8 | Good |
| Hu 2024 | JBI CRITICAL APPRAISAL CHECKLIST FOR  ANALYTICAL CROSS SECTIONAL STUDIES | 6 out of 8 | Good |
| Xia 2025 | JBI CRITICAL APPRAISAL CHECKLIST FOR  ANALYTICAL CROSS SECTIONAL STUDIES | 6 out of 8 | Good |
| Yadav 2025 | JBI CRITICAL APPRAISAL CHECKLIST FOR  ANALYTICAL CROSS SECTIONAL STUDIES | 7 out of 8 | Good |
| Wang 2025 | JBI CRITICAL APPRAISAL CHECKLIST FOR  ANALYTICAL CROSS SECTIONAL STUDIES | 8 out of 8 | Good |
| Ko 2024 | JBI CRITICAL APPRAISAL CHECKLIST FOR  ANALYTICAL CROSS SECTIONAL STUDIES | 6 out of 8 | Good |
| Chen 2025 | JBI CRITICAL APPRAISAL CHECKLIST FOR  CASE CONTROL STUDIES | 9 out of 10 | Good |
| Lipka 2025 | JBI CRITICAL APPRAISAL CHECKLIST FOR  ANALYTICAL CROSS SECTIONAL STUDIES | 6 out of 8 | Good |
| Zhao 2025 | JBI CRITICAL APPRAISAL CHECKLIST FOR  CASE CONTROL STUDIES | 9 out of 10 | Good |
| Wang 2025 | JBI CRITICAL APPRAISAL CHECKLIST FOR  ANALYTICAL CROSS SECTIONAL STUDIES | 6 out of 8 | Good |
| Li 2025 | JBI CRITICAL APPRAISAL CHECKLIST FOR  ANALYTICAL CROSS SECTIONAL STUDIES | 6 out of 8 | Good |
| Zhang 2025 | JBI CRITICAL APPRAISAL CHECKLIST FOR  ANALYTICAL CROSS SECTIONAL STUDIES | 6 out of 8 | Good |
| Bano 2025 | JBI CRITICAL APPRAISAL CHECKLIST FOR  CASE CONTROL STUDIES | 9 out of 10 | Good |
| Li 2024 | JBI CRITICAL APPRAISAL CHECKLIST FOR  ANALYTICAL CROSS SECTIONAL STUDIES | 6 out of 8 | Good |
| Inyang 2022 | JBI CRITICAL APPRAISAL CHECKLIST FOR  ANALYTICAL CROSS SECTIONAL STUDIES | 8 out of 8 | Good |

JBI - Joanna Briggs Institute, Grading for each tool was decided as follows:

JBI CRITICAL APPRAISAL CHECKLIST FOR ANALYTICAL CROSS SECTIONAL STUDIES 8-6 Good, 5-3 Fair and <3 Poor

JBI CRITICAL APPRAISAL CHECKLIST FOR CASE CONTROL STUDIES 8-10 good, 4-7 fair, and <4 poor and Cohort 9-11 good, 5-8 Fair and <5 Poor.

**References**

Abramowicz, A., et al. (2020). "MicroRNA Profile of Exosomes and Parental Cells is Differently Affected by Ionizing Radiation." Radiation Research **194**(2): 133-142.

Affolter, A., et al. (2023). "Modulation of PD-L1 expression by standard therapy in head and neck cancer cell lines and exosomes." INTERNATIONAL JOURNAL OF ONCOLOGY **63**(3).

Ai, Y. L., et al. (2021). "Exosomal LncRNA LBX1-AS1 Derived From RBPJ Overexpressed-Macrophages Inhibits Oral Squamous Cell Carcinoma Progress <i>via</i> miR-182-5p/FOXO3." FRONTIERS IN ONCOLOGY **11**.

Al-Samadi, A., et al. (2017). "Crosstalk between tongue carcinoma cells, extracellular vesicles, and immune cells in <i>in vitro</i> and <i>in vivo</i> models." ONCOTARGET **8**(36): 60123-60134.

Amit, M., et al. (2020). "Loss of p53 drives neuron reprogramming in head and neck cancer." NATURE **578**(7795): 449-+.

Apeltrath, C., et al. (2024). "Extracellular Vesicle microRNAs as Possible Liquid Biopsy Markers in HNSCC—A Longitudinal, Monocentric Study." Cancers **16**(22): 3793.

Bai, S. W., et al. (2022). "Exocyst controls exosome biogenesis via Rab11a." MOLECULAR THERAPY-NUCLEIC ACIDS **27**: 535-546.

Bano, A., et al. (2023). "Exploring salivary exosomes as early predictors of oral cancer in susceptible tobacco consumers: noninvasive diagnostic and prognostic applications." Journal of Cancer Research and Clinical Oncology **149**(17): 15781-15793.

Bano, A., et al. (2025). "Smoking-induced shifts in salivary exosomal cytokines and amino acid profiles as potential early biomarkers for oral cancer." Cytokine **187**: 156857.

Beccard, I. J., et al. (2020). "Immune Suppressive Effects of Plasma-Derived Exosome Populations in Head and Neck Cancer." CANCERS **12**(7).

Benecke, L., et al. (2022). "Isolation and analysis of tumor-derived extracellular vesicles from head and neck squamous cell carcinoma plasma by galectin-based glycan recognition particles." Int J Oncol **61**(5): 133.

Bernareggi, D., et al. (2022). "CHMP2A regulates tumor sensitivity to natural killer cell-mediated cytotoxicity." NATURE COMMUNICATIONS **13**(1).

Bottino, L. Z. M. F., et al. (2021). "Extracellular vesicles derived from head and neck squamous cells carcinoma inhibit NLRP3 inflammasomes." Current Research in Immunology **2**: 175-183.

Bozyk, N., et al. (2023). "Salivary exosomes as biomarkers for early diagnosis of oral squamous cell carcinoma." Oral Oncology Reports **6**.

Bugajova, M., et al. (2024). "Glutamine and serum starvation alters the ATP production, oxidative stress, and abundance of mitochondrial RNAs in extracellular vesicles produced by cancer cells." Scientific Reports **14**(1).

Busso-Lopes, A. F., et al. (2018). "Multi-omics data indicate that primary and lymph node oral cancer cells-derived extracellular vesicles carry cargo molecules with a specific aggressive pattern." CANCER RESEARCH **78**(13).

Cai, J., et al. (2019). "Oral squamous cell carcinoma-derived exosomes promote M2 subtype macrophage polarization mediated by exosome-enclosed miR-29a-3p." American Journal of Physiology - Cell Physiology **316**(5): C731-C740.

Cela, I., et al. (2024). "LGALS3BP is a potential target of antibody-drug conjugates in oral squamous cell carcinoma." Oral Dis.

Cema, I., et al. (2021). "Correlation of Soluble CD44 Expression in Saliva and CD44 Protein in Oral Leukoplakia Tissues." CANCERS **13**(22).

Chen, G., et al. (2023). "Fusobacterium nucleatum outer membrane vesicles activate autophagy to promote oral cancer metastasis." Journal of Advanced Research.

Chen, L. C., et al. (2018). "Exosomes derived from HIV-1-infected cells promote growth and progression of cancer via HIV TAR RNA." NATURE COMMUNICATIONS **9**.

Chen, X., et al. (2025). "Spermine accumulation via spermine synthase promotes tumor cell proliferation in head and neck squamous cell carcinoma." BMC Cancer **25**(1).

Chen, Y. Y., et al. (2022). "CircRNA has_circ_0069313 induced OSCC immunity escape by miR-325-3p-Foxp3 axes in both OSCC cells and Treg cells." AGING-US **14**(10): 4376-4389.

Cheng, Y., et al. (2024). "Selective detection of exosomes by elemental labeling ICP-MS based on cholesterol-EpCAM aptamer proximity ligation mediated MNAzyme assembly." Sensors and Actuators B: Chemical **416**: 136013.

Cohen, O., et al. (2021). "'Golden' exosomes as delivery vehicles to target tumors and overcome intratumoral barriers:: In vivo tracking in a model for head and neck cancer." Biomaterials Science **9**(6): 2103-2114.

de Andrade, A., et al. (2018). "Extracellular vesicles from oral squamous carcinoma cells display pro- and anti-angiogenic properties." Oral Dis **24**(5): 725-731.

Deng, W., et al. (2022). "<i>In vitro</i> experimental study on the formation of microRNA-34a loaded exosomes and their inhibitory effect in oral squamous cell carcinoma." Cell Cycle **21**(16): 1775-1783.

Ding, B., et al. (2024). "In Vitro and In Vivo Analyses Reveal Tumor-Derived Exosome miR-558 Promotes Angiogenesis in Tongue Squamous Cell Carcinoma by Targeting Heparinase." Technology in Cancer Research & Treatment **23**.

Dong, Y., et al. (2023). "Regulation of Exosomes-Mediated circNR4A1 on Chemoresistance and Biological Effects of Oral Squamous Cell Carcinoma Cells." Letters in Drug Design and Discovery **20**(7): 921-929.

Dourado, M. R., et al. (2019). "Extracellular vesicles derived from cancer-associated fibroblasts induce the migration and invasion of oral squamous cell carcinoma." Journal of Extracellular Vesicles **8**(1).

Duan, Y., et al. (2024). "Hypoxia-induced miR-5100 promotes exosome-mediated activation of cancer-associated fibroblasts and metastasis of head and neck squamous cell carcinoma." Cell Death & Disease **15**(3).

El‐Labban, N. G. and E. Osorio‐Herrera (1986). "Apoptotic bodies and abnormally dividing epithelial cells in squamous cell carcinoma." Histopathology **10**(9): 921-931.

Faur, C. I., et al. (2023). "A New Detection Method of Oral and Oropharyngeal Squamous Cell Carcinoma Based on Multivariate Analysis of Surface Enhanced Raman Spectra of Salivary Exosomes." Journal of Personalized Medicine **13**(5).

Feng, J., et al. (2023). "Simultaneous Detection of Two Extracellular Vesicle Subpopulations in Saliva Assisting Tumor T Staging of Oral Squamous Cell Carcinoma." ANALYTICAL CHEMISTRY **95**(19): 7753-7760.

Flemming, P. J., et al. (2020). "miRNA‐ and cytokine‐associated extracellular vesicles mediate squamous cell carcinomas." Journal of Extracellular Vesicles **9**(1): 1790159.

Fu, Q.-Y., et al. (2024). "Spatiotemporal characteristics of tissue derived small extracellular vesicles is associated with tumor relapse and anti-PD-1 response." Cancer Letters **591**: 216897.

Fujiwara, T., et al. (2018). "Carcinogenic epithelial-mesenchymal transition initiated by oral cancer exosomes is inhibited by anti-EGFR antibody cetuximab." ORAL ONCOLOGY **86**: 251-257.

Galvão, F., et al. (2023). "MiRNAs that target amyloid precursor protein processing machinery in extracellular vesicles and particles derived from oral squamous cells carcinoma." Journal of Oral Pathology and Medicine **52**(9): 877-884.

Gluszko, A., et al. (2021). "Small Extracellular Vesicles from Head and Neck Squamous Cell Carcinoma Cells Carry a Proteomic Signature for Tumor Hypoxia." CANCERS **13**(16).

Gong, W., et al. (2021). "Cancer-specific type-I interferon receptor signaling promotes cancer stemness and effector CD8+T-cell exhaustion." ONCOIMMUNOLOGY **10**(1).

Gonzalez-Callejo, P., et al. (2023). "Cancer stem cell-derived extracellular vesicles preferentially target MHC-II-macrophages and PD1+T cells in the tumor microenvironment." PLOS ONE **18**(2).

Goudsmit, C., et al. (2021). "Differences in Extracellular Vesicle Protein Cargo Are Dependent on Head and Neck Squamous Cell Carcinoma Cell of Origin and Human Papillomavirus Status." CANCERS **13**(15).

Guerreiro, E. M., et al. (2020). "Cancer cell line-specific protein profiles in extracellular vesicles identified by proteomics." PLOS ONE **15**(9).

Guerreiro, E. M., et al. (2018). "Efficient extracellular vesicle isolation by combining cell media modifications, ultrafiltration, and size-exclusion chromatography." PLOS ONE **13**(9).

Guo, E., et al. (2021). "Alternatively spliced ANLN isoforms synergistically contribute to the progression of head and neck squamous cell carcinoma." Cell Death and Disease **12**(8).

Guo, H., et al. (2021). "Serum exosome-derived biomarkers for the early detection of oral squamous cell carcinoma." Molecular and Cellular Biochemistry **476**(12): 4435-4447.

Ha, Y. J., et al. (2025). "Transcriptome Analysis of <scp>HNSCC</scp> by <i>Aggregatibacter actinomycetemcomitans</i> Extracellular Vesicles." Oral Diseases **31**(4): 1165-1177.

Han, B., et al. (2021). "Adipose-Derived Stem Cell-Derived Extracellular Vesicles Inhibit the Fibrosis of Fibrotic Buccal Mucosal Fibroblasts via the MicroRNA-375/FOXF1 Axis." STEM CELLS INTERNATIONAL **2021**.

Han, N., et al. (2023). "Cancer cell-derived extracellular vesicles drive pre-metastatic niche formation of lymph node via IFNGR1/JAK1/STAT1-activated-PD-L1 expression on FRCs in head and neck cancer." Oral Oncology **145**.

He, L., et al. (2020). "Salivary exosomal miR-24-3p serves as a potential detective biomarker for oral squamous cell carcinoma screening." Biomedicine and Pharmacotherapy **121**.

He, L., et al. (2021). "Engineered red blood cell membrane for sensitive and precise electrochemical detection of salivary exosomes." Analytical Methods **13**(48): 5859-5865.

He, L. H., et al. (2023). "Exosomal miR-146b-5p derived from cancer-associated fibroblasts promotes progression of oral squamous cell carcinoma by downregulating HIPK3." CELLULAR SIGNALLING **106**.

He, S., et al. (2021). "Oral squamous cell carcinoma (OSCC)-derived exosomal MiR-221 targets and regulates phosphoinositide-3-kinase regulatory subunit 1 (PIK3R1) to promote human umbilical vein endothelial cells migration and tube formation." Bioengineered **12**(1): 2164-2174.

He, T., et al. (2021). "Plasma-Derived Exosomal microRNA-130a Serves as a Noninvasive Biomarker for Diagnosis and Prognosis of Oral Squamous Cell Carcinoma." Journal of Oncology **2021**.

Higaki, M., et al. (2020). "Eldecalcitol (ED-71)-induced exosomal miR-6887-5p suppresses squamous cell carcinoma cell growth by targeting heparin-binding protein 17/fibroblast growth factor-binding protein-1 (HBp17/FGFBP-1)." IN VITRO CELLULAR & DEVELOPMENTAL BIOLOGY-ANIMAL **56**(3): 222-233.

Hill, B. L., et al. (2023). "IL-8 correlates with nonresponse to neoadjuvant nivolumab in HPV positive HNSCC via a potential extracellular vesicle miR-146a mediated mechanism." MOLECULAR CARCINOGENESIS.

Hofmann, L., et al. (2022). "Comparison of plasma- and saliva-derived exosomal miRNA profiles reveals diagnostic potential in head and neck cancer." FRONTIERS IN CELL AND DEVELOPMENTAL BIOLOGY **10**.

Hofmann, L., et al. (2023). "Arginase-1 in Plasma-Derived Exosomes as Marker of Metastasis in Patients with Head and Neck Squamous Cell Carcinoma." CANCERS **15**(22).

Hong, C. S., et al. (2016). "Isolation of biologically active and morphologically intact exosomes from plasma of patients with cancer." Journal of Extracellular Vesicles **5**(1): 29289.

Hong, S. L., et al. (2022). "One-step detection of oral ulcers and oral cancer derived exosomes on wedge-shaped and high magnetic field gradient mediated chip." Sensors and Actuators B: Chemical **357**.

Hu, K. F., et al. (2025). "Regulation of Exosomal <scp>miR</scp>‐320d/<scp>FAM49B</scp> Axis by Guanylate Binding Protein 5 Promotes Cell Growth and Tumor Progression in Oral Squamous Cell Carcinoma." Journal of Oral Pathology & Medicine **54**(5): 298-311.

Huang, C.-Y., et al. (2024). "MEG3-Mediated Oral Squamous-Cell-Carcinoma-Derived Exosomal miR-421 Activates Angiogenesis by Targeting HS2ST1 in Vascular Endothelial Cells." International Journal of Molecular Sciences **25**(14): 7576.

Huang, Q., et al. (2021). "Small extracellular vesicle-packaged TGFS1 promotes the reprogramming of normal fibroblasts into cancer-associated fibroblasts by regulating fibronectin in head and neck squamous cell carcinoma." CANCER LETTERS **517**: 1-13.

Huang, W. X., et al. (2023). "Investigating whether exosomal miR-205-5p derived from tongue squamous cell carcinoma cells stimulates the angiogenic activity of HUVECs by targeting AMOT." CANCER BIOMARKERS **38**(2): 215-224.

Inyang, E. K., et al. (2022). Head and Neck Cancer-derived small extracellular vesicles sensitize TRPV1+ neurons to mediate cancer pain.

Inyang, E. K., et al. (2024). "HPV+ head and neck cancer–derived small extracellular vesicles communicate with TRPV1+ neurons to mediate cancer pain." Pain **165**(3): 608-620.

Jelonek, K., et al. (2015). "Ionizing radiation affects protein composition of exosomes secreted in vitro from head and neck squamous cell carcinoma." Acta Biochimica Polonica **62**(2): 265-272.

Jha, A., et al. (2023). "Crosstalk between PD-L1 and Jak2-Stat3/ MAPK-AP1 signaling promotes oral cancer progression, invasion and therapy resistance." Int Immunopharmacol **124**: 110894.

Ji, Y., et al. (2019). "Multiplexed profiling of single-cell extracellular vesicles secretion." Proceedings of the National Academy of Sciences of the United States of America **116**(13): 5979-5984.

Jiang, E., et al. (2019). "Tumoral microvesicle–activated glycometabolic reprogramming in fibroblasts promotes the progression of oral squamous cell carcinoma." FASEB Journal **33**(4): 5690-5703.

Jiang, H., et al. (2022). "M1 macrophage-derived exosomes and their key molecule lncRNA HOTTIP suppress head and neck squamous cell carcinoma progression by upregulating the TLR5/NF-κB pathway." Cell Death and Disease **13**(2).

Jin, J., et al. (2020). "Bioinformatics analysis of aberrantly expressed exosomal lncRNAs in oral squamous cell carcinoma (CAL.27 vs. oral epithelial) cells." Oncology Letters **20**(3): 2378-2386.

Jin, N., et al. (2020). "Long non-coding RNA TIRY promotes tumor metastasis by enhancing epithelial-to-mesenchymal transition in oral cancer." EXPERIMENTAL BIOLOGY AND MEDICINE **245**(7): 585-596.

Juvkam, I. S., et al. (2023). "Proton Compared to X-Irradiation Induces Different Protein Profiles in Oral Cancer Cells and Their Derived Extracellular Vesicles." International Journal of Molecular Sciences **24**(23).

Kang, S. H., et al. (2024). "Differential effect of cancer-associated fibroblast-derived extracellular vesicles on cisplatin resistance in oral squamous cell carcinoma via miR-876-3p." Theranostics **14**(2): 460-479.

Kim, J. W., et al. (2005). "Fas ligand-positive membranous vesicles isolated from sera of patients with oral cancer induce apoptosis of activated T lymphocytes." CLINICAL CANCER RESEARCH **11**(3): 1010-1020.

Ko, H. H., et al. (2025). "Downregulation of <scp>PAX1</scp> in <scp>OSCC</scp> Enhances Stemness and Immunosuppression via <scp>IFIT1</scp> and <scp>PD</scp>‐<scp>L1</scp> Pathways." Oral Diseases **31**(7): 2071-2083.

Kulkarni, B., et al. (2020). "Exosome-mediated delivery of miR-30a sensitize cisplatin-resistant variant of oral squamous carcinoma cells via modulating Beclin1 and Bcl2." Oncotarget **11**(20): 1832-1845.

Langevin, S., et al. (2017). "Comprehensive microRNA-sequencing of exosomes derived from head and neck carcinoma cells in vitro reveals common secretion profiles and potential utility as salivary biomarkers." ONCOTARGET **8**(47): 82459-82474.

Languino, L. R., et al. (2016). "Exosome-mediated transfer from the tumor microenvironment increases TGFβ signaling in squamous cell carcinoma." AMERICAN JOURNAL OF TRANSLATIONAL RESEARCH **8**(5): 2432-2437.

Lee, J. C., et al. (2020). "HNC0014, a Multi-Targeted Small-Molecule, Inhibits Head and Neck Squamous Cell Carcinoma by Suppressing c-Met/STAT3/CD44/PD-L1 Oncoimmune Signature and Eliciting Antitumor Immune Responses." CANCERS **12**(12).

Li, C., et al. (2025). "Lymphocytes‐Associated Extracellular Vesicles Activate Natural Killer Cells in HNSCC." Cancer Science **116**(3): 633-642.

Li, C. P., et al. (2022). "Exosomal long noncoding RNAs MAGI2-AS3 and CCDC144NL-AS1 in oral squamous cell carcinoma development via the PI3K-AKT-mTOR signaling pathway." PATHOLOGY RESEARCH AND PRACTICE **240**.

Li, C. P., et al. (2019). "Potential Markers from Serum-Purified Exosomes for Detecting Oral Squamous Cell Carcinoma Metastasis." CANCER EPIDEMIOLOGY BIOMARKERS & PREVENTION **28**(10): 1668-1681.

Li, J., et al. (2023). "M2 Macrophages-Derived Exosomal miRNA-23a-3p Promotes the Progression of Oral Squamous Cell Carcinoma by Targeting PTEN." Current Issues in Molecular Biology **45**(6): 4936-4947.

Li, J., et al. (2020). "Small extracellular vesicle-bound vascular endothelial growth factor secreted by carcinoma-associated fibroblasts promotes angiogenesis in a bevacizumab-resistant manner." Cancer Letters **492**: 71-83.

Li, K., et al. (2022). "Biomimetic Nanosystems for the Synergistic Delivery of miR-144/451a for Oral Squamous Cell Carcinoma." Balkan Medical Journal **39**(3): 178-186.

Li, L.-S., et al. (2025). "A label-free fluorescence aptasensor for salivary exosomes based on a nano-micro dual-scale signal amplification strategy." Talanta **287**: 127638.

Li, L., et al. (2019). "Microenvironmental oxygen pressure orchestrates an anti- and pro-tumoral γδ T cell equilibrium via tumor-derived exosomes." Oncogene **38**(15): 2830-2843.

Li, L., et al. (2016). "Exosomes Derived from Hypoxic Oral Squamous Cell Carcinoma Cells Deliver miR-21 to Normoxic Cells to Elicit a Prometastatic Phenotype." CANCER RESEARCH **76**(7): 1770-1780.

Li, L., et al. (2019). "γδTDEs: An Efficient Delivery System for miR-138 with Anti-tumoral and Immunostimulatory Roles on Oral Squamous Cell Carcinoma." Molecular Therapy Nucleic Acids **14**: 101-113.

Li, M., et al. (2023). "Synergistic Phototherapy-Molecular Targeted Therapy Combined with Tumor Exosome Nanoparticles for Oral Squamous Cell Carcinoma Treatment." Pharmaceutics **16**(1): 33.

Li, R. W., et al. (2023). "Oral squamous cell carcinoma-derived EVs promote tumor progression by regulating inflammatory cytokines and the IL-17A-induced signaling pathway." INTERNATIONAL IMMUNOPHARMACOLOGY **118**.

Li, S., et al. (2021). "Exosomal-mediated transfer of APCDD1L-AS1 induces 5-fluorouracil resistance in oral squamous cell carcinoma via miR-1224-5p/nuclear receptor binding SET domain protein 2 (NSD2) axis." Bioengineered **12**(1): 7188-7204.

Li, S. F., et al. (2022). "Mesenchymal stem cell-exosome-mediated matrix metalloproteinase 1 participates in oral leukoplakia and carcinogenesis by inducing angiogenesis." JOURNAL OF ORAL PATHOLOGY & MEDICINE **51**(7): 638-648.

Li, W. W., et al. (2019). "Oral mucosal mesenchymal stem cell-derived exosomes: A potential therapeutic target in oral premalignant lesions." INTERNATIONAL JOURNAL OF ONCOLOGY **54**(5): 1567-1578.

Li, Y., et al. (2024). "A Group of Highly Secretory miRNAs Correlates with Lymph Node Metastasis and Poor Prognosis in Oral Squamous Cell Carcinoma." Biomolecules **14**(2): 224.

Li, Y., et al. (2024). "Exosomal microRNA let-7c-5p enhances cell malignant characteristics by inhibiting TAGLN in oral cancer." Oncology Research **32**(10): 1623-1635.

Li, Y. Y., et al. (2018). "Cancer-associated fibroblasts contribute to oral cancer cells proliferation and metastasis via exosome-mediated paracrine miR-34a-5p." EBioMedicine **36**: 209-220.

Li, Z.-Z., et al. (2024). "Lymph node metastasis diagnosis of postoperative OSCC patients by analyzing extracellular vesicles in drainage fluid based on microfluidic isolation." Journal of Nanobiotechnology **22**(1).

Lipka, A., et al. (2025). "The effect of extracellular vesicles derived from oral squamous cell carcinoma on the metabolic profile of oral fibroblasts." Frontiers in Molecular Biosciences **12**.

Liu, J., et al. (2025). "<scp>TAM</scp>‐Derived Exosomes Promote <scp>EMT</scp> by Upregulating <scp>lncRNA MIR4435</scp>‐<scp>2HG</scp> in Head and Neck Cancer." Oral Diseases **31**(4): 1154-1164.

Liu, J. J., et al. (2021). "Adipose-derived mesenchymal stem cell exosomes inhibit transforming growth factor-β1-induced collagen synthesis in oral mucosal fibroblasts." EXPERIMENTAL AND THERAPEUTIC MEDICINE **22**(6).

Liu, P., et al. (2022). "Exosomes derived from stem cells of human deciduous exfoliated teeth inhibit angiogenesis in vivo and in vitro via the transfer of miR-100-5p and miR-1246." Stem Cell Research and Therapy **13**(1).

Liu, Q., et al. (2025). "Light-activated photosensitizer/quercetin co-loaded extracellular vesicles for precise oral squamous cell carcinoma therapy." International Journal of Pharmaceutics **671**: 125224.

Liu, T., et al. (2017). "Exosomes containing miR-21 transfer the characteristic of cisplatin resistance by targeting PTEN and PDCD4 in oral squamous cell carcinoma." ACTA BIOCHIMICA ET BIOPHYSICA SINICA **49**(9): 808-816.

Liu, T., et al. (2024). "A Comprehensive Analysis of Exosome‐Related Long Noncoding RNAs as Prognostic Biomarkers and Therapeutic Targets in Head and Neck Squamous Cell Carcinoma." International Journal of Clinical Practice **2024**(1).

Liu, X., et al. (2023). "Carcinoma-associated fibroblast-derived lysyl oxidase-rich extracellular vesicles mediate collagen crosslinking and promote epithelial-mesenchymal transition via p-FAK/p-paxillin/YAP signaling." INTERNATIONAL JOURNAL OF ORAL SCIENCE **15**(1).

Liu, Y. H., et al. (2021). "Nanoscale biomimetic nano system for the co-delivery of SNS032 and tumor necrosis factor related apoptosis inducing ligand to enhance therapeutic efficacy in oral squamous cell carcinoma cell line SCC25." MATERIALS EXPRESS **11**(8): 1321-1330.

Lou, C., et al. (2022). "Exosomal miR-626 promotes the malignant behavior of oral cancer cells by targeting NFIB." Molecular Biology Reports **49**(6): 4829-4840.

Lu, Y. Y., et al. (2021). "Exosome-Based Molecular Transfer Activity of Macrophage-Like Cells Involves Viability of Oral Carcinoma Cells: Size Exclusion Chromatography and Concentration Filter Method." CELLS **10**(6).

Ludwig, N., et al. (2020). "Tumor-derived exosomes promote angiogenesis via adenosine A2B receptor signaling." Angiogenesis **23**(4): 599-610.

Ludwig, N., et al. (2022). "TGFβ<SUP>+</SUP> small extracellular vesicles from head and neck squamous cell carcinoma cells reprogram macrophages towards a pro-angiogenic phenotype." JOURNAL OF EXTRACELLULAR VESICLES **11**(12).

Ludwig, N., et al. (2023). "TGFβ carrying exosomes in plasma: potential biomarkers of cancer progression in patients with head and neck squamous cell carcinoma." British Journal of Cancer **128**(9): 1733-1741.

Ludwig, N., et al. (2023). "TGF-β carrying exosomes in plasma of HNSCC as potential biomarkers of disease progression in patients with HNSCC." CLINICAL CANCER RESEARCH **29**(18).

Ludwig, S., et al. (2020). "Mrna and mirna profiles of exosomes from cultured tumor cells reveal biomarkers specific for hpv16-positive and hpv16-negative head and neck cancer." International Journal of Molecular Sciences **21**(22): 1-15.

Luo, Y. W., et al. (2020). "Upregulation of circ_0000199 in circulating exosomes is associated with survival outcome in OSCC." SCIENTIFIC REPORTS **10**(1).

Lv, T., et al. (2024). "Cancer-associated fibroblast-derived extracellular vesicles promote lymph node metastases in oral cavity squamous cell carcinoma by encapsulating ITGB1 and BMI1." BMC Cancer **24**(1).

Madhu, I. and A. Kannan (2025). "Exosomal 4EBP1 promotes head and neck cancer progression via regulating mitochondrial fission." Biochemical and Biophysical Research Communications **761**: 151735.

Man, Q.-W., et al. (2023). "Prognostic value of CD8-to-EGFR ratio in salivary microvesicles of patients with oral squamous cell carcinoma." Oral Dis **29**(4): 1480-1486.

Maybruck, B. T., et al. (2017). "Tumor-derived exosomes induce CD8+ T cell suppressors." Journal for ImmunoTherapy of Cancer **5**(1).

Mayne, G. C., et al. (2022). "Cross validated serum small extracellular vesicle microRNAs for the detection of oropharyngeal squamous cell carcinoma (vol 18, 280, 2020)." JOURNAL OF TRANSLATIONAL MEDICINE **20**(1).

Menck, K., et al. (2017). "Characterisation of tumour-derived microvesicles in cancer patients' blood and correlation with clinical outcome." JOURNAL OF EXTRACELLULAR VESICLES **6**(1): 1-16.

Metsäniitty, M., et al. (2024). "Extracellular vesicles from Aggregatibacter actinomycetemcomitans exhibit potential antitumorigenic effects in oral cancer: a comparative in vitro study." Archives of Microbiology **206**(6).

Metsäniitty, M., et al. (2024). "Zebrafish larvae as a model for studying the impact of oral bacterial vesicles on tumor cell growth and metastasis." Human Cell **37**(6): 1696-1705.

Miao, C., et al. (2024). "OSCC-derived EVs educate fibroblasts and remodel collagen landscape." Matrix Biology **134**: 132-143.

Momen-Heravi, F. and S. Bala (2018). "Extracellular vesicles in oral squamous carcinoma carry oncogenic miRNA profile and reprogramme monocytes via NF-κB pathway." Oncotarget **9**(78): 34838-34854.

Mutschelknaus, L., et al. (2017). "Radiation alters the cargo of exosomes released from squamous head and neck cancer cells to promote migration of recipient cells." Scientific Reports **7**(1).

Nakamichi, E., et al. (2021). "Detection of serum/salivary exosomal Alix in patients with oral squamous cell carcinoma." Oral Dis **27**(3): 439-447.

Ogawa, T., et al. (2024). "Novel mechanism of cisplatin resistance in head and neck squamous cell carcinoma involving extracellular vesicles and a copper transporter system." HEAD AND NECK-JOURNAL FOR THE SCIENCES AND SPECIALTIES OF THE HEAD AND NECK.

Omori, Y., et al. (2024). "Bacterial Lipopolysaccharide Induces PD-L1 Expression and an Invasive Phenotype of Oral Squamous Cell Carcinoma Cells." Cancers **16**(2): 343.

Ono, K., et al. (2018). "HSP-enriched properties of extracellular vesicles involve survival of metastatic oral cancer cells." CANCER SCIENCE **109**: 621-621.

Ono, K., et al. (2020). "Triple knockdown of CDC37, HSP90-alpha and HSP90-beta diminishes extracellular vesicles-driven malignancy events and macrophage M2 polarization in oral cancer." JOURNAL OF EXTRACELLULAR VESICLES **9**(1).

Overmiller, A. M., et al. (2017). "Desmoglein 2 modulates extracellular vesicle release from squamous cell carcinoma keratinocytes." FASEB JOURNAL **31**(8): 3412-3424.

Pande, K., et al. (2024). "A biocompatible nanoformulation of curcumin analogue and curd exosomes targeting EphA2 signalling cascade in head and neck cancer." Cancer Nanotechnology **15**(1).

Pang, X., et al. (2021). "OSCC cell-secreted exosomal CMTM6 induced M2-like macrophages polarization via ERK1/2 signaling pathway." Cancer Immunology, Immunotherapy **70**(4): 1015-1029.

Pelaez-Prestel, F. H., et al. (2024). "Oral squamous cell carcinomas drive monocytes into immunosuppressive CD25+CD163+CD206+ macrophages." Oral Oncology **159**: 107078.

Principe, S., et al. (2018). "Proteomic Analysis of Cancer-Associated Fibroblasts Reveals a Paracrine Role for MFAP5 in Human Oral Tongue Squamous Cell Carcinoma." Journal of Proteome Research **17**(6): 2045-2059.

Qadir, F., et al. (2018). "Transcriptome reprogramming by cancer exosomes: Identification of novel molecular targets in matrix and immune modulation." Molecular Cancer **17**(1).

Qin, X., et al. (2019). "Exosomal miR-196a derived from cancer-associated fibroblasts confers cisplatin resistance in head and neck cancer through targeting CDKN1B and ING5." Genome Biology **20**(1).

Qiu, L., et al. (2020). "Exosomes of oral squamous cell carcinoma cells containing miR-181a-3p induce muscle cell atrophy and apoptosis by transmissible endoplasmic reticulum stress signaling." Biochemical and Biophysical Research Communications **533**(4): 831-837.

Qiu, Y., et al. (2020). "Antitumor activity of cabazitaxel and msc-trail derived extracellular vesicles in drug-resistant oral squamous cell carcinoma." Cancer Management and Research **12**: 10809-10820.

Qu, X., et al. (2021). "Proteomic analysis of circulating extracellular vesicles identifies potential biomarkers for lymph node metastasis in oral tongue squamous cell carcinoma." Cells **10**(9).

Rabinowits, G., et al. (2017). "Comparative analysis of microrna expression among benign and malignant tongue tissue and plasma of patients with tongue cancer." Frontiers in Oncology **7**.

Ramayanti, O., et al. (2019). "Vesicle-bound EBV-BART13-3p miRNA in circulation distinguishes nasopharyngeal from other head and neck cancer and asymptomatic EBV-infections." International Journal of Cancer **144**(10): 2555-2566.

Razzo, B., et al. (2020). "Tumor-derived exosomes promote carcinogenesis of murine oral squamous cell carcinoma." Carcinogenesis **41**(5): 625-633.

Rizwan, M., et al. (2025). "Deregulation of Exosomal miR-17, miR-20a and TGFBR2 in Head and Neck Cancer Patients." Technology in Cancer Research & Treatment **24**.

Rodrigues-Junior, D. M., et al. (2019). "A preliminary investigation of circulating extracellular vesicles and biomarker discovery associated with treatment response in head and neck squamous cell carcinoma." BMC Cancer **19**(1).

Rodrigues-Junior, D. M., et al. (2019). "Circulating extracellular vesicle-associated TGFβ3 modulates response to cytotoxic therapy in head and neck squamous cell carcinoma." Carcinogenesis **40**(12): 1452-1461.

Rubenich, S. D., et al. (2024). "The immunomodulatory ballet of tumour‐derived extracellular vesicles and neutrophils orchestrating the dynamic CD73/PD‐L1 pathway in cancer." Journal of Extracellular Vesicles **13**(7).

Sakha, S., et al. (2016). "Exosomal microRNA miR-1246 induces cell motility and invasion through the regulation of DENND2D in oral squamous cell carcinoma." Scientific Reports **6**.

Sanada, T., et al. (2020). "Elevated exosomal lysyl oxidase like 2 is a potential biomarker for head and neck squamous cell carcinoma." Laryngoscope **130**(5): E327-E334.

Sato, K., et al. (2025). "Nationwide multi-centric prospective study for the identification of biomarkers to predict the treatment responses of nivolumab through comprehensive analyses of pretreatment plasma exosome mRNAs from head and neck cancer patients (BIONEXT study)." Frontiers in Immunology **15**.

Sato, S., et al. (2019). "EPHB2 carried on small extracellular vesicles induces tumor angiogenesis via activation of ephrin reverse signaling." JCI INSIGHT **4**(23).

Seki, Y., et al. (2024). "Programmed death-ligand 1-expressing extracellular vesicles are a prognostic factor in patients with oral squamous cell carcinoma treated with immune checkpoint inhibitors." Journal of Oral and Maxillofacial Surgery, Medicine, and Pathology.

Shafiaa, A. S. E., et al. (2022). "Effect of Mesenchymal Stem Cell-Derived Exosomes on Head & Neck Squamous Cell Carcinoma Cell Line in-Vitro Study." Ain Shams Dental Journal (Egypt) **25**(1): 74-83.

Shoff, M., et al. (2020). "Differential exosome mirna expression in oral cancer stem cells." ExRNA **2**.

Silva, E. D., et al. (2021). "Extracellular vesicles cargo from head and neck cancer cell lines disrupt dendritic cells function and match plasma microRNAs." SCIENTIFIC REPORTS **11**(1).

Smolarz, M., et al. (2022). "Radiation-Induced Bystander Effect Mediated by Exosomes Involves the Replication Stress in Recipient Cells." INTERNATIONAL JOURNAL OF MOLECULAR SCIENCES **23**(8).

Søland, M. T., et al. (2024). "Extracellular vesicles from cancer cell lines of different origins drive the phenotype of normal oral fibroblasts in a CAF-like direction." Frontiers in Oncology **14**.

Sun, J., et al. (2023). "Proteomic and phosphoproteomic landscape of salivary extracellular vesicles to assess OSCC therapeutical outcomes." Proteomics **23**(5).

Sun, L.-P., et al. (2019). "Cancer-associated fibroblast-derived exosomal miR-382-5p promotes the migration and invasion of oral squamous cell carcinoma." Oncol Rep **42**(4): 1319-1328.

Sun, Y. Q., et al. (2024). "Oral cancer cell to endothelial cell communication via exosomal miR-21/RMND5A pathway." BMC Oral Health **24**(1).

Tamkovich, S. N., et al. (2018). "Comparative Subpopulation Analysis of Plasma Exosomes from Cancer Patients." BIOCHEMISTRY MOSCOW-SUPPLEMENT SERIES B-BIOMEDICAL CHEMISTRY **12**(2): 151-155.

Tang, K. D., et al. (2021). "Proteomic Alterations in Salivary Exosomes Derived from Human Papillomavirus-Driven Oropharyngeal Cancer." MOLECULAR DIAGNOSIS & THERAPY **25**(4): 505-515.

Tengler, L., et al. (2023). "Plasma-derived small extracellular vesicles unleash the angiogenic potential in head and neck cancer patients." MOLECULAR MEDICINE **29**(1).

Tengler, L., et al. (2024). "Optimization of extracellular vesicles preparation from saliva of head and neck cancer patients." Scientific Reports **14**(1).

Theodoraki, M.-N., et al. (2023). "Plasma-derived CD16 exosomes and peripheral blood monocytes as correlating biomarkers in head and neck cancer." Oncol. Lett. **25**(5): 200.

Theodoraki, M.-N., et al. (2024). "Type 2-like polarization and elevated CXCL4 secretion of monocyte derived macrophages upon internalization of plasma-derived exosomes from head and neck cancer patients." BMC Cancer **24**(1).

Theodoraki, M. N., et al. (2018). "Separation of plasma-derived exosomes into CD3<SUP>(+)</SUP> and CD3<SUP>(-)</SUP> fractions allows for association of immune cell and tumour cell markers with disease activity in HNSCC patients." CLINICAL AND EXPERIMENTAL IMMUNOLOGY **192**(3): 271-283.

Theodoraki, M. N., et al. (2018). "Clinical significance of PD-L1 levels in plasma-derived exosomes in head and neck cancer." JOURNAL OF CLINICAL ONCOLOGY **36**(5).

Toh, Y. S., et al. (2024). "Therapeutic application of extracellular vesicular EGFR isoform D as a co-drug to target squamous cell cancers with tyrosine kinase inhibitors." Developmental Cell **59**(16): 2189-2202.e2188.

Tomita, R., et al. (2020). "Macrophage-derived exosomes attenuate the susceptibility of oral squamous cell carcinoma cells to chemotherapeutic drugs through the AKT/GSK-3[beta] pathway." Oncol Rep **44**(5): 1905-1916.

Tong, F., et al. (2020). "HPV + HNSCC-derived exosomal miR-9 induces macrophage M1 polarization and increases tumor radiosensitivity." Cancer Letters **478**: 34-44.

Vasiljevic, T., et al. (2023). "Necrotic Cells from Head and Neck Carcinomas Release Biomolecules That Are Activating Toll-like Receptor 3." International Journal of Molecular Sciences **24**(20).

Vered, M., et al. (2015). "Caveolin-1 accumulation in the tongue cancer tumor microenvironment is significantly associated with poor prognosis: an <i>in-vivo</i> and <i>in-vitro</i> study." BMC CANCER **15**.

Wang, B., et al. (2022). "HPV<sup>+</sup> HNSCC‐derived exosomal miR‐9‐5p inhibits TGF‐β signaling‐mediated fibroblast phenotypic transformation through NOX4." Cancer Science **113**(4): 1475-1487.

Wang, C., et al. (2019). "Exosome-delivered TRPP2 siRNA inhibits the epithelial-mesenchymal transition of FaDu cells." Oncology Letters **17**(2): 1953-1961.

Wang, D., et al. (2025). "Tetrahedral-DNA-Nanostructure-Modified Engineered Extracellular Vesicles Enhance Oral Squamous Cell Carcinomas Therapy by Targeting GPX4." ACS Nano **19**(9): 9351-9366.

Wang, H., et al. (2020). "OSCC Exosomes Regulate miR-210-3p Targeting EFNA3 to Promote Oral Cancer Angiogenesis through the PI3K/AKT Pathway." BIOMED RESEARCH INTERNATIONAL **2020**.

Wang, H., et al. (2023). "MicroRNA-21 promotes head and neck squamous cell carcinoma (HNSCC) induced transition of bone marrow mesenchymal stem cells to cancer-associated fibroblasts." BMC CANCER **23**(1).

Wang, L., et al. (2019). "Delivery of mesenchymal stem cells-derived extracellular vesicles with enriched miR-185 inhibits progression of OPMD." Artificial Cells, Nanomedicine, and Biotechnology **47**(1): 2481-2491.

Wang, S.-H., et al. (2019). "Laminin [gamma]2-enriched extracellular vesicles of oral squamous cell carcinoma cells enhance in vitro lymphangiogenesis via integrin [alpha]3-dependent uptake by lymphatic endothelial cells." Int. j. cancer **144**(11): 2795-2810.

Wang, W. C., et al. (2022). "Salivary Exosome Proteomics and Bioinformatics Analysis in 7,12-Dimethylbenz[a]anthracene-Induced Oral Cancer with Radiation Therapy-A Syrian Golden Hamster Model." DIAGNOSTICS **12**(1).

Wang, X. N., et al. (2019). "Loss of exosomal miR-3188 in cancer-associated fibroblasts contributes to HNC progression." JOURNAL OF EXPERIMENTAL & CLINICAL CANCER RESEARCH **38**.

Wang, X. P., et al. (2021). "Targeting miR-185-3p Inhibits Head and Neck Squamous Cell Carcinoma by Modulating RAB25." FRONTIERS IN ONCOLOGY **11**.

Wang, Y., et al. (2025). "Hypoxia regulates small extracellular vesicle biogenesis and cargo sorting through HIF-1α/HRS signaling pathway in head and neck squamous cell carcinoma." Cellular Signalling **127**: 111546.

Wang, Y. N., et al. (2018). "Oral cancer-derived exosomal NAP1 enhances cytotoxicity of natural killer cells via the IRF-3 pathway." ORAL ONCOLOGY **76**: 34-41.

Wang, Z. Y., et al. (2022). "The Endoplasmic Reticulum-Stressed Head and Neck Squamous Cell Carcinoma Cells Induced Exosomal miR-424-5p Inhibits Angiogenesis and Migration of Humanumbilical Vein Endothelial Cells Through LAMC1-Mediated Wnt/β-Catenin Signaling Pathway." CELL TRANSPLANTATION **31**.

Wang, Z. Y., et al. (2020). "Acoustofluidic Salivary Exosome Isolation <i>A Liquid Biopsy Compatible Approach for Human Papillomavirus</i>-<i>Associated Oropharyngeal Cancer Detection</i>." JOURNAL OF MOLECULAR DIAGNOSTICS **22**(1): 50-59.

Wei, D., et al. (2023). "Targeted Phosphoproteomics of Human Saliva Extracellular Vesicles via Multiple Reaction Monitoring Cubed (MRM3)." Analytical Chemistry.

Winck, F. V., et al. (2015). "Insights into immune responses in oral cancer through proteomic analysis of saliva and salivary extracellular vesicles." Scientific Reports **5**.

Wu, L., et al. (2022). "Oral Cancer Stem Cell-Derived Small Extracellular Vesicles Promote M2 Macrophage Polarization and Suppress CD4<SUP>+</SUP> T-Cell Activity by Transferring UCA1 and Targeting LAMC2." STEM CELLS INTERNATIONAL **2022**.

Wu, Y., et al. (2024). "Bioorthogonal surface-edited exosomes for targeted multimodal imaging and synergic chemophototherapy of oral cancer." Chemical Engineering Journal **497**: 154653.

Wu, Y. K., et al. (2022). "N-Glycomic profiling reveals dysregulated glycans related to oral cancer using MALDI-MS." ANALYTICAL AND BIOANALYTICAL CHEMISTRY **414**(5): 1881-1890.

Xia, H.-F., et al. (2025). "PCBP2-dependent secretion of miRNAs via extracellular vesicles contributes to the EGFR-driven angiogenesis." Theranostics **15**(4): 1255-1271.

Xiao, B. L., et al. (2023). "HRS Regulates Small Extracellular Vesicle PD-L1 Secretion and Is Associated with Anti-PD-1 Treatment Efficacy." Cancer immunology research **11**(2): 228-240.

Xiao, M., et al. (2018). "M1-like tumor-associated macrophages activated by exosome-transferred THBS1 promote malignant migration in oral squamous cell carcinoma." JOURNAL OF EXPERIMENTAL & CLINICAL CANCER RESEARCH **37**.

Xie, C., et al. (2019). "Exosomes derived from microRNA-101-3p-overexpressing human bone marrow mesenchymal stem cells suppress oral cancer cell proliferation, invasion, and migration." MOLECULAR AND CELLULAR BIOCHEMISTRY **458**(1): 11-26.

Xie, C., et al. (2024). "Exosomal miR-17-5p derived from epithelial cells is involved in aberrant epithelium-fibroblast crosstalk and induces the development of oral submucosal fibrosis." International Journal of Oral Science **16**(1).

Xu, J., et al. (2023). "CCR7 Mediated Mimetic Dendritic Cell Vaccine Homing in Lymph Node for Head and Neck Squamous Cell Carcinoma Therapy." Advanced Science **10**(17).

Xu, K. Y., et al. (2022). "Altered protein profile of plasma extracellular vesicles in oral squamous cell carcinoma development." JOURNAL OF PROTEOMICS **251**.

Xu, Q., et al. (2024). "The small extracellular vesicle‐mediated intercellular transformation of CXCR1<sup>Low</sup> to CXCR1<sup>High</sup> tumour cells promotes the progression of head and neck squamous cell carcinoma." Journal of Extracellular Vesicles **13**(4).

Yadav, J., et al. (2024). "Exosomal transcript cargo and functional correlation with HNSCC patients’ survival." BMC Cancer **24**(1).

Yadav, J., et al. (2025). "Influence of head and neck cancer exosomes on macrophage polarization." Cytokine **186**: 156831.

Yamana, K., et al. (2022). "Extracellular vesicles derived from radioresistant OSCC cells contribute acquired radioresistance via miR-503-3p." CANCER SCIENCE **113**: 1606-1606.

Yan, Q., et al. (2024). "Tumor-associated macrophage-derived exosomal miR21-5p promotes tumor angiogenesis by regulating YAP1/HIF-1α axis in head and neck squamous cell carcinoma." Cellular and Molecular Life Sciences **81**(1).

Yan, W., et al. (2021). "Exosomal miR-130b-3p Promotes Progression and Tubular Formation Through Targeting PTEN in Oral Squamous Cell Carcinoma." FRONTIERS IN CELL AND DEVELOPMENTAL BIOLOGY **9**.

Yang, M., et al. (2022). "Improving the diagnostic efficacy of squamous cell carcinoma antigen for oral squamous cell carcinoma via saponin disruption of serum extracellular vesicles." CLINICA CHIMICA ACTA **525**: 40-45.

Yang, M., et al. (2021). "Bitter melon derived extracellular vesicles enhance the therapeutic effects and reduce the drug resistance of 5-fluorouracil on oral squamous cell carcinoma." JOURNAL OF NANOBIOTECHNOLOGY **19**(1).

Yang, R., et al. (2024). "Radiation‐induced exosomes promote oral squamous cell carcinoma progression via enhancing <scp>SLC1A5</scp>‐glutamine metabolism." Journal of Oral Pathology & Medicine **53**(7): 458-467.

Yang, Z., et al. (2025). "Iron Knights with Nanosword Induced Ferroptosis in the Battle Against Oral Carcinoma." Nano Letters **25**(1): 327-335.

Yang, Z. J., et al. (2021). "A New Nanomaterial Based on Extracellular Vesicles Containing Chrysin-Induced Cell Apoptosis Through Let-7a in Tongue Squamous Cell Carcinoma." FRONTIERS IN BIOENGINEERING AND BIOTECHNOLOGY **9**.

Ye, B. B., et al. (2023). "Hypoxic tumor-derived exosomal miR-21 induces cancer-associated fibroblast activation to promote head and neck squamous cell carcinoma metastasis." CELLULAR SIGNALLING **108**.

You, D. Y., et al. (2023). "MiR-3529-3p from PDGF-BB-induced cancer-associated fibroblast-derived exosomes promotes the malignancy of oral squamous cell carcinoma." DISCOVER ONCOLOGY **14**(1).

You, Y. H., et al. (2023). "Identification of Exosome-Related Genes Associated with Prognosis and Immune Infiltration Features in Head-Neck Squamous Cell Carcinoma." BIOMOLECULES **13**(6).

Yu, Z.-L., et al. (2024). "Predictive Analysis in Oral Cancer Immunotherapy: Profiling Dual PD-L1-Positive Extracellular Vesicle Subtypes with Step-Wedge Microfluidic Chips." Analytical Chemistry **96**(37): 14980-14988.

Yuan, Y., et al. (2021). "Macrophage-Derived Exosomal miR-31-5p Promotes Oral Squamous Cell Carcinoma Tumourigenesis Through the Large Tumor Suppressor 2-Mediated Hippo Signalling Pathway." Journal of biomedical nanotechnology **17**(5): 822-837.

Yuana, Y., et al. (2017). "Microbubbles-assisted ultrasound triggers the release of extracellular vesicles." International Journal of Molecular Sciences **18**(8).

Yunusova, N. V., et al. (2017). The Characterization of Exosomes from Biological Fluids of Patients with Different Types of Cancer. PHYSICS OF CANCER: INTERDISCIPLINARY PROBLEMS AND CLINICAL APPLICATIONS (PC IPCA). **1882**.

Zhang, M. J., et al. (2023). "Exosomes derived from oral squamous cell carcinoma tissue accelerate diabetic wound healing." AMERICAN JOURNAL OF PHYSIOLOGY-CELL PHYSIOLOGY **324**(6): C1307-C1319.

Zhang, S., et al. (2025). "Cancer-associated fibroblasts promote oral squamous cell carcinoma progression by targeting ATP7A via exosome-mediated paracrine miR-148b-3p." Cellular Signalling **128**: 111631.

Zhang, Y., et al. (2020). "Exosomal CircGDI2 Suppresses Oral Squamous Cell Carcinoma Progression Through the Regulation of MiR-424-5p/SCAI Axis." CANCER MANAGEMENT AND RESEARCH **12**: 7501-7514.

Zhao, J., et al. (2025). "Salivary apoptotic microvesicles as biomarkers for prognostic non-healing oral ulcers and oral cancer: a cross-sectional study." Scientific Reports **15**(1).

Zheng, Y., et al. (2020). "Identification of extracellular vesicles-transported miRNAs in Erlotinib-resistant head and neck squamous cell carcinoma." JOURNAL OF CELL COMMUNICATION AND SIGNALING **14**(4): 389-402.

Zhong, W. Q., et al. (2019). "Increased salivary microvesicles are associated with the prognosis of patients with oral squamous cell carcinoma." Journal of Cellular and Molecular Medicine **23**(6): 4054-4062.

Zhou, J., et al. (2024). "Extracellular vesicle-bound VEGF in oral squamous cell carcinoma and its role in resistance to Bevacizumab Therapy." Cancer Cell International **24**(1).

Zhou, S., et al. (2021). "Exosome-derived long non-coding RNA ADAMTS9-AS2 suppresses progression of oral submucous fibrosis via AKT signalling pathway." J Cell Mol Med **25**(4): 2262-2273.

Zhou, Z. H., et al. (2024). "Macrophage‐Capturing Self‐Assembly Photosensitizer Nanoparticles Induces Immune Microenvironment Re‐Programming and Golgi‐Responsive Immunogenic Cell Death in Head and Neck Carcinoma." Advanced Healthcare Materials **13**(20).

Zhu, F., et al. (2021). "High-Throughput Single-Cell Extracellular Vesicle Secretion Analysis on a Desktop Scanner without Cell Counting." Analytical Chemistry **93**(39): 13152-13160.

Zhu, G., et al. (2021). "Small extracellular vesicles containing miR-192/215 mediate hypoxia-induced cancer-associated fibroblast development in head and neck squamous cell carcinoma." Cancer Letters **506**: 11-22.

Zhu, G., et al. (2021). "Loxl2-enriched small extracellular vesicles mediate hypoxia-induced premetastatic niche and indicates poor outcome of head and neck cancer." Theranostics **11**(19): 9198-9216.

Zhu, G., et al. (2023). "90 K increased delivery efficiency of extracellular vesicles through mediating internalization." Journal of Controlled Release **353**: 930-942.

Zhu, X., et al. (2020). "Oral cancer cell‑derived exosomes modulate natural killer cell activity by regulating the receptors on these cells." International Journal of Molecular Medicine **46**(6): 2115-2125.

Zhuang, D. X., et al. (2022). "Phenformin suppresses angiogenesis through the regulation of exosomal microRNA-1246 and microRNA-205 levels derived from oral squamous cell carcinoma cells." FRONTIERS IN ONCOLOGY **12**.

Zlotogorski-Hurvitz, A., et al. (2019). "FTIR-based spectrum of salivary exosomes coupled with computational-aided discriminating analysis in the diagnosis of oral cancer." JOURNAL OF CANCER RESEARCH AND CLINICAL ONCOLOGY **145**(3): 685-694.

Zorrilla, S. R., et al. (2019). "A pilot clinical study on the prognostic relevance of plasmatic exosomes levels in oral squamous cell carcinoma patients." Cancers **11**(3).
